# Supplementary material for: Locking CBL TKBD in its native conformation presents a novel therapeutic opportunity in mutant CBL-dependent leukemia
Source: Mol Ther. 2025 May 5;33(8):3624–43. doi: 10.1016/j.ymthe.2025.04.042 (PMC12461627; doi:10.1016/j.ymthe.2025.04.042)
Supplement: Supplementary file 1 [file mmc1.pdf]

YMTHE, Volume *33*

Supplemental Information

Locking CBL TKBD in its native conformation presents a novel therapeutic opportunity in mutant CBL-dependent leukemia

Syed Feroj Ahmed, Jayanthi Anand, Wei Zhang, Lori Buetow, Loveena Rishi, Louise Mitchell, Jonathan Bohlen, Sergio Lilla, Gary J. Sibbet, Colin Nixon, Amrita Patel, Karolina A. Majorek, Sara Zanivan, Jacinta C. Bustamante, Sachdev S. Sidhu, Karen Blyth, and Danny T. Huang

Supplemental Information

Materials and Methods

Estimation of iRFP–CBLock variant concentration in cells

The MOLM-13$^{Luc/CBLock}$ variant cells were induced with 2 µg/ml dox on every 2 days and cells from day 1, 3, 5 and 8 were lysed. The cell lysates (2.0 µl) from three independent experiments from each day for each iRFP–CBLock variant were separated by SDS-PAGE using 4–12%NuPAGE™ Bis-Tris gels (Thermo Fisher Scientific). Purified iRFP–CBLock (25 µM) was run as a standard in each gel. Following separation, the gels were scanned using an Odyssey CLx Imaging System (LI-COR). The bands were quantified using the LI-COR Image Studio 6.0 software and normalized against the whole-cell lysate concentration. The cell pellet for each sample yielded 50 µl whole-cell lysate. Concentration of iRFP–CBLock variant was calculated using the formula:

Concentration (µM) = [{(Normalized band intensity of iRFP–CBLock variant/Band intensity of purified iRFP–CBLock) x Concentration of purified iRFP–CBLock (µM)} x total volume of cell lysate]/ total cell volume.

Volume of $5.5x10^6$ MOLM-13 cells were calculated to be ~10 µl, considering an average diameter of 15 µm for each cell and using the formula $4/3\pi r^3$

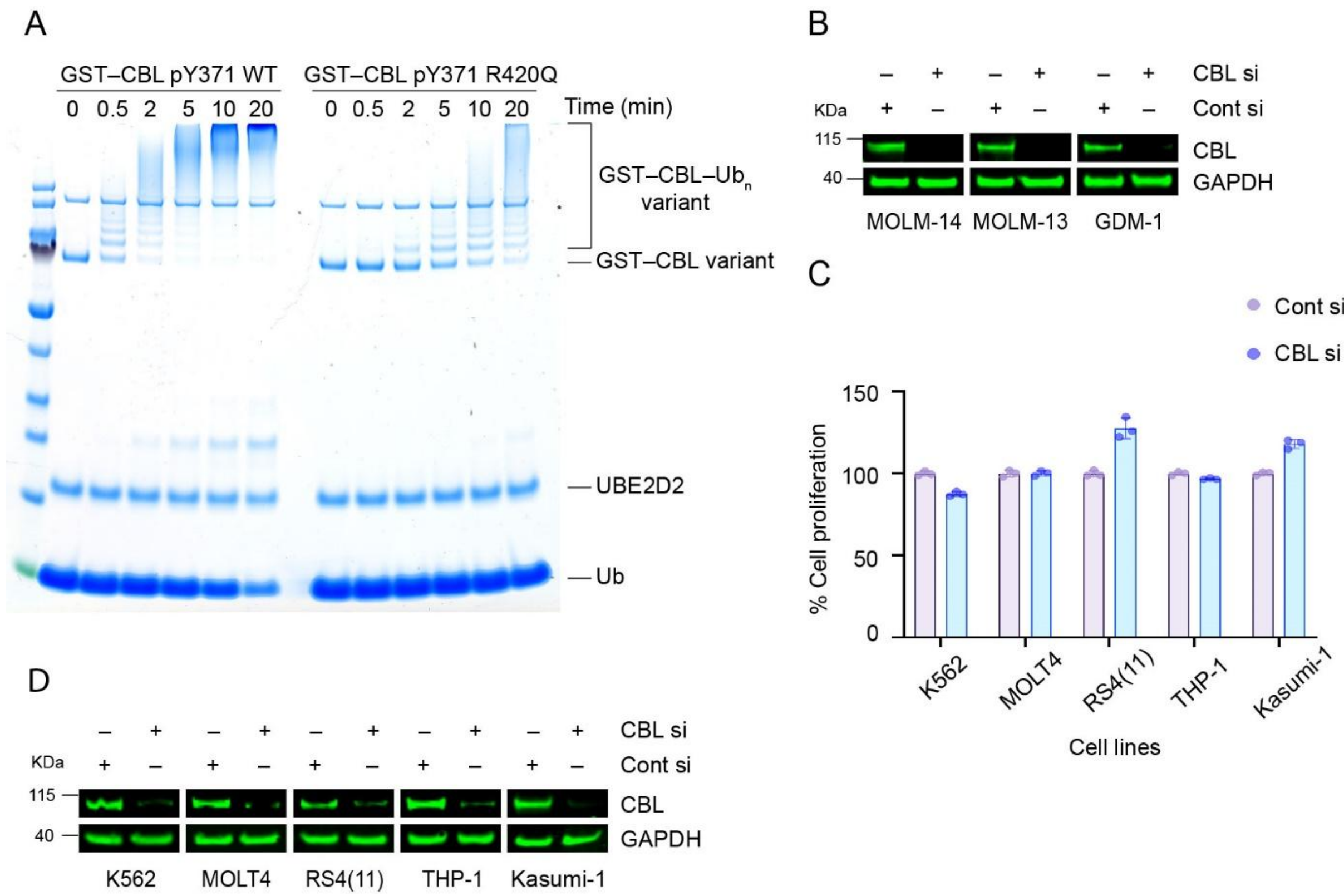

Fig S1. CBL-WT cannot provide growth advantage to leukemia cells. (A) Representative Coomassie stained gel showing the *in vitro* auto-ubiquitination assay performed with purified GST-pCBL 47-435 or GST-pCBL 47-435 R420Q. Three independent experiments were performed. (B) Related to Figure 1D, expression of CBL and GAPDH (loading control) in lysates from MOLM-14, MOLM-13 and GDM-1 cells transfected with control siRNA (Cont si) or CBL siRNA (CBL si). (C) Proliferation of leukemia cells K562, MOLT4, RS4(11), THP-1, and Kasumi-1 knocked down with control siRNA (Cont si) or CBL siRNA (CBL si) and analyzed after 48 hours using Cell Titer-Glo and represented as percentage of cells knocked down with cont si. The data are presented from three independent experiments while the error bars represent ±SD (n = 3 independent experiments). (D) Expression of CBL and GAPDH (loading control) in lysates from K562, MOLT4, RS4(11), THP-1, and Kasumi-1 cell lines transfected with control siRNA (Cont si) or CBL siRNA (CBL si). The GAPDH panel is a loading control in B and D.

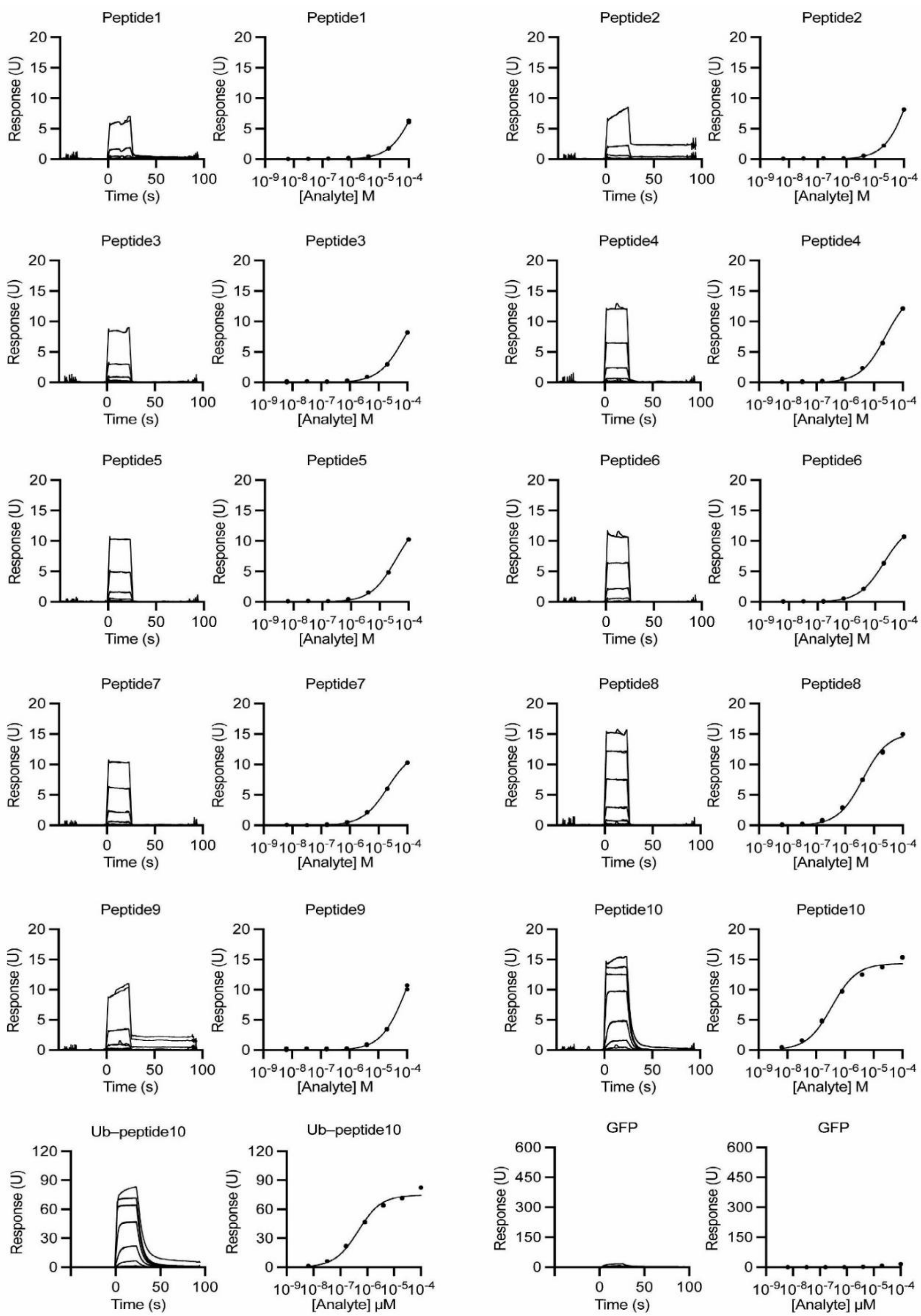
Peptide1
Peptide2
Peptide3
Peptide4
Peptide5
Peptide6
Peptide7
Peptide8
Peptide9
Peptide10
Ub–peptide10
GFP
Response (U)
Time (s)
[Analyte] M
[Analyte] μM

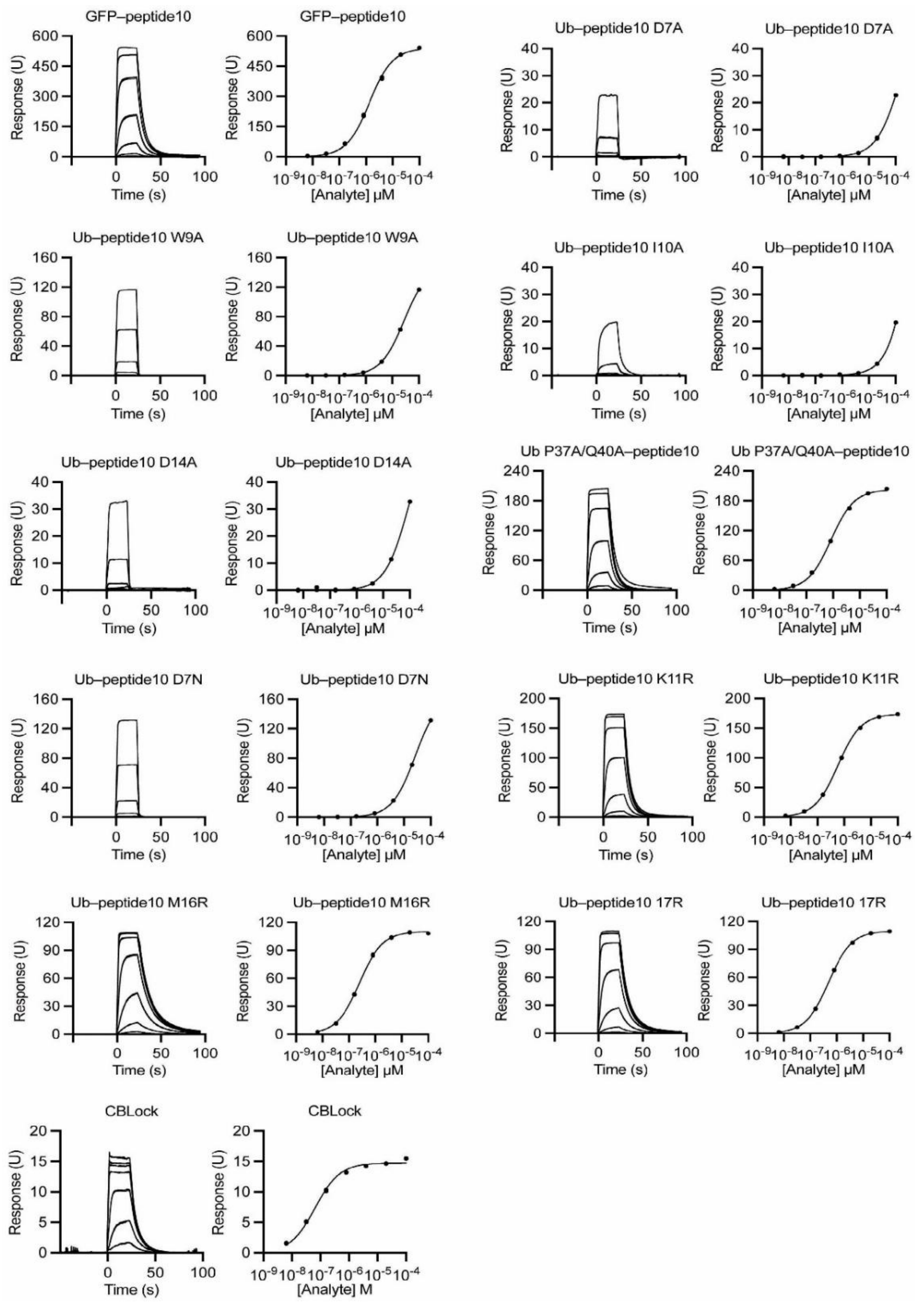

Fig S2. SPR analyses of GST-bound ligand and analyte binding affinities, related to Table 1. Representative sensorgrams (left) and binding curves (right) for GST-TKBD and the analytes peptide (1-10), Ub-peptide10, GFP, GFP-peptide10, Ub-peptide10 D7A, Ub-peptide10 W9A, Ub-peptide10 I10A, Ub-peptide10 D14A, Ub-P37A/Q40A-peptide10, Ub-peptide10 D7N, Ub-peptide10 K11R, Ub-peptide10 M16R, Ub-peptide10 17R, CBLock, as indicated above each sensorgram and binding curve set. Two technical replicates (n =2) for each binding curve.

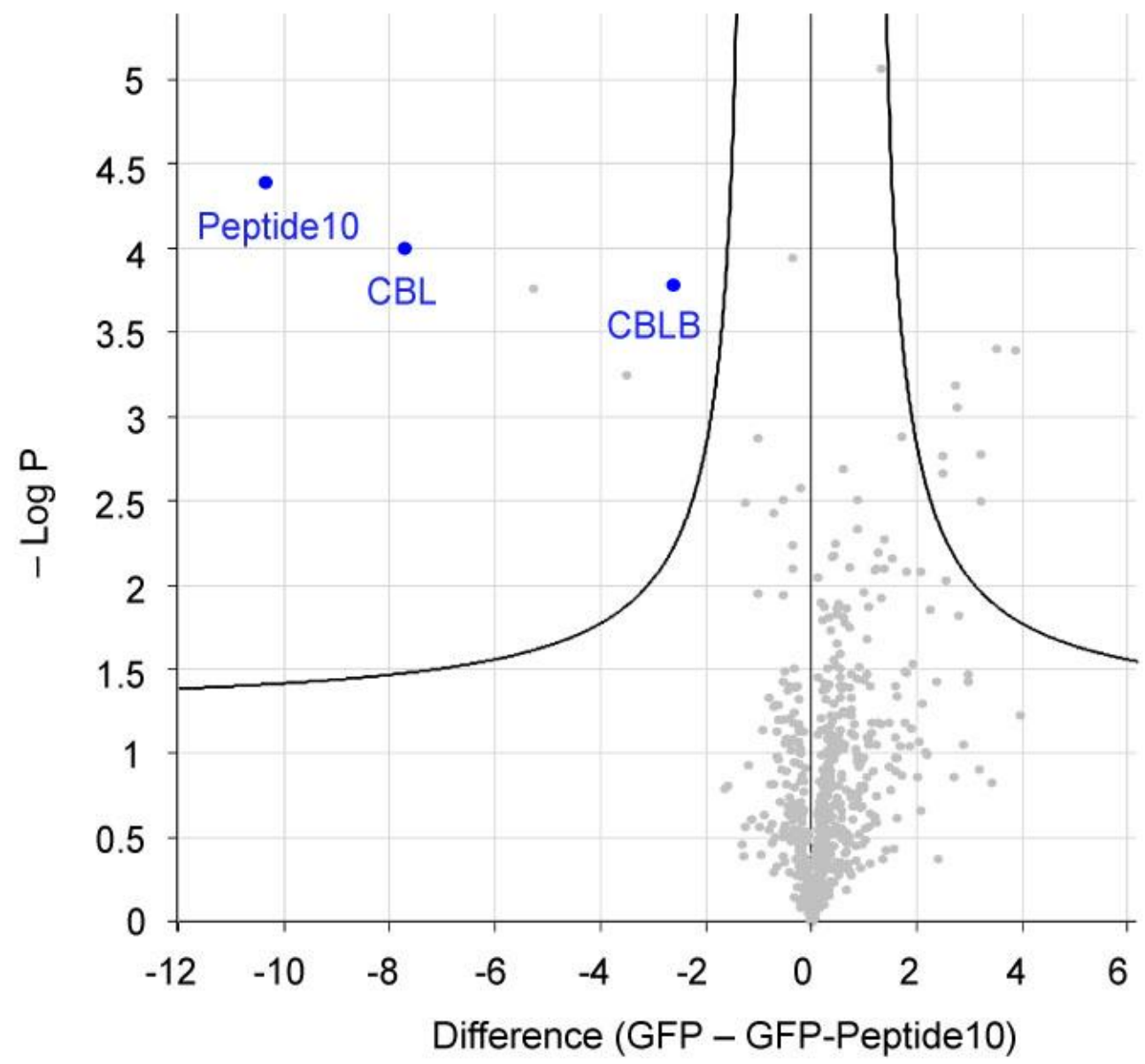

Fig S3. Peptide10 binds specifically to CBL in cells. Volcano plot showing differences in the interactomes of GFP and GFP-peptide10. The experiment was performed in three biological replicates.

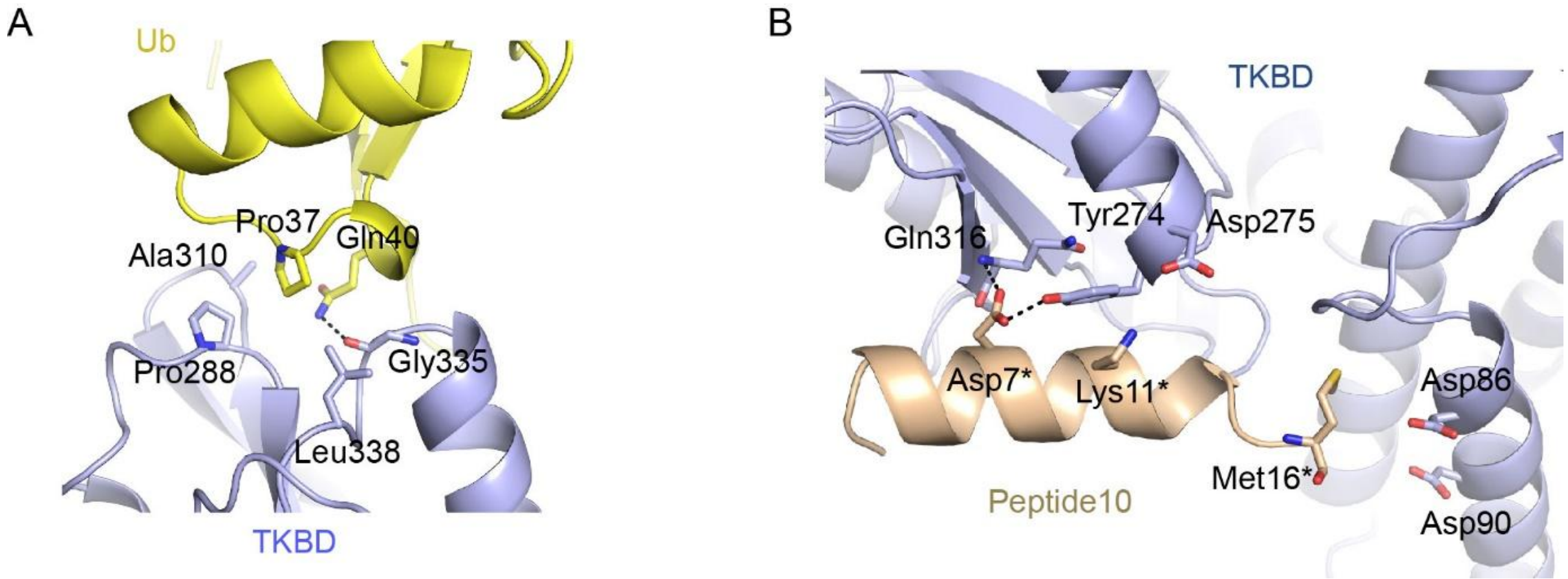

Fig S4. Close-up views of binding interfaces in the CBL TKBD-Ub-peptide10 complex structure (A) Close-up view of CBL TKBD-Ub binding interface. Key interacting residues are shown as sticks. Coloring as in Figure 2A. O and N atoms are colored red and blue, respectively. (B) Close-up view of CBL TKBD-peptide10 binding interface. Residues and regions for potential sequence optimization to improve binding affinity are shown as sticks. Coloring as in Figure 2A. O and N atoms are colored red and blue, respectively.

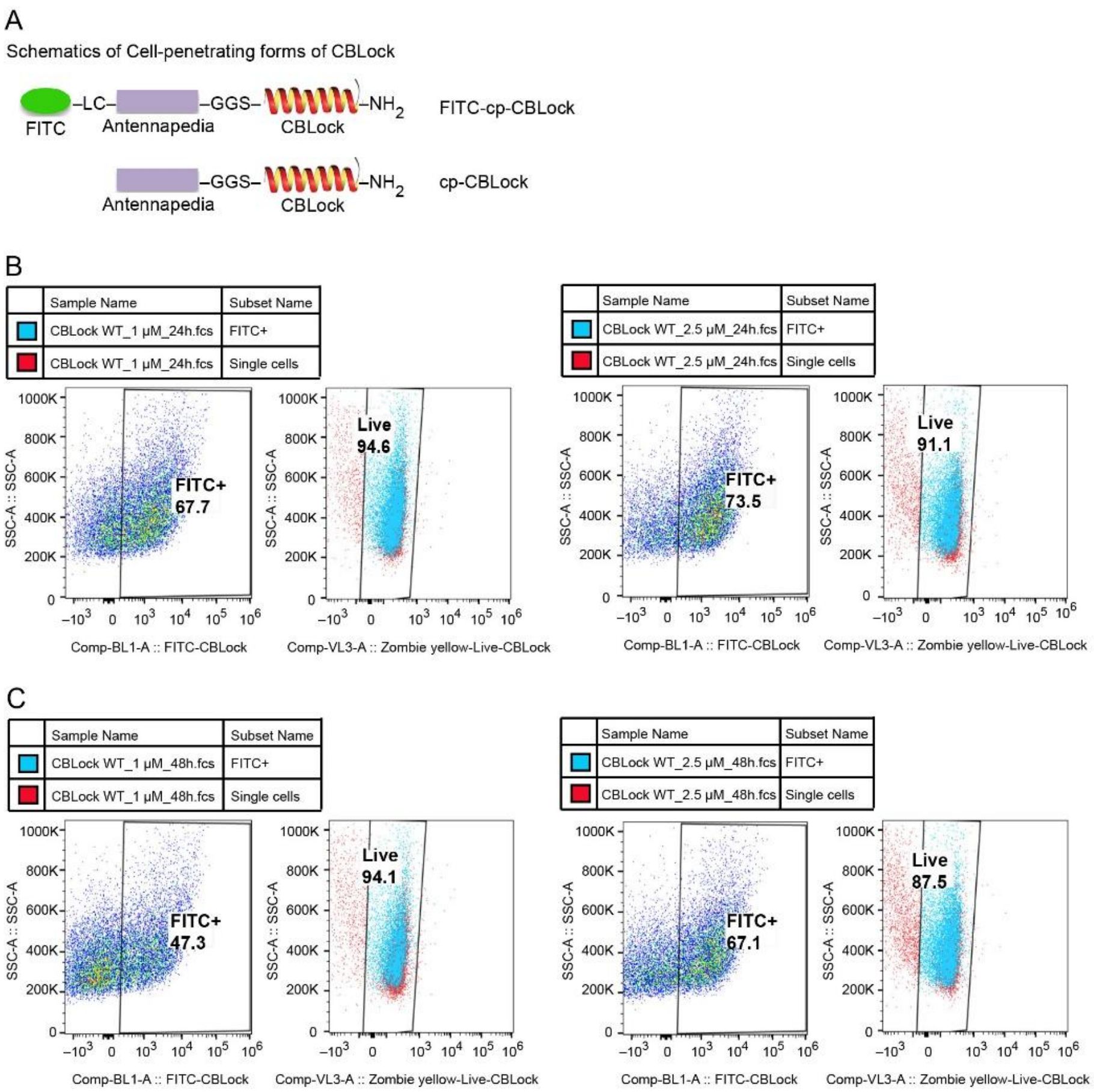

Fig S5. Cell penetrating form of CBLock. (A) Schematics outlining the CBLock with antennapedia (cp-CBLock) with/without FITC conjugation. (B) FACS analysis of MOLM-14 cells treated with FITC-cp-CBLock (1 and 2.5 µM) for 24 hours and 48 hours. Zombie yellow was used to find the dead cells. Non-treated cells were used as fluorescence negative controls to set gating. The experiment was performed in three biological replicates.

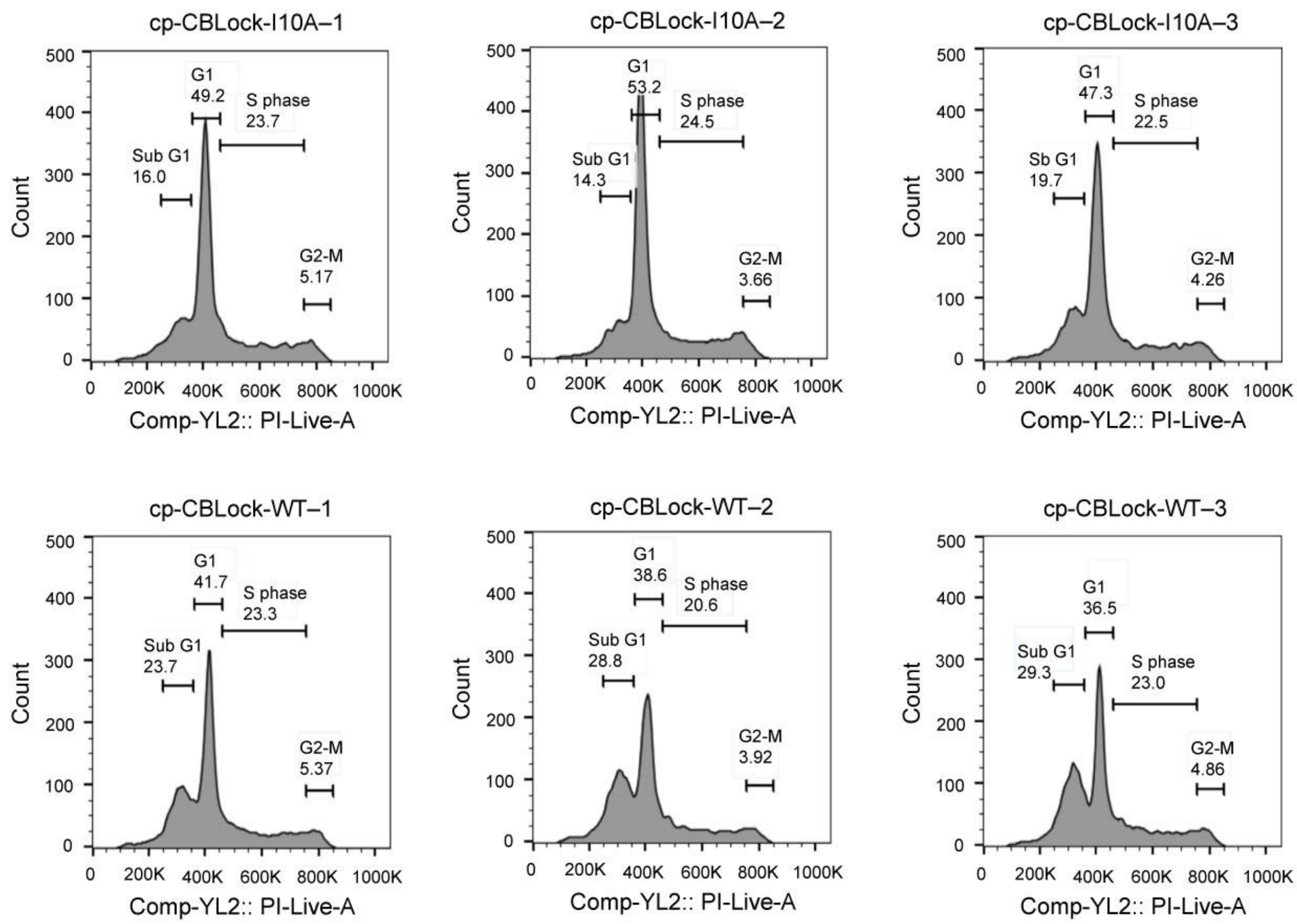

Fig S6. CBLock treatment causes sub-G1 cell cycle arrest in mutant CBL cells. Cell cycle analysis of MOLM-14 cells treated with 2.5 µM cp-CBLock WT or cp-CBLock I10A for 48 hours. Representative examples of data shown in Figure 4B. Vehicle treated cells were used as the controls. Histograms show the cell cycle profile of the cells from the 3 independent experiments stained with PI.

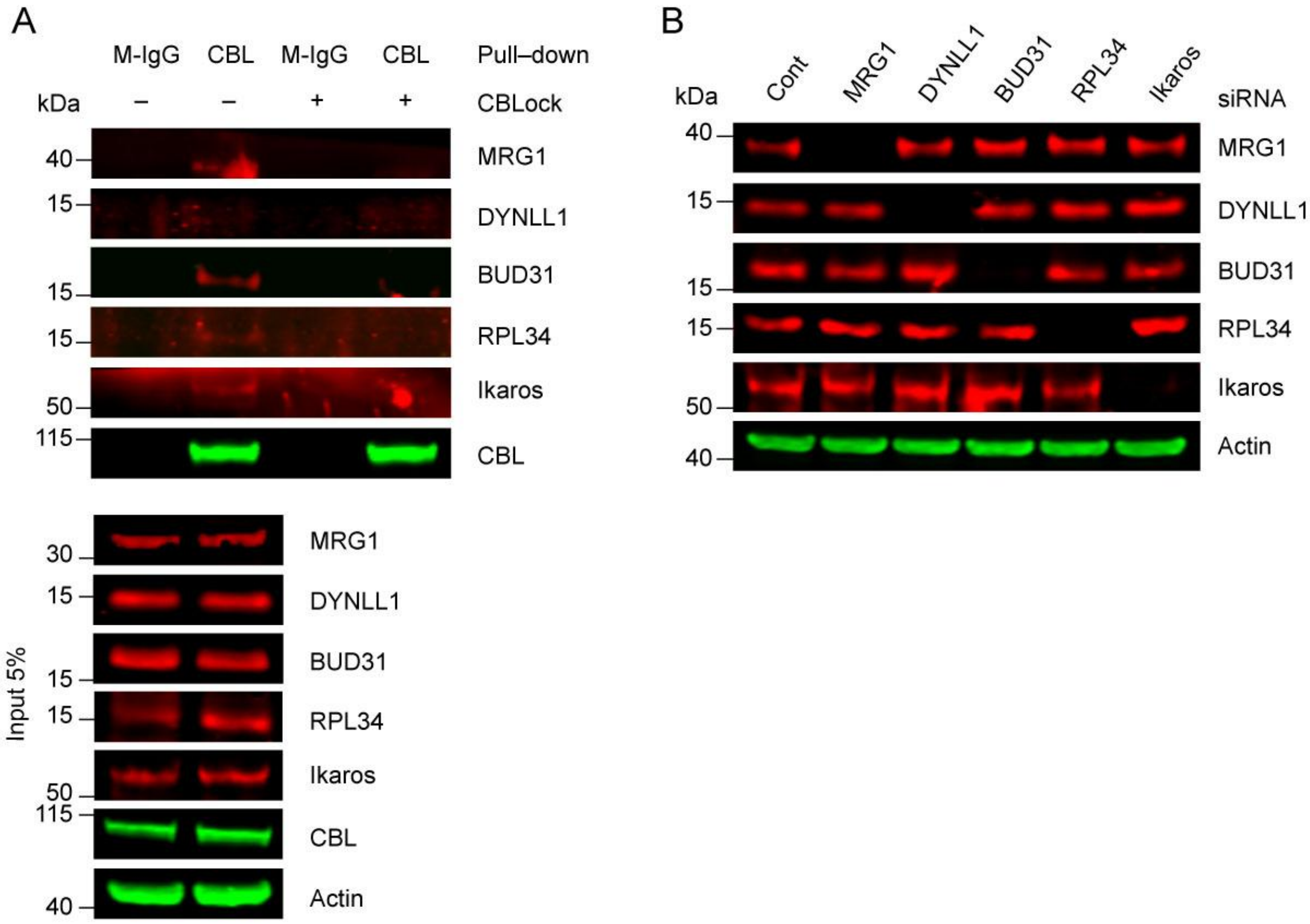

Fig S7. CBLock disrupts additional mutant CBL interactome. (A) Co-immunoprecipitation assay of MOLM-14 cell lysates treated with vehicle (water) or 5 µM cp-CBLock. Anti-CBL antibody and mouse anti-IgG (M-IgG) immunoprecipitants and cell lysates were analyzed by immunoblotting with anti-MRG1, anti-DYNLL1, anti-BUD31, anti-RPL34, anti-Ikaros, anti-CBL and anti-Actin antibodies as indicated. Related to Figure 4H. (B) Related to Figure 4I, expression of MRG1, DYNLL1, BUD31, RPL34, Ikaros and Actin in lysates from MOLM-14 cells transfected with control siRNA (Cont si), MRG1 siRNA (MRG1 si), DYNLL1 siRNA (DYNLL1 si), BUD31 siRNA (BUD31 si), RPL34 siRNA (RPL34 si), or Ikaros siRNA (Ikaros si). The Actin panel is a sample control in A and B.

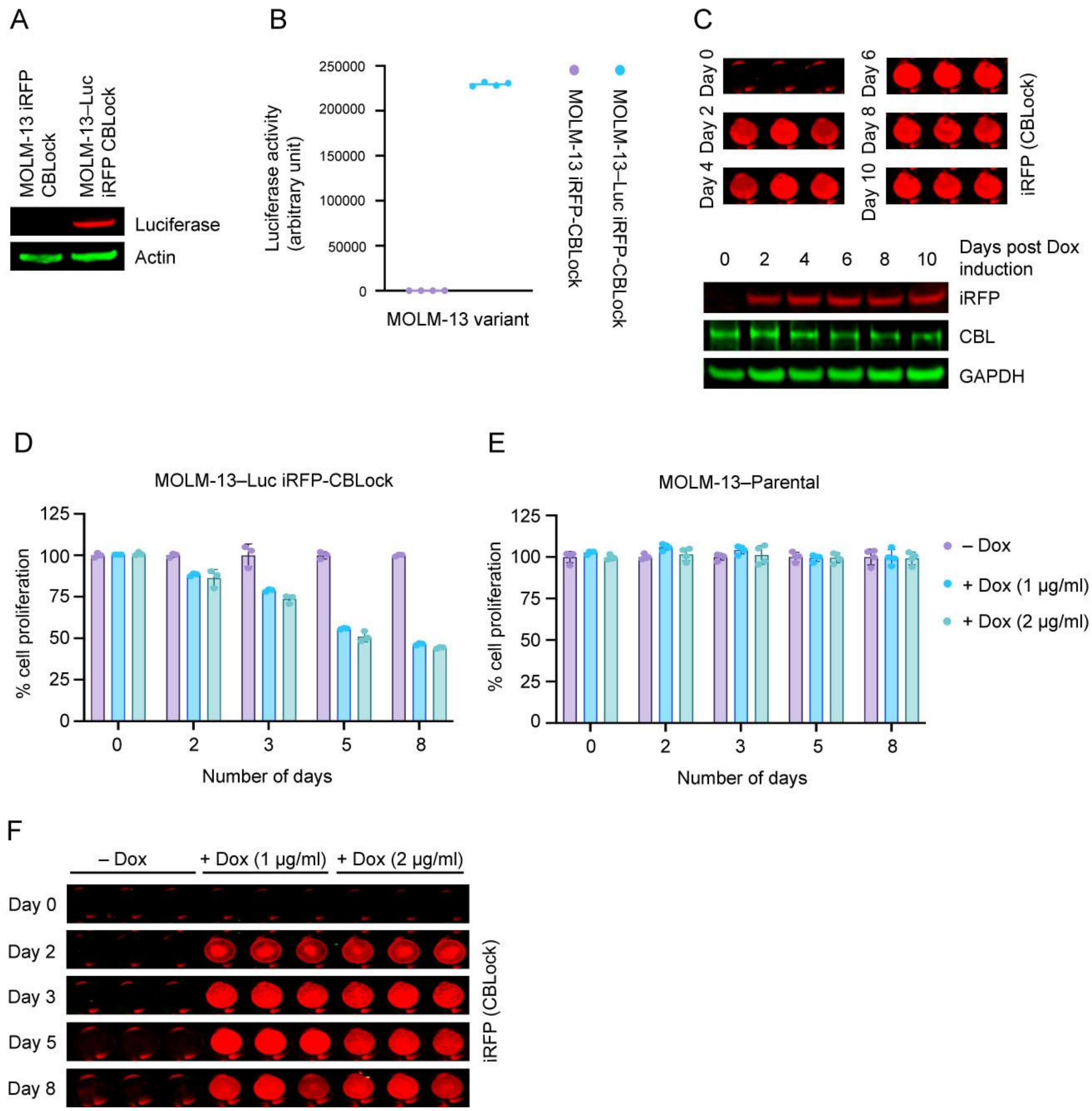
A
MOLM-13 iRFP CBLock
MOLM-13–Luc iRFP CBLock
Luciferase
Actin
B
Luciferase activity (arbitrary unit)
250000
200000
150000
100000
50000
0
MOLM-13 variant
MOLM-13 iRFP-CBLock
MOLM-13–Luc iRFP-CBLock
C
Day 0
Day 2
Day 4
Day 6
Day 8
Day 10
iRFP (CBLock)
Days post Dox induction
0 2 4 6 8 10
iRFP
CBL
GAPDH
D
MOLM-13–Luc iRFP-CBLock
% cell proliferation
125
100
75
50
25
0
0 2 3 5 8
Number of days
E
MOLM-13–Parental
% cell proliferation
Number of days
– Dox
+ Dox (1 µg/ml)
+ Dox (2 µg/ml)
F
– Dox
+ Dox (1 µg/ml)
+ Dox (2 µg/ml)
Day 0
Day 2
Day 3
Day 5
Day 8
iRFP (CBLock)

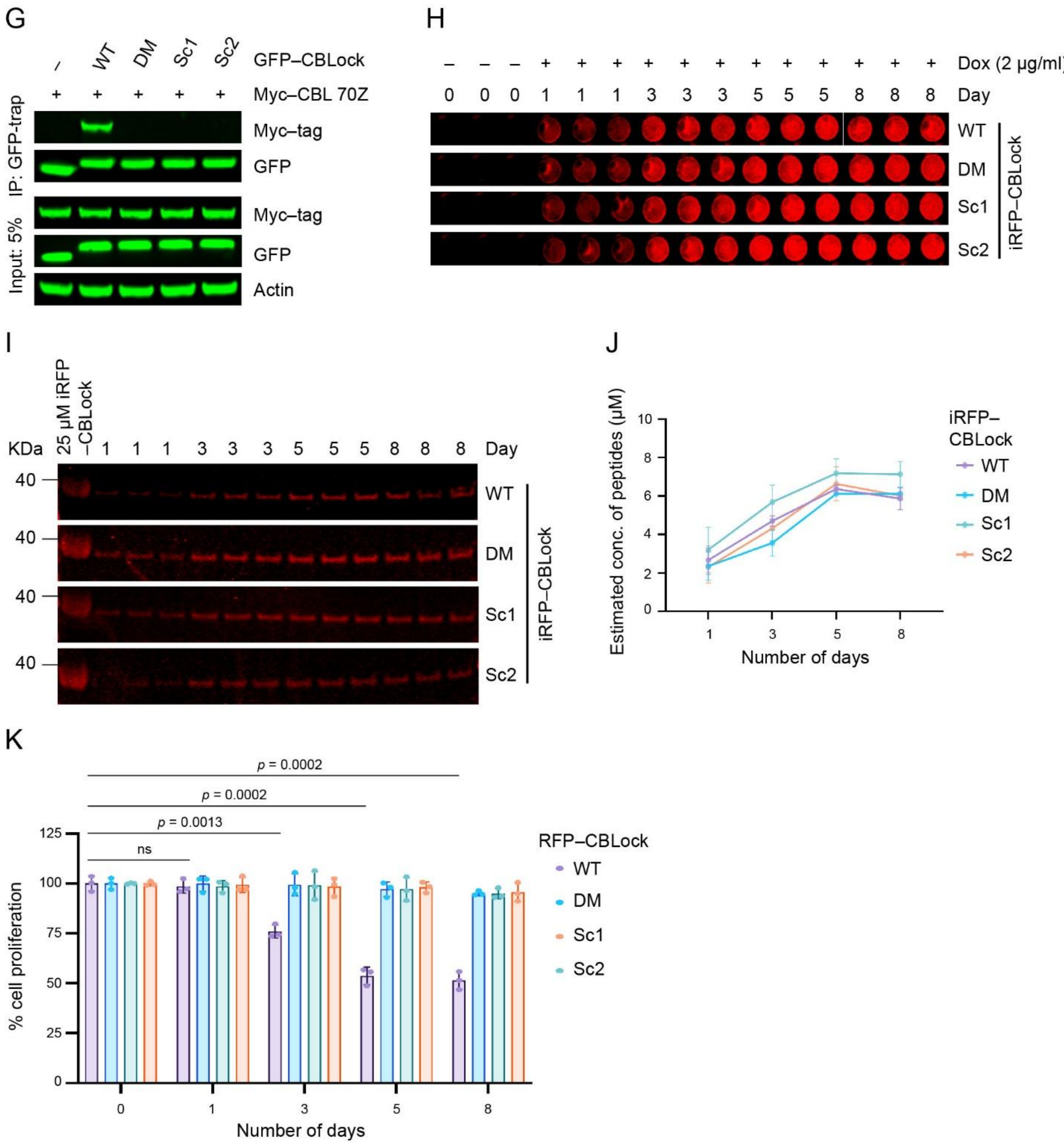

Fig S8. Developing constitutive luciferase expressing and inducible iRFP-CBLock variant expressing MOLM-13 cells. (A) Expression of luciferase and Actin were analyzed from lysates of MOLM-13^{CBLock} or MOLM-13$^{Luc/CBLock}$ cells using anti-luciferase and anti-Actin antibodies, where Actin is the loading control. (B) Luciferase activity of MOLM-13^{CBLock} or MOLM-13$^{Luc/CBLock}$ cells using One-Glo assay system (n=4). The data points are presented from four biological replicates (n = 4 biological replicates). (C) Dox induced expression of iRFP-CBLock. MOLM-13$^{Luc/CBLock}$ cells were treated with 2 µg/ml dox every two days for 10 days. Cells from day 0, 2, 4, 6, 8, 10 were scanned using the LI-COR imaging system. Alternatively, lysates from dox induced cells were lysed and checked for iRFP (CBLok), CBL and GAPDH expressions,

where GAPDH is used as the loading control. (D) Proliferation of MOLM-$13^{Luc/CBLock}$ cells treated with dox (1 and 2 μg/ml) every 2 days for 8 days and cells from 0, 2, 3, 5, and 8 days were analyzed using Cell Titer-Glo and represented as percentage of cells on day 0. The data points are presented from three independent experiments while the error bars represent ±SD (n = 3 independent experiments). (E) Proliferation of MOLM-$13^{parental}$ cells treated with dox (1 and 2 μg/ml) as in D. The data points are presented from four independent experiments while the error bars represent ±SD (n = 4 independent experiments). (F) iRFP (CBLock) expression from cells in D. (G) Co-immunoprecipitation assay of HEK293 cell lysates overexpressing Myc-tagged CBL-70Z along with GFP or GFP-CBLock variants as indicated in the figure. GFP-trap immunoprecipitants and cell lysates were analyzed by immunoblotting with anti-Myc–tag, anti-GFP and anti-Actin antibodies as indicated. (H) iRFP (CBLock variant) expression from MOLM-13^{Luc}cells expressing the respective variant in presence of 2 μg/ml dox from days post induction as indicated in the figure. (I) Dox-induced cells in H were lysed and lysates were checked for iRFP (CBLock variants). Purified iRFP–CBLock (25 μM) was run as a standard. (J) Plot showing the estimated concentrations (in μM) of iRFP–CBLock variants. The data points represent the mean values from three independent experiments while the error bars represent ±SD (n = 3 independent experiments). (K) Proliferation of MOLM-$13^{Luc/CBLock}$ variant cells treated with dox (2 μg/ml) every 2 days for 8 days and cells from 0, 1, 3, 5, and 8 days were analyzed using Cell Titer-Glo and represented as percentage of cells on day 0. The data points are presented from three independent experiments while the error bars represent ±SD (n = 3 independent experiments).

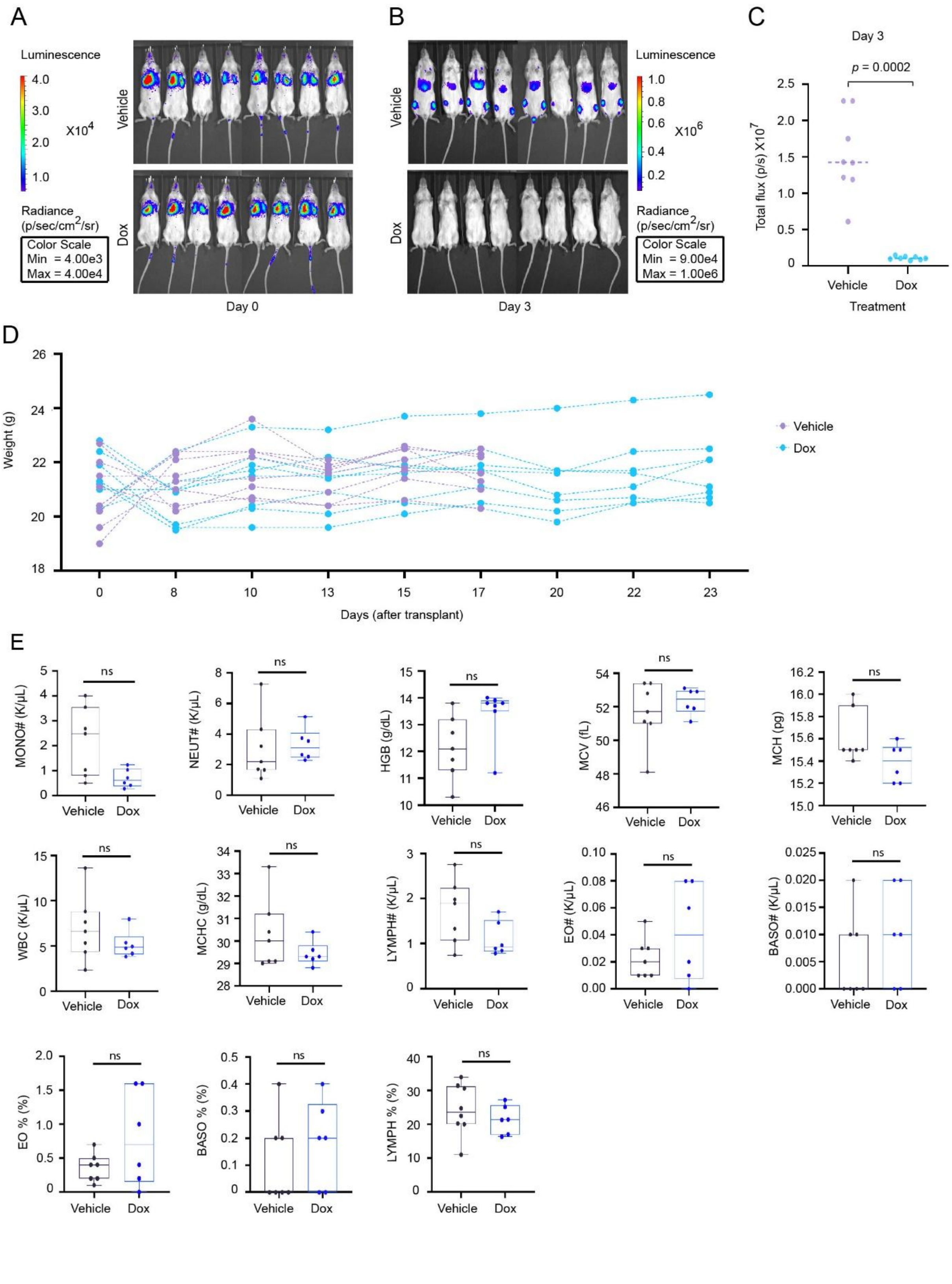

A
Luminescence
4.0
3.0
2.0
1.0
X10^4
Radiance (p/sec/cm^2/sr)
Color Scale Min = 4.00e3 Max = 4.00e4
Vehicle
Dox
Day 0
B
Vehicle
Dox
Luminescence
1.0
0.8
0.6
0.4
0.2
X10^6
Radiance (p/sec/cm^2/sr)
Color Scale Min = 9.00e4 Max = 1.00e6
Day 3
C
Day 3
p = 0.0002
Total flux (p/s) X10^7
Vehicle
Dox
Treatment
D
Weight (g)
Days (after transplant)
Vehicle
Dox
E
MONO# (K/μL)
NEUT# (K/μL)
HGB (g/dL)
MCV (fL)
MCH (pg)
WBC (K/μL)
MCHC (g/dL)
LYMPH# (K/μL)
EO# (K/μL)
BASO# (K/μL)
EO % (%)
BASO % (%)
LYMPH % (%)
ns
Vehicle
Dox

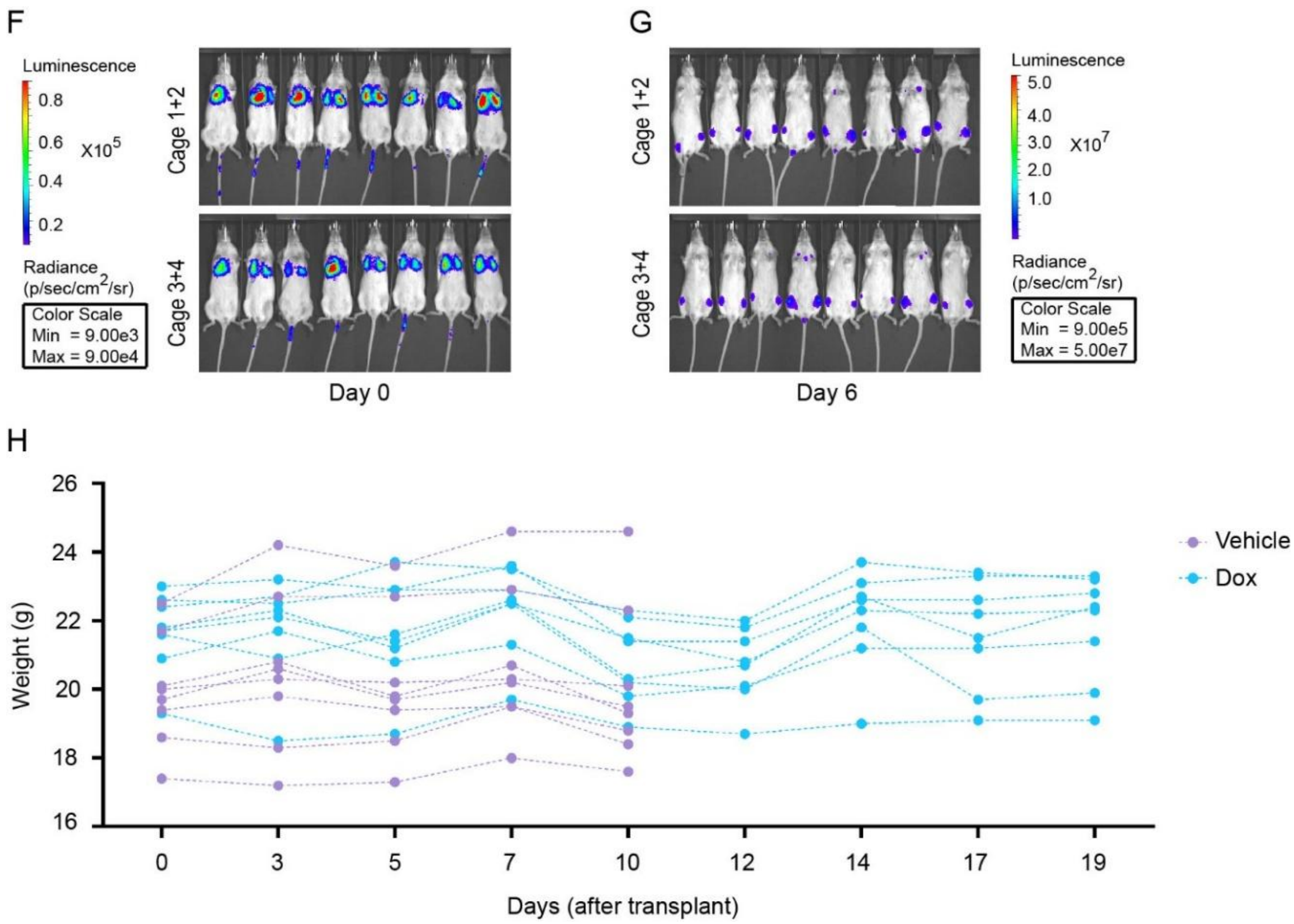

Fig S9. CBLock delays tumour progression in the mouse model of AML. (A) Bioluminescence imaging of NSG mice injected with MOLM-13$^{Luc/CBLock}$ cells on the day of cell injection to monitor cell uptake (n=8 mice per group). Relative to Figure 5B. (B) Bioluminescence imaging of NSG mice iv injected with 100 µl of 5x10^{6} MOLM-13$^{Luc/CBLock}$ cells and treated with either vehicle (n = 8) or dox (n = 8) to regularly track disease progression, related to Figure 5B. (C) Tumor burdens in both groups of mice (shown in B) were measured as total flux (p/s). Total flux (p/s) for the ventral view of the mice were calculated from the region of interest (ROI) of the bioluminescent images of the vehicle (n= 8) and dox (n = 8) treated mice, the dashed lines represent median values. (D) Plot showing body weights of individual mice treated with vehicle (n = 8) or dox (n = 8). (E) Complete blood count analyses of vehicle (n=7) and dox (n=6, reason for exclusion of 2 samples provided in the methods section) treated mice cardiac whole blood. Plots showing neutrophil count (NEUT), monocyte count (MONO), hemoglobin (HGB), mean corpuscular volume (MCV), mean corpuscular hemoglobin (MCH), white blood cells (WBC), mean corpuscular hemoglobin concentration (MCHC), lymphocyte count (LYMPH), eosinophil count (EO), basophil count (BASO), eosinophil percent (EO %), basophil percent (BASO %), and lymphocyte percent (LYMPH %) between vehicle and dox treated mouse blood. Data points represent individual mice blood harvested at clinical endpoint (day12) for vehicle (n = 7) and timed endpoint (day 21) for dox (n = 6) treated mice, the error bars represent ±SD. (F) Bioluminescence imaging of NSG mice injected with MOLM-13$^{Luc/CBLock}$ cells on the day of cell injection to monitor cell uptake. Related to Figure 6F. (G) Bioluminescence imaging of NSG mice iv injected with 100 µl of 1x10^{6} MOLM-13$^{Luc/CBLock}$ cells to track disease progression. (H) Plot showing body weights of individual mice treated with vehicle or dox.

Table S1: Data collection and refinement statistics

	TKBD-Ub-peptide10
**Data collection**	
PDB	9ERZ
Space group	$P12_11$
Cell dimensions	
a, b, c (Å)	58.78, 87.32, 84.35
α , β, γ (°)	90, 99.39, 90
Resolution (Å)	60.2–2.02 (2.05–2.02)
R_{merge} (%)	3.8 (50.8)
R_{pim} (%)	2.4 (33.2)
Completeness (%)	97.2 (99.7)
Multiplicity	3.4 (3.3)
I/σI	17.1 (2.2)
CC(1/2)	0.999 (0.835)
Wilson B (Å^2)	37.85
**Refinement**	
R_{work} (%)	17.81
R_{free} (%)	22.91
Number of reflections	54602
No. atoms	
Protein	6064
Water	349
Ligand /ion	2
R.m.s.d.	
Bond lengths (Å)	0.007
Bond angles (°)	0.768
B-factors	
Protein	55.56
Ca^{2+}	74.77
Water	46.39

Values in parentheses are for highest-resolution shell.
