# Supplementary material for: Locking CBL TKBD in its native conformation presents a novel therapeutic opportunity in mutant CBL-dependent leukemia
Source: Mol Ther. 2025 May 5;33(8):3624–43. doi: 10.1016/j.ymthe.2025.04.042 (PMC12461627; doi:10.1016/j.ymthe.2025.04.042)
Supplement: Supplementary file 2 [file mmc2.pdf]

Molecular Therapy
Original Article

Locking CBL TKBD in its native conformation presents a novel therapeutic opportunity in mutant CBL-dependent leukemia

Syed Feroj Ahmed,[1] Jayanthi Anand,[1] Wei Zhang,[2] Lori Buetow,[1] Loveena Rishi,[1,7] Louise Mitchell,[1] Jonathan Bohlen,[3,4] Sergio Lilla,[1] Gary J. Sibbet,[1] Colin Nixon,[1] Amrita Patel,[1,5,8] Karolina A. Majorek,[1] Sara Zanivan,[1,5] Jacinta C. Bustamante,[3,4] Sachdev S. Sidhu,[6] Karen Blyth,[1,5] and Danny T. Huang[1,5]

[1]Cancer Research UK Scotland Institute, Garscube Estate, Switchback Road, Glasgow G61 1BD, UK; [2]Department of Molecular and Cellular Biology, College of Biological Science, University of Guelph, 50 Stone Road E, Guelph, ON N1G2W1, Canada; [3]Laboratory of Human Genetics of Infectious Diseases, Necker Hospital for Sick Children, 75015 Paris, France; [4]Paris Cité University, Imagine Institute, 75015 Paris, France; [5]School of Cancer Sciences, University of Glasgow, Glasgow G61 1QH, UK; [6]School of Pharmacy, University of Waterloo, 10 Victoria Street S A, Kitchener, ON N2G1C5, Canada

Casitas B-lineage lymphoma (CBL) is an E3 ubiquitin ligase critical for negatively regulating receptor protein tyrosine kinases (RTKs). Deleterious CBL mutants lose E3 activity, but act as adaptors that gain function to cause myeloproliferative neoplasms. Currently, there is no targeted treatment available for patients with *CBL* mutant-dependent disorders. By combining phage-display technology and structure-based optimization, we discovered CBLock, a nanomolar affinity peptide inhibitor, that binds the substrate-binding site of CBL's tyrosine kinase binding domain (TKBD). CBLock disrupts the interaction between CBL mutants and RTKs, thereby impairing RTK-mediated priming of adaptor function of CBL mutants and downstream signaling. Notably, CBLock binds TKBD without inducing conformational changes, thereby preserving its ligand-free native conformation. In contrast, when CBL binds RTK substrates, TKBD undergoes a conformational change. Maintaining the native CBL TKBD conformation was crucial for CBLock to inhibit proliferation, induce cell-cycle arrest, and promote apoptosis in leukemia cells harboring *CBL* mutations. In a mouse xenograft model of acute myeloid leukemia (AML), CBLock reduced tumor burden and improved survival rate. Moreover, CBLock inhibited the proliferation of cells derived from patients with *CBL* mutations. Therefore, inhibiting CBL TKBD in its native state presents a promising therapeutic opportunity in targeting mutant CBL-dependent leukemia.

INTRODUCTION

In mammals, Casitas B-lineage Lymphoma (c-CBL or CBL) is one of the three members of CBL family proteins that also includes CBL-B and CBL-C. The highly conserved TKBD of CBL binds to and regulates the ubiquitination of activated non-receptor tyrosine kinases (TKs) and RTKs.[1–4] CBL-mediated negative regulation of activated RTKs is critical for inhibiting the sustained and aberrant TK signaling.[5] The TKBD in CBL is followed by the linker-helix region (LHR) and RING domain that together impart the E3 ligase function by recruiting a ubiquitin-conjugating enzyme thioesterified with ubiquitin (E2∼Ub) to facilitate ubiquitin (Ub) transfer.[6] Structural studies reveal that phosphorylation of LHR Tyr371 induces conformational changes that facilitate juxtaposition of bound E2∼Ub and the substrate and priming of a reactive E2∼Ub conformation for catalysis.[7,8] Under physiological settings, CBL gets recruited to an activated RTK upon growth factor stimulation causing extensive phosphorylation of CBL's tyrosine residues including Tyr371, thereby activating its E3 activity to attenuate RTK signaling.[2–4,7,9,10]

Almost 5% of different types of myeloid malignancies show *CBL* mutations.[11] The highest frequencies of *CBL* mutations are found in myelodysplastic syndrome/myeloproliferative neoplasms (MDS/MPN) such as juvenile myelomonocytic leukemia (JMML, ∼15%) and chronic myelomonocytic leukemia (CMML, ∼10%).[11,12] These patients generally have poor prognosis, with a low survival rate.[13,14] Currently, there is no curative treatment option available for them.[15] The majority of these mutations are missense mutations that cluster within the regions encoding the LHR and RING domains and disrupt CBL's E3 activity.[11] Tyr371 is a common site for most missense mutations and has the highest frequency in MDS/MPN patients.[11,16,17] In various cell lines and mouse models, CBL mutants induce oncogenic properties including CMML-like and other leukemic phenotypes.[18–24] CBL mutants can act as dominant negative molecules or can attain gain-of function (GOF) properties by

Received 13 September 2024; accepted 30 April 2025;
https://doi.org/10.1016/j.ymthe.2025.04.042.

[7]Present address: Q[2] solutions, Rosebank, Livingston EH54 7EG, UK

[8]Present address: Leicester Institute of Structural and Chemical Biology, Department of Molecular and Cell Biology, University of Leicester, Leicester LE1 7RH, UK

Correspondence: Danny T. Huang, Cancer Research UK Scotland Institute, Garscube Estate, Switchback Road, Glasgow G61 1BD, UK.
E-mail: d.huang@crukscotlandinstitute.ac.uk

 © 2025 The Authors.
Published by Elsevier Inc. on behalf of The American Society of Gene and Cell Therapy.
This is an open access article under the CC BY license (http://creativecommons.org/licenses/by/4.0/).

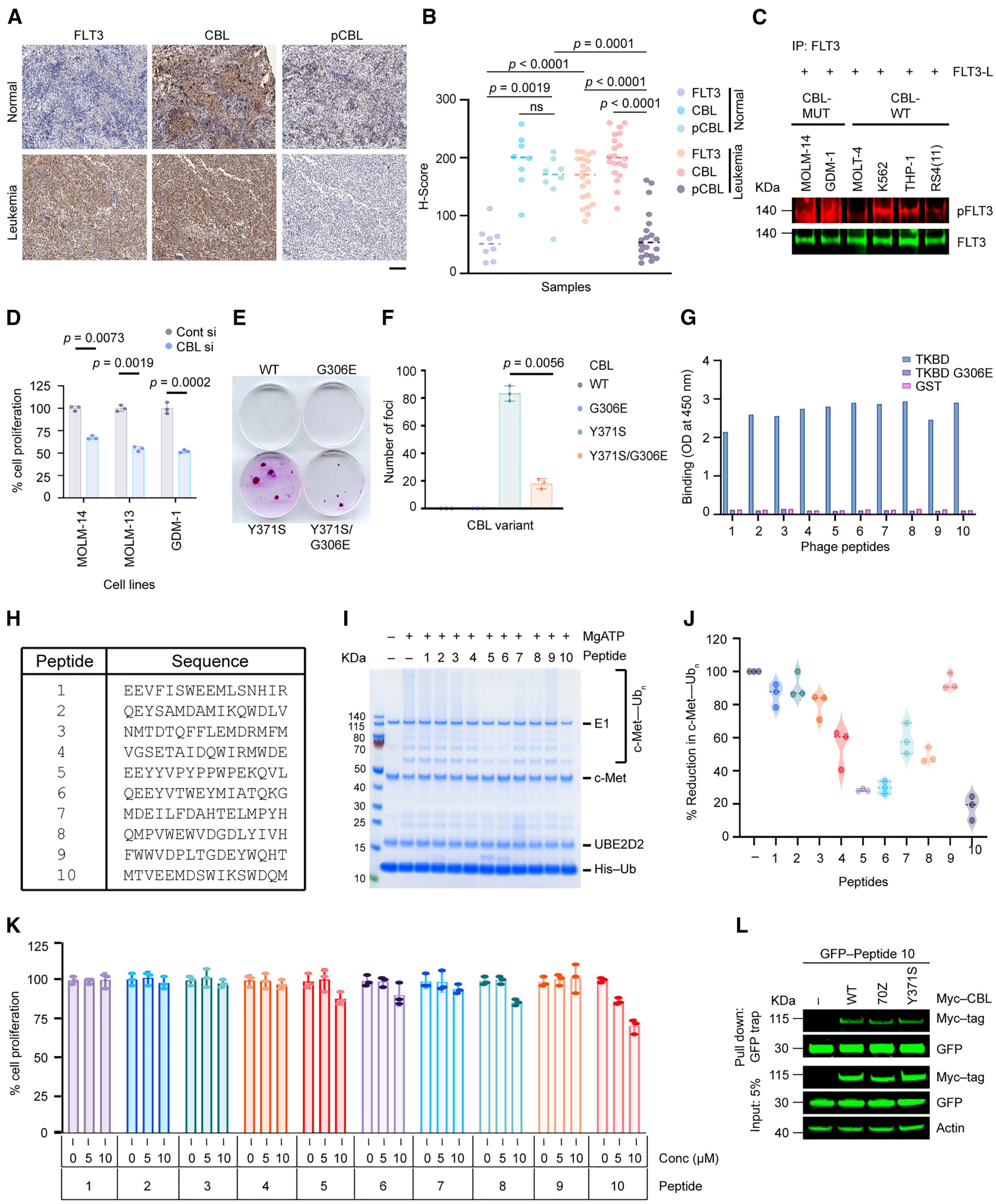

Figure 1. Deregulated mutant CBL activity can be targeted in leukemia

(A) Representative examples of immunohistochemical staining of FLT3, CBL, CBL pY371 (pCBL) in normal (n = 8) and leukemia (n = 20) samples, description provided in the materials and methods section. Images were exported using Halo software at ×20 original magnification. The scale bar (bottom right) represents 100 μm in each panel. (B) Quantification of FLT3, CBL, and pCBL expression as shown by the respective histoscore (H-Score) for the normal and leukemia cores of the multi-organ tissue microarray in (A). (C) Co-immunoprecipitation assay of CBL mutant (MOLM-14, GDM-1) and CBL-WT [MOLT-4, K562, THP-1 and RS4(11)] from cell lysates treated with 10 ng/mL FLT-L for 3 h. Anti-FLT3 antibody immunoprecipitants and cell lysates were analyzed by immunoblotting with anti-pFLT3 and anti-FLT3 antibodies as indicated. (D) Proliferation of *CBL* mutant-expressing leukemia cells knocked down with control siRNA (cont si) or CBL siRNA (CBL si) and analyzed after 48 h using Cell Titer-Glo and represented as percentage of cells knocked down with cont si. (E) Foci-formation assay using NIH3T3cells stably expressing indicated CBL variants. (F) Quantification of (E) (n = 3 independent experiments). (G) ELISAs for phage-displayed peptides binding to GST-CBL variants and GST control. Absorbance at 450 nm (y axis) was plotted against phage peptides (x axis). The experiment was repeated three times (n = 3 repeated experiments). (H) Sequences of phage-displayed peptides identified in (G). (I) Coomassie-stained

(legend continued on next page)

acting as adaptor molecules.[20,22] Under different pathophysiological settings, they activate signaling pathways including AKT/PI3K, ERK, and STATs to promote oncogenesis.[20,23–36]

Here, we explore the feasibility of targeting the TKBD substrate-binding site to inhibit the oncogenic properties of CBL mutants found in myeloproliferative diseases. Using a phage-displayed peptide library and structure-based optimization, we identified a peptide CBLock that binds the CBL TKBD substrate-binding site. In addition to disrupting the interaction between CBL mutants and RTK, CBLock also locks the TKBD in its native conformation. This dual effect is crucial for CBLock in preventing the priming of the adaptor function of CBL mutants and hindering their GOF, resulting in the inhibition of the proliferation in cell lines harboring *CBL* mutations. Conditional expression of CBLock in a xenograft mouse model of AML resulted in reduced tumor progression and improved survival. Furthermore, EBV immortalized B cells of patients with *CBL* mutations showed reduced proliferation upon CBLock treatment. These findings provide a rationale for targeting the TKBD substrate-binding site in CBL mutant-dependent cancers for therapeutic intervention.

RESULTS

Deregulation of CBL activation in different leukemias

Phosphorylation of Tyr371 is critical for activating CBL's E3 activity to downregulate RTK signaling.[7] Overexpression of RTKs such as FLT3 is a characteristic feature observed in different types of leukemias as was observed in a multi-organ human cancer tissue microarray (Figure 1A). While the mutational status of *CBL* in these leukemic cancer patient samples is unknown, we observed that, despite exhibiting high expression levels of CBL protein in the cancer samples, CBL predominantly remained in the Tyr371-unphosphorylated state compared with the normal samples (Figures 1A and 1B). This highlights that deregulation in the activation of CBL's E3 activity is a common feature in different leukemias.

MOLM-14, MOLM-13, and GDM-1 are human monocytic leukemia cell lines that harbor *CBL* mutations. MOLM-14 and MOLM-13 are heterozygous for a deletion in exon 8 of *CBL* along with internal tandem duplication (ITD) of *FLT3*.[17,37,38] The deletion in exon 8 results in a CBL mutant lacking residues 366–409, similar to CBL-70Z, which lacks the LHR and a portion of the RING domain, thereby disrupting E3 activity.[39] GDM-1 harbors a homozygous R420Q mutation in *CBL*.[38] R420 is the critical linchpin residue that primes the active closed E2∼Ub conformation for catalysis.[8] Indeed, R420Q substitution greatly reduced the E3 activity of CBL (Figure S1A). Upon addition of FLT3 ligand, the *CBL* mutant cell lines displayed elevated pFLT3 levels compared with cell lines that harbored CBL-WT (Figure 1C). In addition, knockdown of *CBL* mutants in these cell lines caused growth inhibition (Figures 1D and S1B), in line with prior findings demonstrating the oncogenic properties of CBL mutants.[17] However, we did not observe a similar effect of CBL knockdown in leukemia cells expressing CBL-WT (Figures S1C and S1D). These results suggest that CBL mutants provide growth advantage to the CBL mutant harboring cell lines, consistent with prior findings.[18–24]

Since most CBL mutations occur in the LHR and RING domains, the TKBD remains intact, allowing it to interact with RTK.[28] RTK binding is an important trigger for the CBL mutants to gain oncogenic properties. We previously showed that stable overexpression of CBL-Y371S in NIH3T3 cells enhanced cell transforming efficiency.[28,39] Here, we incorporated a secondary site mutation G306E in CBL-Y371S that disrupts RTK-CBL TKBD interactions and found that the CBL-Y371S/G306E mutant had significant reduction in foci-forming ability as compared with CBL-Y371S (Figures 1E and 1F). These findings suggest that disrupting RTK-CBL interactions might be a potential approach to inhibit oncogenic properties of CBL mutants.

Screening for a CBL TKBD binding peptide

CBL's TKBD is known to bind phosphotyrosine peptides from RTKs.[1] To identify peptides that bind selectively to CBL's TKBD substrate-binding site, we used a phage-displayed library of random 16-residue peptides to perform binding selections with CBL TKBD and its G306E counterpart as a negative control. Several peptide sequences were obtained (Figures 1G and 1H). We confirmed the binding of the top 10 peptides to TKBD by surface plasmon resonance (SPR) and observed that peptide10 (MTVEEMDSWIKSWDQM) showed the highest affinity for TKBD with a K_D of ∼400 nM (Table 1; Figure S2). Further, in an *in vitro* ubiquitination assay using c-Met as a substrate, we found that peptide10 exerted the highest inhibitory effect on CBL activity (Figures 1I and 1J). We treated MOLM-14 cells with these 10 peptides in the presence of Antennapedia, a cell-penetrating peptide, and found that peptide10 reduced cell proliferation with the highest efficiency (Figure 1K). Thus, we focused further investigation on peptide10.

We expressed a GFP-peptide10 fusion protein in HEK293 cells and observed that GFP-peptide10 could be immunoprecipitated with WT and CBL mutants with similar efficiency (Figure 1L). Mass spectrometry analysis of GFP-peptide10 pulldown products revealed that CBL and CBL-B are the major interacting partners (Figure S3).

gel showing *in vitro* ubiquitination assay performed with purified pCBL and c-Met in absence or presence of 20 μM peptide (1–10). (J) Quantification of c-Met ubiquitinated bands in (I) (*n* = 3 independent experiments). (K) Proliferation of MOLM-14 cells treated with vehicle (DMSO) or different concentrations of peptide (1–10) along with 1 μM Antennapedia peptide were analyzed after 72 h using Cell Titer-Glo and represented as percentage of cells treated with vehicle. (L) Co-immunoprecipitation assay of HEK293 cell lysates overexpressing GFP-Peptide10 fusion protein along with empty vector (−) or Myc-tagged CBL variants. Anti-GFP trap pulled down immunoprecipitants and cell lysates were analyzed by immunoblotting with anti-Myc–tag, anti-GFP and anti-Actin antibodies as indicated, where the Actin panel is a sample control. The data points in (D), (F), (J), and (K) are presented from three independent experiments while the error bars in (D), (F), and (K) represent ±SD (*n* = 3 independent experiments).

Table 1. Equilibrium dissociation constant, K_D, values for interactions between immobilized GST-CBL TKBD residues 47–355 variants and peptide or Ub-peptide

Analyte	K_D (μM)
Peptide1	N.M.
Peptide2	N.M.
Peptide3	N.M.
Peptide4	27.0 ± 1.2
Peptide5	39.2 ± 1.2
Peptide6	20.5 ± 0.4
Peptide7	20.8 ± 0.5
Peptide8	4.4 ± 0.3
Peptide9	N.M.
Peptide10	0.40 ± 0.05
Ub-peptide10	0.49 ± 0.07
GFP-peptide10	1.4 ± 0.1
GFP	N.B.
Ub-peptide10 D7A	N.M.
Ub-peptide10 W9A	27.7 ± 0.1
Ub-peptide10 I10A	N.M.
Ub-peptide10 D14A	N.M.
Ub-P37A/Q40A–peptide10	0.86 ± 0.03
Ub-peptide10 D7N	26.7 ± 0.3
Ub-peptide10 K11R	0.59 ± 0.01
Ub-peptide10 M16R (Ub-CBLock)	0.27 ± 0.02
Ub-peptide10 17R	0.57 ± 0.04
CBLock	0.075 ± 0.008

SEM is indicated for n = 2 technical replicates. N.M. = not measurable; it was not possible to estimate the K_D in the analyte concentration range. N.B. = no binding. Representative sensorgrams and binding curves are shown in Figure S2.

Together, these results show that peptide10 binds to the CBL TKBD with high affinity and specificity.

Structural insights into the interaction between CBL TKBD and peptide10

To gain insight into their binding mode, we tried to crystallize the complex between CBL TKBD and peptide10. To improve its solubility, we fused peptide10 to the C terminus of Ub or GFP to generate Ub-peptide10 and GFP-peptide10, respectively. Ub-peptide10 and GFP-peptide10 displayed K_D values of 0.49 and 1.4 μM, respectively, for the CBL TKBD (Table 1; Figure S2). While these tags had some effects on binding, they improved the solubility of peptide10 necessary for crystallization and further characterization. We successfully crystallized and determined the structure of TKBD bound to Ub-peptide10 at 2.02 Å resolution (Figure 2A; Table S1). The structure shows that peptide10 occupies the TKBD substrate-binding site and the binding mode overlaps with interactions between the TKBD and phosphotyrosine peptides from substrate RTKs (Figure 2B). However, in contrast to phosphotyrosine peptides from RTKs, peptide10 adopts an α-helical structure.

Peptide10 lacks the phosphotyrosine found in RTK substrates and the binding is predominantly stabilized by hydrophobic interactions and hydrogen bonds (Figure 2C). Ile10* and Trp13* in peptide10 (asterisks indicate peptide10 residues throughout) replace the phosphotyrosine in the TKBD substrate-binding pocket and, together with Met6* and Trp9*, form hydrophobic contacts to TKBD's Ala304 and Ile318. Hydrogen bonds occur between Asp7* and TKBD's Tyr274 and the backbone amide of Gln316, and between Asp14* and the backbone amide of Tyr274. Additional side chain stacking interactions are observed between Lys11* and Tyr274 and between Trp13* and Arg299. To verify the importance of the observed peptide10-TKBD interactions, we generated selected Ub-peptide10 mutants and analyzed their affinities for the TKBD. All mutants displayed reduced affinity for the TKBD with the I10A* variant showing no measurable binding (Table 1; Figure S2). Moreover, the same mutants expressed as GFP-peptide10 in cells also showed no interaction with Myc-tagged full-length WT-CBL (Figure 2D). The mutant peptide10-I10A was also defective in inhibiting CBL-mediated c-Met ubiquitination *in vitro* (Figure 2E). Ub makes very few interactions with the TKBD that primarily involve Pro37 and Gln40 in Ub contacting an adjacent surface of the TKBD substrate-binding site (Figure S4A). The substitution of both Ub residues to alanine had a minimal effect on the affinity between Ub-peptide10 and the TKBD (Table 1; Figure S2).

Closer inspection of the structure suggests that there are a few possible ways to improve the binding affinity of peptide10. The C-terminal Met16* is within 5–10 Å of Asp86 and Asp90 of TKBD (Figure S4B). We considered substituting Met16* to Arg and adding an additional Arg at the C terminus to facilitate interaction with the sidechains of Asp86 and Asp90. We also explored D7N* and K11R* substitutions to enhance the interaction. We generated these variants in Ub-peptide10 and examined their binding affinity to the CBL TKBD. We found that the D7N* substitution disrupted binding, whereas the K11R* substitution and addition of Arg17* had little effect (Table 1; Figure S2). Interestingly, the M16R* substitution not only improved affinity but also enhanced solubility in aqueous solution. Consequently, we focused our study on peptide10-M16R, referring it to as CBLock from hereon. CBLock displayed a K_D value of 75 nM for the CBL TKBD (Table 1; Figure S2).

CBLock binds CBL in its native conformation and inhibits cell proliferation

All the published structures of ligand-free CBL containing TKBD alone or TKBD-LHR-RING domain show that the TKBD adopts a similar conformation, which we will refer to as the native conformation from hereon. The binding of phosphotyrosine substrate peptides induces a conformational change in the TKBD. However, binding of peptide10 did not induce a conformational change in the TKBD; therefore, peptide10 binds TKBD in its native ligand-free conformation (Figures 3A and 3B).

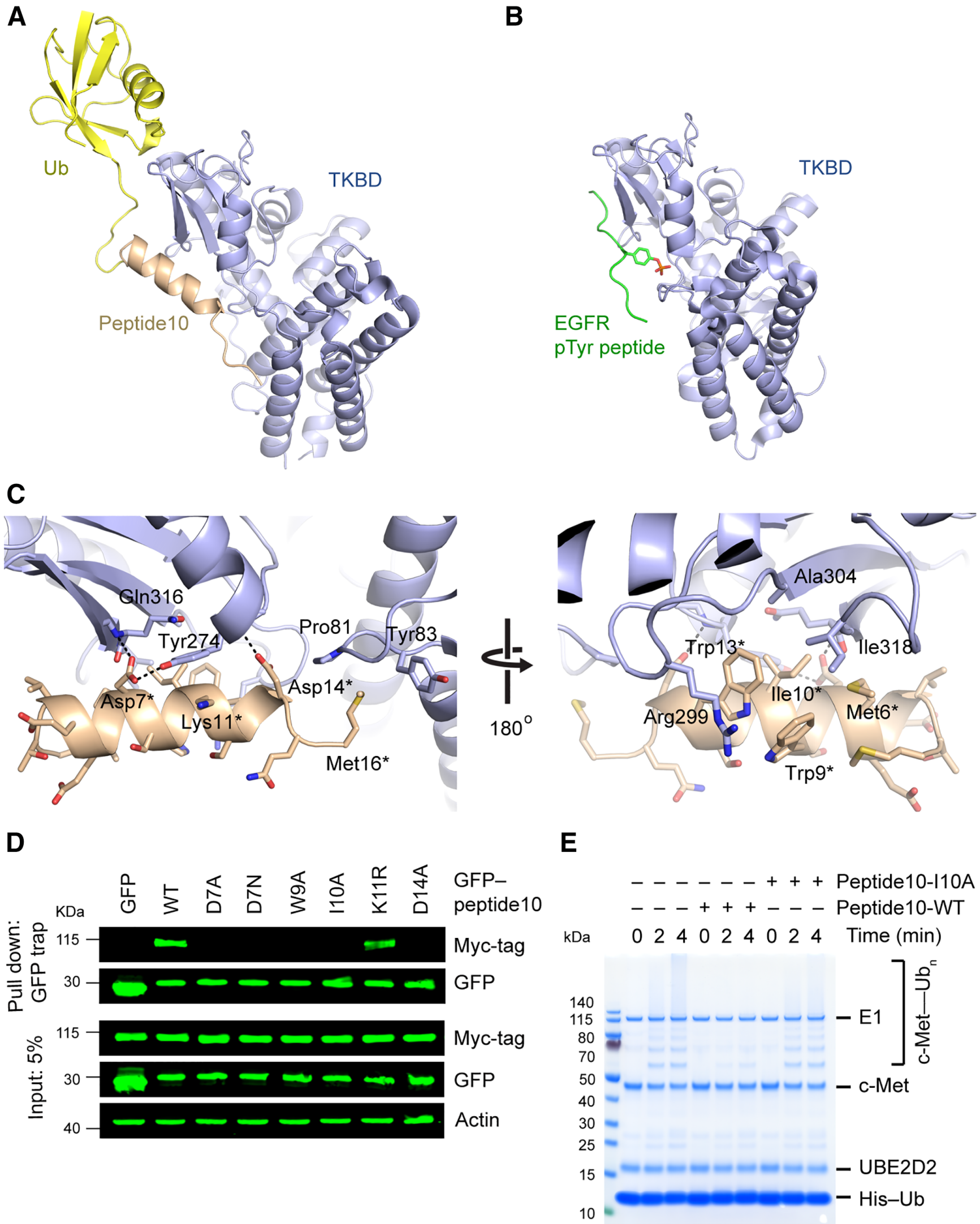

Figure 2. Peptide10 binds CBL TKBD substrate-binding site and competes against RTK binding
(A) Cartoon representation of the structure of CBL TKBD bound to Ub-peptide10: TKBD in blue, Ub and linker in yellow, and peptide10 in wheat. (B) Cartoon representation of the structure of CBL TKBD bound to EGFR phosphotyrosine (pTyr) peptide (PDB: 3BUO) in the same orientation as (A): TKBD in blue, EGFR pTyr peptide in green, pTyr in stick with red phosphate. (C) Two close-up views of CBL TKBD and peptide10 binding interface rotated by 180°. Key interacting residues are shown as sticks and colored as in (A). O and N atoms are colored red and blue, respectively. Dashed black lines depict hydrogen bonds. Asterisks (*) indicate peptide10 residues. (D) Co-immunoprecipitation assay of HEK293 cell lysates overexpressing GFP-tagged peptide10 and variants, or GFP alone along with Myc-tagged CBL-WT. Anti-GFP trap pulled down immunoprecipitants and cell lysates were analyzed by immunoblotting with anti-Myc-tag, anti-GFP, and anti-Actin antibodies as indicated, where the Actin panel is a sample control. (E) Coomassie-stained gel showing *in vitro* ubiquitination assay performed with pCBL and c-Met in the absence or presence of 10 μM peptide10 or peptide10-I10A.

To assess the differences in TKBD conformation upon binding of CBLock or phosphotyrosine substrate peptides, we performed a disulfide assay as described previously.[7] We engineered the CBL (47–435) R139C/D435C variant (diCys; consisting of TKBD-LHR-RING domain), where R139C and D435C are in close proximity and readily form a disulfide bond when CBL is in the ligand-free state. The distance between R139C and D435C becomes greater upon binding of phosphotyrosine substrate peptide (Figure 3C). The unmodified diCys migrated slower than the disulfide-bonded diCys on SDS-PAGE (Figures 3D and 3E), and we assessed the rate of disulfide bond formation of diCys in the presence of CBLock. CBL-WT did not form disulfide bonds. Addition of phosphotyrosine substrate peptide from ZAP70 (pZAP70) or SYK (pSYK) prevented formation of the diCys disulfide bond (Figures 3D and 3E), consistent with an increase in the distance between R139C and D435C in CBL-substrate complexes. The addition of CBLock did not affect diCys disulfide bond formation (Figures 3D and 3E), suggesting that it binds CBL TKBD in the native conformation.

To determine if the difference in the CBL conformation upon binding of TKBD by CBLock or phosphotyrosine substrate peptides influences CBL stability, we performed a thermal shift assay using cell-penetrating CBLock and pSYK peptide. A previous study fused the *Drosophila Antennapedia* homeodomain cell-penetrating peptide to the N terminus of the pSYK peptide (hereafter referred to as cp-pSYK) to investigate the role of CBL-B in antifungal immunity and demonstrated its efficacy in protecting cell lines and mice from *C. albicans* infections.[40] We fused the Antennapedia cell-penetrating sequence to the N terminus of CBLock (referred to as cp-CBLock) (Figure S5A). We then assessed the cell-penetrating efficiency of cp-CBLock and observed that cp-CBLock could penetrate cells and was also stable for 48 h (Figures S5B and S5C). We found that addition of the cp-pSYK peptide resulted in reduced apparent melting temperature (T_m) of CBL. In contrast, cp-CBLock addition led to a slight stabilization of CBL (Figures 3F and 3G).

We also observed that cp-CBLock but not cp-pSYK peptide could inhibit the proliferation of MOLM-14 cells across a range of different concentrations, indicating the need to block mutant CBL TKBD in its native conformation to exert growth inhibitory properties (Figure 3H). Collectively, these results showed that CBLock binds CBL TKBD in its native conformation and that

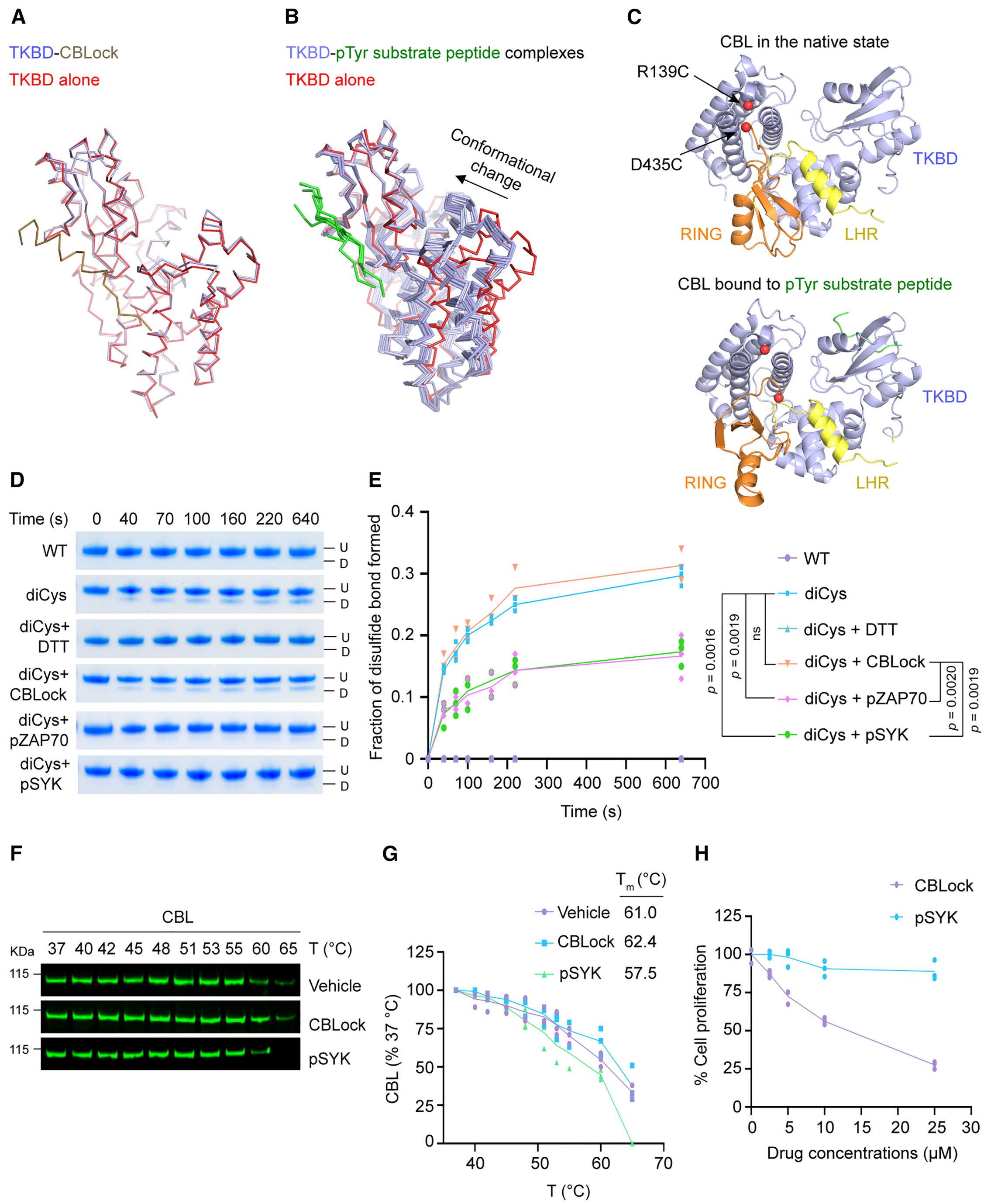

Figure 3. Peptide10 and CBLock bind CBL TKBD in the native conformation

(A) Ribbon overlay of TKBDs from TKBD-peptide10 portion of TKBD-Ub-peptide10 complex (colored as in Figure 2A) and TKBD alone (red; PDB: 1B47) (RSMD = 0.4 Å across 264 Cα atoms). (B) Ribbon overlay of CBL TKBDs (residues 247–350) from six CBL TKBD-phospho-tyrosine substrate peptide complexes (PDBs: 2CBL, 3BUO, 3BUX, 3BUW, 3BUM, 1YVH; colored as in Figure 2B) and TKBD alone (red; PDB: 1B47) (RSMD = 0.39–0.55 Å across 82 Cα atoms). TKBD conformational change upon binding phosphotyrosine substrate peptide is indicated. (C) Probing CBL conformational change with diCys. Cartoon representation of CBL structures in the native state (PDB: 2Y1M, top panel) and bound to pZAP70 peptide (PDB: 2Y1N, bottom panel): TKBD in blue, LHR in yellow, and RING domain in orange. R139C and D435C are indicated by red spheres. (D) Coomassie-stained SDS-PAGE gel showing disulfide bond formation for CBL 47–435 (WT) or diCys in the absence or presence of CBLock, pZAP70, or pSYK peptide. Unmodified (U) and disulfide-bonded (D) species are indicated. (E) Plots showing the fraction of disulfide bond formed over time for CBL variants in (D). The data points are presented from three independent experiments (n = 3 independent experiments). (F) For the thermal shift assay, HEK293 cells treated with vehicle

(legend continued on next page)

binding in this state may be crucial for limiting the oncogenic potential of CBL mutant.

CBLock restricts RTK-dependent GOF properties of mutant CBL

We found that small interfering RNA (siRNA)-mediated knockdown of *CBL* in leukemia cell lines that harbor CBL mutations caused growth inhibition (Figure 1D). We, therefore examined whether CBLock treatment could elicit growth inhibitory effects on leukemia cells harboring CBL mutants and found that MOLM-14, MOLM-13, and GDM-1 cells showed significantly reduced proliferation upon treatment with cp-CBLock (Figure 4A). We also found that treatment with cp-CBLock-WT but not cp-CBLock-I10A caused Sub-G1 cell-cycle arrest of MOLM-14 cells (Figures 4B and S6). In addition, CBLock treatment showed signs of apoptosis as cleaved caspase-3 and cleaved PARP were detectable in a dose-dependent manner in MOLM-14 cells but not in K562 cells (Figure 4C). These data were corroborated by a foci-forming assay where we observed NIH3T3cells stably expressing CBLock-WT but not CBLock-I10A could inhibit the foci-forming ability of CBL-Y371S (Figures 4D and 4E). Together, these data showed that CBL mutant-dependent cancer cell growth can be inhibited by targeting the TKBD substrate-binding site of CBL.

In an immunoprecipitation experiment, we showed that CBLock-WT but not CBLock-I10A prevented the interaction between EGFR and CBL-Y371S (Figure 4F). Moreover, CBLock-WT reduced the elevated CBL-pY731 levels in HEK293 cells expressing CBL mutants, whereas CBLock-I10A did not exert this effect (Figure 4G). Hyper-phosphorylation of the C terminus of CBL mutant allows for additional interactions with adaptor proteins usually not found with CBL-WT. Treatment of MOLM-14 cells with cp-CBLock showed reduced ability of CBL mutant to interact with a number of key partners (Figure 4H). We validated some of the key interactors using immunoprecipitation (Figure S7A). Interestingly, knockdown of *MRG1*, *BUD31*, and *Ikaros* resulted in reduced proliferation of MOLM-14 cells (Figures 4I and S7B), suggesting that the ability of CBL mutant to interact with distinct partners could provide enhanced growth properties to these cells. Together, these data demonstrate the ability of CBLock to cause growth arrest and apoptosis in leukemia cells harboring *CBL* mutants by disrupting its RTK-mediated GOF properties.

CBLock inhibits disease progression in a murine model of AML

To study the *in vivo* antitumor effect of CBLock, a mouse model of AML was established by the tail vein injection of 5 million MOLM-$13^{Luc/CBLock}$ cells into NSG mice (Figures S8A–S8C). Doxycycline (dox) treatment, which induced the expression of CBLock, led to the inhibition of proliferation of these cells but not the parental MOLM-13 luciferase expressing, MOLM-$13^{parental}$ cells (Figures S8D–S8F). Alternatively, we also designed CBLock mutants, including CBLock-DM (D7A/I10A), CBLock-Sc1 (SMEKTDQWRMVEIDSW), and CBLock-Sc2 (IKDWQDRSVWESMTME), which failed to bind the CBL mutant 70Z (Figure S8G). The corresponding MOLM-13 cell lines, MOLM-$13^{Luc/CBLock-DM}$, MOLM-$13^{Luc/CBLock-Sc1}$, and MOLM-$13^{Luc/CBLock-Sc2}$, efficiently expressed the iRFP-CBLock variants, similar to the MOLM-$13^{Luc/CBLock}$ cells (Figure S8H). We estimated the concentrations of CBLock-WT, CBLock-DM, CBLock-Sc1, and CBLock-Sc2 after dox treatment on different days using a purified iRFP-CBLock as standard and observed that the concentration of iRFP-CBLock variants ranged from approximately 1.4 to 7.9 μM (Figures S8I and S8J). Although the concentrations of all the expressed iRFP-CBLock variants were at comparable levels, only CBLock-WT inhibited the proliferation of the MOLM-13^{Luc} cells, while the other variants did not, further confirming the specificity of CBLock (Figure S8K). Therefore, we decided to inject MOLM-$13^{Luc/CBLock}$ cells into the NSG mice. Following cell injection, bioluminescence imaging showed that all the mice efficiently took up the cells (Figure S9A). The mice were then divided into two groups of eight and received either water (vehicle) or doxycycline (dox, 100 mg/kg) daily through oral gavage. On day 12, the vehicle-treated mice reached clinical endpoint and were euthanized. The dox (CBLock) -treated mice were continuously monitored and were euthanized before reaching clinical endpoint on day 21 to assess the differences in oncology parameters and physiological indicators (Figure 5A). Dox-induced CBLock expression significantly delayed tumor growth and progression compared with the vehicle-treated mice as per the bioluminescence intensity using IVIS system (Figures 5B, 5C, S9B, and S9C). On successive days (3, 7, and 10) of imaging, the vehicle group showed significantly higher total flux (p/s) than the dox group. Tumor-bearing mice receiving dox also showed prolonged survival, as shown in Kaplan-Meier curves (Figure 5D).

To evaluate the severity of disease progression in the mice, different organs (spleen, legs, lymph nodes, spine, ribs, kidney, liver, lungs) were harvested from the two groups of mice. *Ex vivo* bioluminescence imaging of the vehicle group mice organs showed significantly higher total flux as compared with the dox group mice organs (Figures 5E and 5F), suggesting rapid infiltration of the myeloid blasts into several of these organs that could be slowed down by dox treatment. The bioluminescence signal emanating from the brain and cranium region when assessed and quantified also showed a similar trend (Figure 5F). Dox-treated mice also showed absence of splenomegaly, further confirming the reduced tumor burden in the dox group mice (Figure 5G). H&E staining of the organs (spleen, legs, spine, ribs) from the two groups of mice showed strong infiltration of MOLM-$13^{Luc/CBLock}$ cells in the vehicle mice and only mild infiltration in the dox-treated mice

(water), 10 μM cp-CBLock or cp-pSYK peptide were lysed and the stability of CBL was checked through immunoblotting. (G) Plots showing the relative stability of CBL in presence of different treatments in (F). Band intensities were normalized to the band at 37°C for each condition. The data points are presented from three independent experiments (n = 3 independent experiments). (H) Proliferation of MOLM-14 cells treated with cp-CBLock or cp-pSYK peptides over a range of concentrations was analyzed after 72 h using Cell Titer-Glo and represented as percentage of cells treated with peptide concentration at 0 μM (vehicle). The data points are presented from three independent experiments (n = 3 independent experiments).

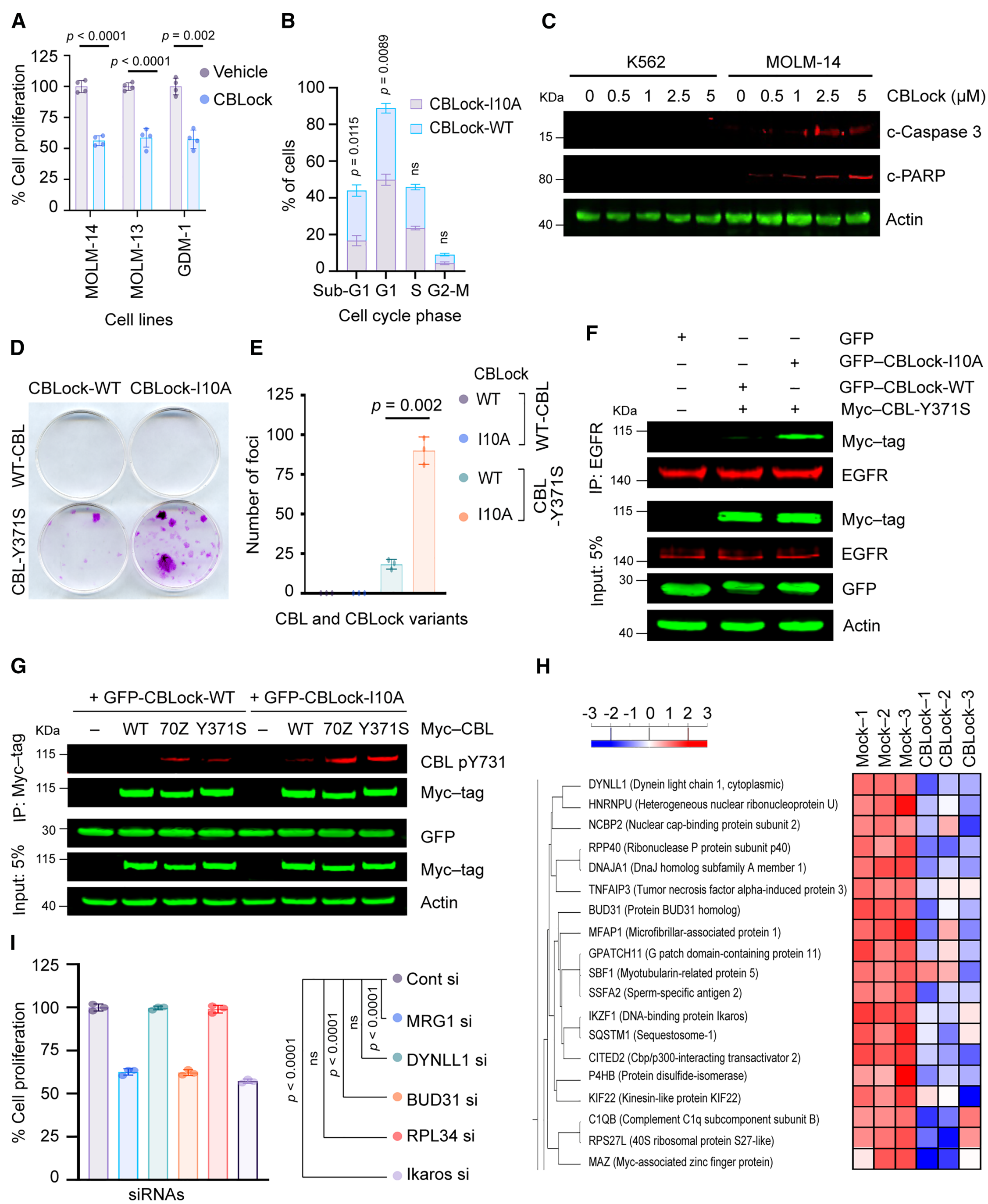

Figure 4. CBLock inhibits RTK-mediated GOF properties in CBL mutants

(A) Proliferation of *CBL* mutant-expressing cells treated with vehicle (water) or 5 μM cp-CBLock peptide were analyzed after 72 h using Cell Titer-Glo and represented as percentage of cells treated with vehicle. The data points are presented from three independent experiments while the error bars represent ±SD (n = 3 independent experiments). (B) Percentage of cells in different phases of cell cycle are represented. MOLM-14 cells were treated with 5 μM cp-CBLock-WT or cp-CBLock-I10A for 72 h, followed by PI staining and cell-cycle profile analysis by FACS. The data are presented as mean of three independent experiments while the error bars represent ±SD (n = 3 independent experiments). (C) Expression of c-Caspase 3, c-PARP, and Actin (loading control) from lysates of K562 and MOLM-14 cells treated with different concentrations of cp-CBLock. (D) Foci-formation assay using NIH3T3cells stably expressing CBL-WT or CBL-Y371S in the presence of either GFP-CBLock-WT or GFP-CBLock-I10A. (E) Quantification of (D), data are presented from three independent experiments while error bars represent ±SD (n = 3 independent experiments). (F) Co-immunoprecipitation assay of HEK293 cell lysates overexpressing Myc-tagged CBL-Y371S or the EV (–) along with GFP, GFP-CBLock-WT, or GFP-CBLock-I10A as indicated in the figure.

(legend continued on next page)

(Figure 5H). There was no obvious difference in body weight between the two groups of mice (Figure S9D). Together, these data underscore the significance of CBLock-mediated CBL mutant inhibition in delaying tumor progression and prolonging survival in a CBL mutant-dependent mouse model of AML.

CBLock improves AML-like features by restraining RTK signaling

We further evaluated various physiological parameters to assess the efficacy of CBLock in our AML mouse model. We collected cardiac whole blood (WB) from both vehicle and dox group mice during harvest. Giemsa staining of blood smears showed a significantly higher number of myeloblasts in the WB of vehicle mice as compared with that of dox (CBLock)-treated mice (Figures 6A and 6B). We also performed complete blood count analyses of the vehicle- and dox-treated mice using ProCyte Dx Hematology Analyzer (IDEXX). The reduced counts of RBCs, reticulocytes (RET), platelets (PLT), and percent of hematocrits (HCT %) in AML mice could be significantly rescued by dox treatment to near normal reference intervals reported previously for NSG mice[41] (Figure 6C). Similarly, the lower neutrophil (NEUT %) and higher monocyte (MONO %) percentages in the AML mice were significantly improved upon dox treatment (Figure 6C). However, we did not observe significant differences in any other blood parameters between the two groups of mice (Figure S9E). Overall, dox-induced CBLock expression improved the changes in blood parameters in AML mice. In addition, we performed immunohistochemistry against human specific-CD33 and pERK1/2 in spleen and leg sections from the two groups of mice. There was stronger signal for both CD33 and pERK1/2 in the vehicle-treated mice tissue sections as compared to the dox-treated tissue sections (Figure 6D). Stronger positive signal for CD33 further confirms the higher MOLM-13$^{Luc/CBLock}$ cell infiltration in the vehicle-treated mice organs. Also, CBLock expression interfered with RTK signaling as was suggestive of the reduced pERK1/2 levels. In a separate experiment, we injected 1 million MOLM-13$^{Luc/CBLock}$ cells into the tail vein of 16 NSG mice and allowed the cells to establish and develop tumors before starting the dox treatment regime. Again, the mice were checked for cell uptake and it was observed that all the mice uniformly received the cells (Figure S9F). Disease progression was monitored by looking at the bioluminescence intensity using the IVIS system (Figure S9G). Once the animals reached a total flux (p/s) of $\geq 10^5$ on day 6, they were randomly divided into two groups (n = 8) based on the bioluminescence intensity and received either vehicle (distilled water) or dox daily through oral gavage starting on day 9 (Figure 6E). The vehicle-treated mice reached clinical endpoints on days 13 and 14 while the dox-treated mice reached clinical endpoints on days 21 and 22. Again, dox as compared with vehicle treatment significantly delayed tumor progression and improved survival of the AML mice (Figures 6F–6H), confirming the ability of CBLock to inhibit disease progression in these tumor-bearing animals. Moreover, no marked changes in body weights of the animals were observed (Figure S9H).

Furthermore, we treated Epstein-Barr virus immortalized B (EBV-B) cells derived from patients harboring the germline heterozygous CBL-Y371C variant and a somatic loss-of-heterozygosity UPD, who exhibited features of leukemic predisposition, with cp-CBLock. The treatment showed reduced proliferation of the EBV-B cells in a dose-dependent manner (Figure 7A). Similarly, knocking down *CBL* in those EBV-B cells resulted in reduced proliferation, suggesting that CBLock-mediated inhibition in these cells occurred through targeting of mutant CBL (Figure 7B). In addition, upon CBLock treatment, the EBV-B cells exhibited reduced levels of the downstream RTK effector targets, activated STAT5 (pSTAT5), ERK1/2 (pERK1/2), and AKT (pAKT) (Figure 7C). Collectively, these data demonstrate that CBLock can improve AML-like features by reducing downstream RTK signaling.

DISCUSSION

In this study, we examined whether inhibiting the upstream event of the CBL mutant's interaction with RTKs could restrict oncogenic properties of CBL mutant. Since CBL mutations mainly occur in the LHR and RING domains, the TKBD substrate-binding remains unaffected and retains the ability to interact with RTKs. We discovered CBLock that binds the TKBD substrate-binding site and blocks CBL mutant's interaction with RTKs. Unlike RTK substrates, CBLock binds TKBD without inducing a conformational change, thereby maintaining TKBD in the native conformation. This property was crucial for CBLock's ability to disrupt CBL mutant interactome that promotes oncogenic signaling. As a result, CBLock reduces cell proliferation, induces sub-G1 cell-cycle arrest, and triggers apoptosis in leukemia cells harboring *CBL* mutations. We validated CBLock's efficacy in inhibiting the oncogenic properties of CBL mutants using NIH3T3 cells overexpressing CBL Y371S mutant, leukemia cell lines harboring various *CBL* mutations, and EBV-B cells derived from pediatric patients carrying homozygous *CBL Y371C* mutation. Furthermore, we demonstrated the efficacy of CBLock in restraining the progression of leukemia in a xenograft mouse model. These findings

Anti-EGFR immunoprecipitants and cell lysates were analyzed by immunoblotting with anti-Myc–tag, anti-EGFR, anti-GFP, and anti-Actin antibodies as indicated. (G) Co-immunoprecipitation assay of HEK293 cell lysates overexpressing Myc-tagged CBL variants or the EV (−) along with either GFP-CBLock-WT or GFP-CBLock-I10A. Anti-Myc–tag antibody immunoprecipitants and cell lysates were analyzed by immunoblotting with CBL-pY731, anti-Myc–tag, anti-GFP, and anti-Actin antibodies as indicated. (H) Heatmap of mass spectrometry data comparing CBL interactomes in MOLM-14 cells in the presence of vehicle (water) or 5 μM cp-CBLock. MOLM-14 cells were treated with vehicle or cp-CBLock for 48 h and cell lysates were immunoprecipitated using anti-CBL antibody- or mouse IgG-conjugated agarose beads followed by mass spectrometric analysis. Red and blue represent strong- or no/weak-interaction, respectively. The experiment was performed in three biological replicates. (I) Proliferation of MOLM-14 cells transfected with control siRNA (cont si), MRG-1 (CITED2) siRNA (MRG-1 si), DYNLL1 siRNA (DYNLL1 si), BUD31 siRNA (BUD31 si), RPL34 siRNA (RPL34 si), or Ikaros siRNA (Ikaros si) and analyzed after 48 h using Cell Titer-Glo and represented as percentage of cells knocked down with cont si. Data are presented from three independent experiments while error bars represent ±SD (n = 3 independent experiments). The Actin panel is a sample control in (C), (F), and (G).

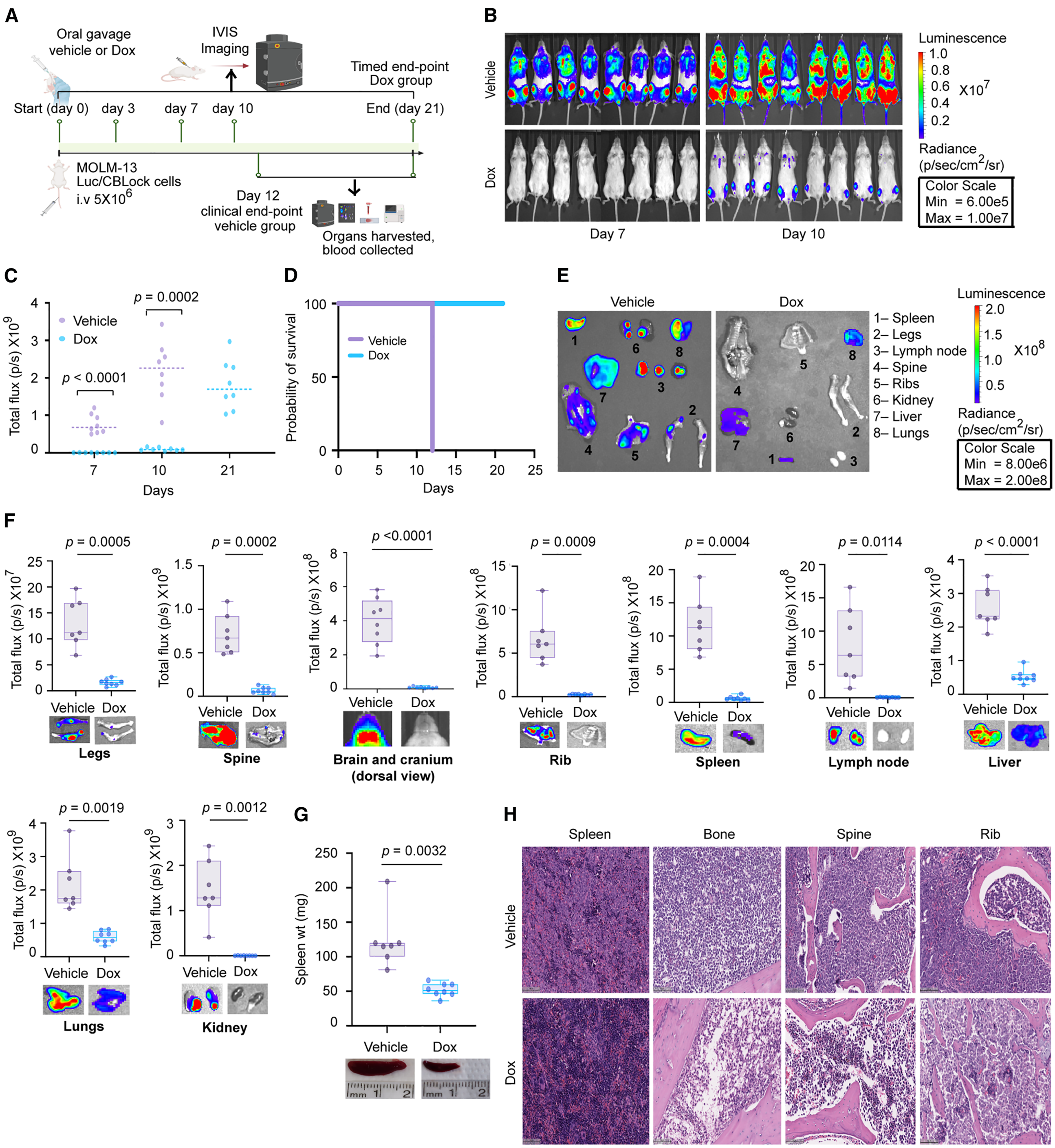

Figure 5. Induced expression of CBLock improves survival in a murine model of AML

(A) Schematic showing the experimental set up, injection of MOLM-13$^{Luc/CBLock}$ cells into NSG mice, bioluminescence imaging in the IVIS system, dox and vehicle treatment, euthanization of animals, and harvest of blood and organs. (B) Bioluminescence imaging of NSG mice intravenously injected with 100 μL of 5×10^6 MOLM$^{13Luc/CBLock}$ cells and treated with either vehicle (*n* = 8) or dox (*n* = 8) to regularly track disease progression. (C) Tumor burdens in both groups of mice in (B) were measured as total flux (p/s). Total flux (p/s) for the ventral view of the mice was calculated from the region of interest (ROI) of the bioluminescent images of the vehicle (*n* = 8) and dox- (*n* = 8) treated mice, the dashed lines represent median values. (D) Survival probability of the AML mice treated with vehicle or dox represented as Kaplan-Meier curves. All the vehicle-treated mice reached clinical endpoint on day 12, except one that was unable to recover from an imaging session on day 10, as a result this mouse was excluded from subsequent

(legend continued on next page)

elucidate the molecular mechanism of CBL mutant-dependent oncogenic processes in leukemia and point toward a mechanism by which CBLock exerts its inhibitory effect. CBLock blocks CBL mutants' interaction with RTKs, locks TKBD in its native conformation, and prevents subsequent RTK-mediated C-terminal tyrosine phosphorylation of CBL mutants, which is essential for their adaptor function. Consequently, CBL mutants are unable to recruit protein partners that drive cell growth and proliferation.

In recent years, several inhibitors targeting CBL-B have emerged. Among these are allosteric inhibitors that bind within the LHR pocket, keeping CBL-B in an inactive state,[42–44] and phosphotyrosine peptide inhibitors that target CBL-B's TKBD substrate-binding site.[40,45,46] These inhibitors likely also act on CBL due to identical binding site sequences. The allosteric inhibitors are unlikely to have a beneficial effect on CBL mutants as they already lack E3 activity and often have mutations in the LHR, which would prevent binding. The stability of phosphotyrosine in cells is questionable, but studies have shown efficacy of these phosphotyrosine peptide inhibitors in both cell lines and mouse models.[40,45] In contrast, CBLock lacks the phosphotyrosine yet binds CBL TKBD with nanomolar affinity. Notably, our structural and biochemical analyses showed that CBLock binds CBL TKBD in the native conformation, unlike phosphotyrosine substrate peptides such as the pSYK peptide. This distinction led us to explore whether CBLock's binding to CBL TKBD in its native conformation is a key aspect of its inhibitory effect on CBL mutants. We used the cp-pSYK peptide, previously shown to protect mice from *C. albicans* infection,[40] to assess its potential to inhibit the proliferation of MOLM-14 cells. Surprisingly, cp-CBLock, but not cp-pSYK, caused growth inhibition of MOLM-14 cells, suggesting that both disrupting RTK-CBL mutant interaction and locking CBL mutant's TKBD in the native conformation are crucial for restricting the oncogenic properties of CBL mutant. CBLock exhibits a K_D of 75 nM for CBL TKBD (Table 1), whereas pSYK peptide displayed a weaker K_D of 1.48 μM based on a prior report.[1] Despite observing no effect of cp-pSYK up to 25 μM (Figure 3H), we cannot rule out the possibility that the difference is attributable to variances in their binding affinities for CBL mutants. Nonetheless, by maintaining CBL TKBD's native conformation, CBLock limits unnecessary signaling that may arise from substrate peptide-induced conformational changes.

Previous studies have utilized orthotopic mouse models to demonstrate CBL mutant-dependent leukemic features *in vivo*.[47] We developed an orthotopic AML mouse model with MOLM-13 cells with inducible CBLock expression to evaluate the effect of CBLock *in vivo*. Induction of CBLock expression significantly reduced tumor progression and improved survival rate in the AML mouse model. CBLock reduced the infiltration of MOLM-13 cells in the spleen while reversing various AML-like blood parameters. These findings demonstrate the efficacy of CBLock in targeting CBL mutant-dependent oncogenic properties *in vivo*. Currently, leukemia patients with *CBL* mutations have limited treatment options. For JMML patients with *CBL* mutations, the recommendation often involves a wait-and-watch strategy.[13,48] Although FLT3 inhibitors can impede the CBL mutant-dependent leukemic features,[47] acquired resistance to these inhibitors commonly occurs after prolonged treatment.[49] Our study provides a rationale for targeting the TKBD substrate-binding site in CBL as a promising therapeutic opportunity for leukemia with *CBL* mutations. The discovery of CBLock in our work provides an initial platform for future optimization, such as using the stapled peptide approach to improve stability and cell delivery,[50] or developing small molecules that mimic its action.

MATERIALS AND METHODS

Plasmids

The constructs generated in this study were pBabe Puro FLAG-CBL-G306E, pBabe Puro FLAG-CBL-Y371S/G306E, pGEX4T1 GST-TEV CBL TKBD G306E (47–355), pGEX4T1 His-GST-Thrombin c-Met (995–1360), RSF_DUET His-TEV Ub-peptide10-6xHis, RSF_DUET His-TEV Ub-peptide10, RSF_DUET His-TEV Ub-peptide10-D7A, RSF_DUET His-TEV Ub-peptide10-D7N, RSF_DUET His-TEV Ub-peptide10-W9A, RSF_DUET His-TEV Ub-peptide10-I10A, RSF_DUET His-TEV Ub-peptide10-K11R, RSF_DUET His-TEV Ub-peptide10-D14A, RSF_DUET His-TEV Ub-peptide10-M16R, RSF_DUET His-TEV Ub-peptide10-17R, RSF_DUET His-TEV Ub-P37A/Q40A-peptide10, RSF_DUET His-TEV GFP-peptide10, pcDNA3.1 GFP-peptide10, pcDNA3.1 GFP-peptide10-D7A, pcDNA3.1 GFP-peptide10-D7N, pcDNA3.1 GFP-peptide10-W9A, pcDNA3.1 GFP-peptide10-I10A, pcDNA3.1 GFP-peptide10-K11R, pcDNA3.1 GFP-peptide10-D14A, pcDNA3.1 GFP-CBLock-D7A/I10A (GFP-CBLock-DM), pcDNA3.1 GFP-CBLock-scrambled1 (GFP-CBLock-Sc1), pcDNA3.1 GFP-CBLock-scrambled2 (GFP-CBLock-Sc2), pBabe *Neo* CBLock, pBabe *Neo* CBLock-I10A, pCW57 *Neo* iRFP- CBLock (iRFP-CBLock), pCW57 *Neo* iRFP- CBLock-DM (iRFP-CBLock-DM), pCW57 *Neo* iRFP-CBLock-Sc1 (iRFP-CBLock-Sc1), pCW57 *Neo* iRFP-CBLock-Sc2 (iRFP-CBLock-Sc2), pGEX4T1 His-GST-Thrombin CBL-WT (47–435) R420Q, and RSF_DUET His-TEV iRFP-CBLock (His-iRFP-CBLock).

analyses (n = 7), while the dox-treated mice were euthanized on day 21 at a timed endpoint (with no signs of clinical endpoint). (E) *Ex vivo* bioluminescence imaging of the organs harvested from AML mice treated with vehicle (n = 7) or dox (n = 8) to analyze the extent of disease progression. (F) Plots showing the total flux, calculated from the region of interest (ROI) of the bioluminescent images of organs of the vehicle or dox-treated mice. (G) Representative image of spleens from vehicle (n = 7) and dox- (n = 8) treated AML mice. Spleens of vehicle-treated mice suggestive of splenomegaly. Plot showing significant difference in spleen weights of vehicle- and dox-treated mice. (H) Representative images from H&E staining of FFPE tissue sections of different organs (spleen, legs, spine, rib) of vehicle- (n = 7) or dox- (n = 8) treated AML mice, showing MOLM-13$^{Luc/CBLock}$ cell infiltration. Images were exported using Halo software at ×20 original magnification. The scale bar (bottom left) represents 100 μm in each panel. Data points in (F) and (G) represent individual mice harvested at clinical endpoint (day 12) for vehicle (n = 7) and timed endpoint (day 21) for dox- (n = 8) treated mice, the error bars represent ±SD. The p values in (C), (F), and (G) were calculated using Student's t test (unpaired, parametric) with Welch's correction.

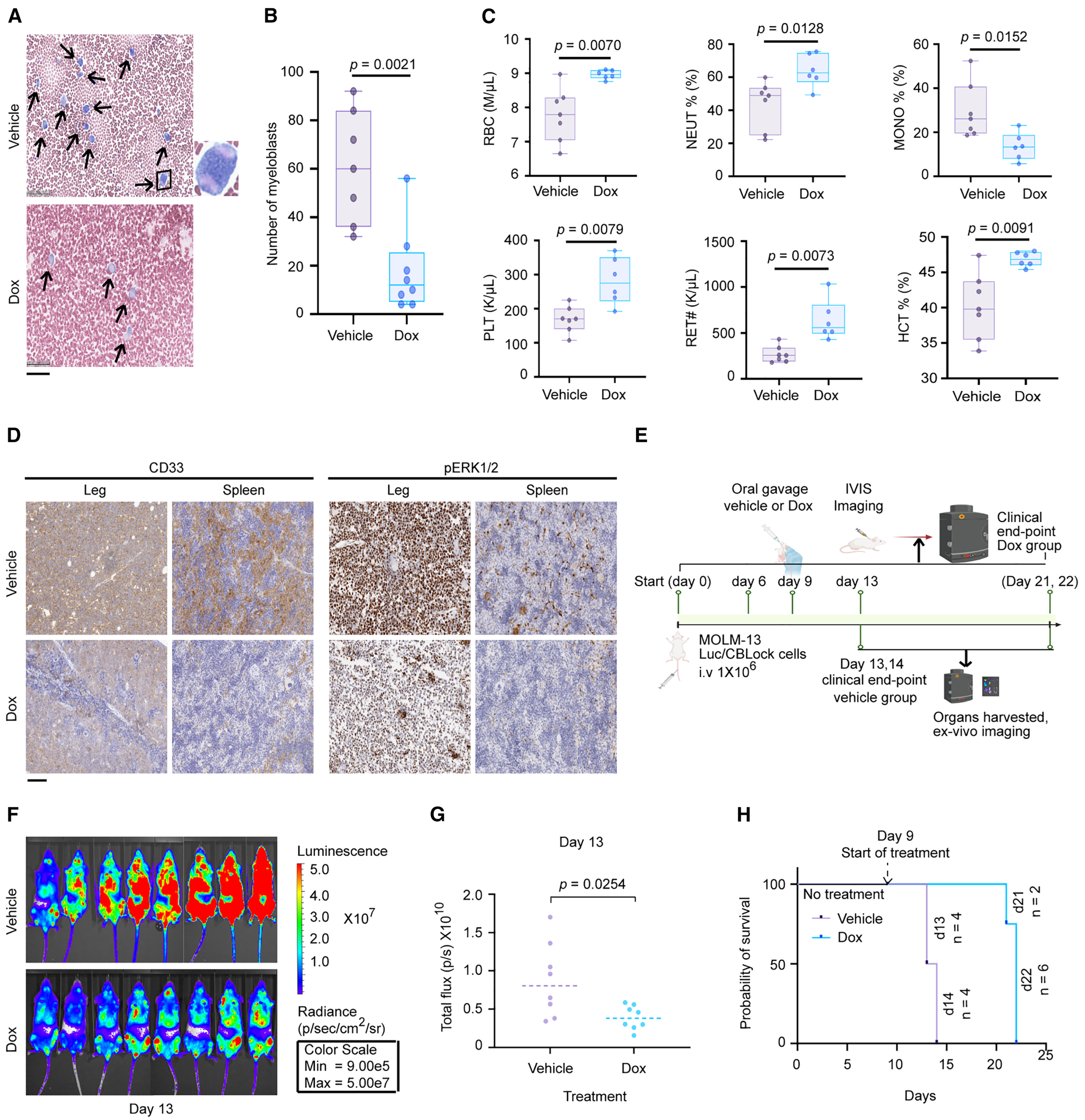

Figure 6. CBLock reverses the AML-like features in a murine model

(A) Representative images of Giemsa staining on whole blood smears of AML mice treated with vehicle (n = 7) or dox (n = 8) showing the presence of myeloblasts (bigger sized shown with arrows). Images were exported using Halo software at ×20 original magnification. The scale bar (bottom left) represents 100 μm in each panel. (B) Plot for (A), showing significantly higher number of myeloblasts in the vehicle-treated mice blood as compared with dox-treated mice blood. Data points represent individual mice blood harvested at clinical endpoint (day 12) for vehicle- (n = 7) and timed endpoint (day 21) for dox- (n = 8) treated mice, the error bars represent ±SD. (C) Complete blood count analyses of vehicle- (n = 7) and dox- (n = 6, reason for exclusion of two samples provided in the materials and methods section) treated mice cardiac whole blood. Plots showing significant differences in red blood cell count (RBC), neutrophil percent (NEUT%), monocyte percent (MONO%), platelet count (PLT), reticulocyte count (RET), and hematocrit percent (HCT %) between vehicle- and dox-treated mouse blood. Data points represent individual mouse blood harvested at clinical endpoint (day 12) for vehicle (n = 7) and timed endpoint (day 21) for dox- (n = 6) treated mice, the error bars represent ±SD. (D) Representative examples of CD33 and pERK1/2 immunohistochemical staining of FFPE tissue sections from leg and spleen of vehicle- (n = 7) and dox- (n = 8) treated mice. Images were exported using Halo software at 20× original magnification. Scale bar (bottom left) represents 100 μm in each panel. (E) Schematic showing the experimental set up highlighting injection of MOLM-13$^{Luc/CBLock}$ cells into NSG mice,

(legend continued on next page)

The following constructs are described in previous studies[7,28,39]: pBabe Puro FLAG-CBL-WT, pBabe Puro FLAG-CBL-Y371S, pGEX4T1 GST-TEV CBL TKBD (47–355), pcDNA3.1 Myc-CBL, pcDNA3.1 Myc-CBL-70Z, pcDNA3.1 Myc-CBL-Y371S, pGEX4T1 His-GST-Thrombin CBL-WT (47–435), pGEX4T1 His-GST-Thrombin CBL-R139C D435C (47–435), pET23d *Arabidopsis thaliana* Uba1, pRSF_1b UBE2D2, RSF_Duet mouse Src (84–526), and RSF_Duet His-TEV GGS-Ub.

Antibodies, reagents, and kits

Primary antibodies used in this study were mouse anti-FLT3 (Abcam, cat. no. ab263452, 1:50 for immunohistochemistry), rabbit anti-phospho-CBL-pY371 was generated by Eurogentec (1:500 for immunohistochemistry), mouse anti-CBL (Thermo Fischer Scientific, cat. no. MA5-15885, 1:1,000 for western blot, 1:500 for immunohistochemistry), rabbit anti-FLT3 (Cell signaling technology, cat. no. 3462, 1:1,000 for western blot, 1:100 for immunoprecipitation), rabbit anti-phospho-FLT3 (Cell Signaling Technology, cat. no. 3464, 1:1,000 for western blot), mouse anti-CBL (Santa Cruz Biotechnology, cat no. sc-1651AC, 1:50 for immunoprecipitation), mouse anti-Myc–tag (Cell Signaling, cat. no. 2276, 1:1,000 for western blot and 1:500 for immunoprecipitation), mouse anti-Actin (Santa Cruz, cat. no. sc-47778, 1:1,000 for western blot), rabbit anti-phospho-CBL-Y731 (Cell Signaling, cat. no. 3554, 1:1,000 for western blot), rabbit anti-EGFR (Merck Millipore, cat. no. 06–847, 1:1,000 for western blot and 1:500 for immunoprecipitation), mouse anti-GFP (Santa Cruz, cat. no. sc-81045, 1:1,000 for western blot), mouse anti-STAT5 (Abcam, cat. no. ab230670, 1:1,000 for western blot), rabbit anti-phospho-pSTAT5 (Abcam, cat. no. ab32364, 1:1,000 for western blot), rabbit anti-phospho-Erk1/2 (Cell Signaling Technology, cat no. 4370, 1:1,000 for western blot), mouse anti- Erk1/2 (Cell Signaling Technology, cat no. 9107, 1:1,000 for western blot), rabbit anti-phospho-AKT-Ser473 (Cell Signaling, cat. no. 9271, 1:1,000 for western blot), mouse anti-AKT (Cell Signaling, cat. no. 2920, 1:1,000 for western blot), rabbit anti-cleaved-Caspase-3 (Cell Signaling, cat. no.9664, 1:1,000 for western blot), rabbit anti-cleaved-PARP (Cell Signaling, cat. no., 5625, 1:1,000 for western blot), goat anti-GAPDH (Santa Cruz, cat. no. sc-20357, 1:1,000 for western blot), mouse anti-DYNLL1 (Santa Cruz, cat. no. sc-136287, 1:1,000 western blot), mouse anti-BUD31 (Santa Cruz, cat. no. sc-374163, 1:1,000 western blot), mouse anti-Ikaros (Santa Cruz, cat. no. sc-398265, 1:500 western blot), mouse anti-MRG1 (Abcam, cat. no. ab108345, 1:1,000 western blot), rabbit anti-RPL34 (Abcam, cat. no. ab122255, 1:1,000 for western blot), rabbit anti-firefly-luciferase (Abcam, cat. no. ab185923, 1:1,000 for western blot), CD33 (Abcam, cat. no. ab269456, 1:200 for immunohistochemistry), rabbit anti-phospho-p44/42 MAPK (pErk1/2) (Cell Signaling Technology, cat. No. 9101, 1:400 for immunohistochemistry). The secondary antibodies used in this study include goat anti-rabbit IRDye 680LT (LI-COR Biosciences, cat. no. 925–68021, 1:20,000 for western blot), goat anti-mouse IRDye 680LT (LI-COR Biosciences, cat. no. 925–68020, 1:20,000 for western blot), goat anti-rabbit IRDye 800CW (LI-COR Biosciences, cat. no. 925–32211, 1:15,000 for western blot), goat anti-mouse IRDye 800CW (LI-COR Biosciences, cat. no. 925–32210, 1:15,000 for western blot), and donkey anti-goat IRDye 800CW (LI-COR Biosciences, cat. no. 925–32214, 1:15,000 for western blot).

The compounds used in the study include growth factors: hEGF (Sigma-Aldrich) and FLT3L (R&D systems); chemicals: doxycycline (Sigma-Aldrich) and IVISBrite D-Luciferin Potassium salt (1g), P/*N* 122799; Revvity). The peptides (1–10), Antennapedia (RQIKIWFQN RRMKWKK), CBLock, cp-CBLock (RQIKIWFQNRRMKWKKGG SMTVEEMDSWIKSWDQR), cp-CBLock-I10A, FITC-cp-CBLock, pSYK (STVSFNP(pY)EPTGGPWG), cp-pSYK [DRQIKIWFQNR RMKWKKSTVSFNP(pY)EPTGGPWG], pZAP70 [TLNSDG(p)YT-PEPA] were obtained from Thermo Fisher Scientific and BioServ. GFP trap was purchased from Chromotek.

Mammalian cell maintenance

Mammalian cell lines HEK293 and NIH3T3 were obtained from ATCC while HeLa was obtained from EACC. The HeLa cells were cultured in RPMI while the HEK293 and NIH3T3 cells were cultured in DMEM. The cells were cultured as monolayers at 37°C in 5% CO_2. The leukemia cell lines expressing CBL-WT (K562, MOLT-4, RS4 (11), THP-1, Kasumi-1) were obtained from ATCC while leukemia cell lines expressing CBL mutants (MOLM-13, MOLM-14, GDM-1) were obtained from DSMZ. The leukemia cells and the EBV-B cells were cultured as suspensions at 37°C in 5% CO_2. All the cell lines were further supplemented with 20 mM L-glutamine, 0.1 mg/mL streptomycin, 100 units/mL penicillin, and 6 mg/L gentamycin, (Invitrogen, USA). The cells received the following % of serum: HEK293 and HeLa (10% FBS), all leukemia and EBV-B cells (20% FBS), and NIH3T3 (10% DBS). The cell lines were authenticated using GenePrint 10 System (Promega)'s short tandem repeat (STR) profiling and were tested regularly for mycoplasma.

Immunoblotting and co-immunoprecipitation

The cells were pelleted and lysed in IP lysis buffer (50 mM Tris-HCl pH 7.5, 150 mM NaCl, 1 mM EDTA, 1% IGEPAL CA-630, 10% glycerol, 0.5 mM DTT, and cOmplete protease inhibitor cocktail) to prepare the whole cell lysates. Immunoprecipitation and immunoblotting were performed using the lysates as described before.[28] For each regular immunoprecipitation reaction, 1 mg freshly prepared

bioluminescence imaging in the IVIS system, dox and vehicle treatment, euthanization of animals, and harvest of blood and organs. (F) Bioluminescence imaging of NSG mice intravenously injected with 100 μL of 1 × 10^6 $MOLM^{13Luc/CBLock}$ cells and treated with either vehicle (*n* = 8) or dox (*n* = 8) to monitor disease burden. (G) Tumor burdens in the two groups (*n* = 8 for each) of mice as a measure of total flux (p/s). Total flux was calculated from the region of interest (ROI) of the bioluminescent images of the animals, the dashed lines represent median values. (H) Survival probability of the AML mice treated with vehicle or dox represented as Kaplan-Meier curves. Half of the vehicle-treated mice (*n* = 4) reached humane clinical endpoint on day 13 while the rest (*n* = 4) on day 14. The dox-treated mice reached humane clinical endpoints on day 21 (*n* = 2) or day 22 (*n* = 6). The *p* values in (B), (C), and (G) were calculated using Student's t test (unpaired, parametric) with Welch's correction.

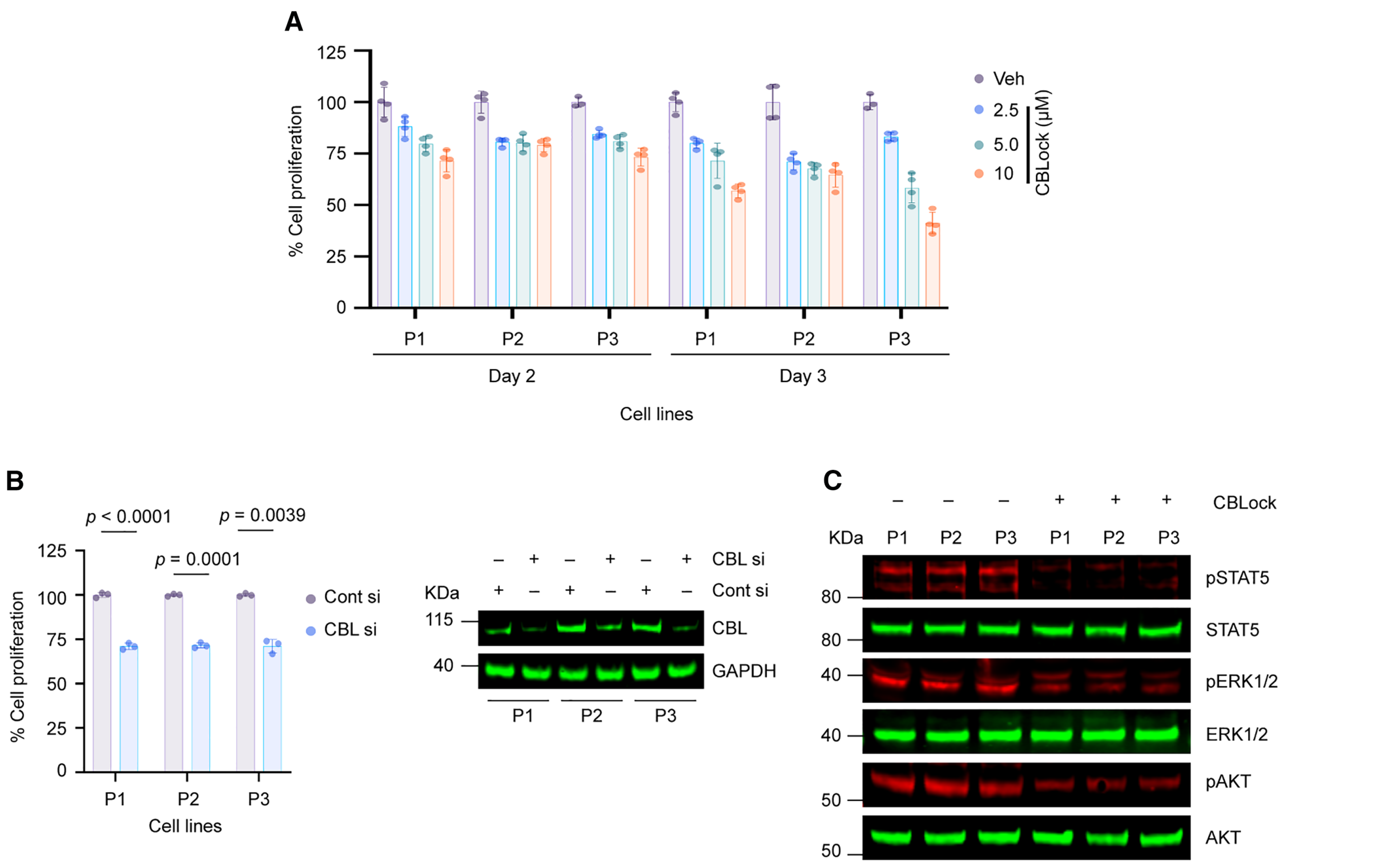

Figure 7. CBLock inhibits growth of patient-derived CBL mutant EBV-B cells and reduces RTK signaling

(A) Proliferation of CBL Y371C-expressing EBV-B cells from patients P1-P3 treated with vehicle (water) or different concentrations of cp-CBLock peptide were analyzed after 48 h and 72 h using Cell Titer-Glo and represented as percentage of cells treated with vehicle. Data are presented from four independent experiments while error bars represent ±SD (n = 4 independent experiments). (B) Proliferation of CBL Y371C-expressing EBV-B cells from patients P1–P3 knocked down with control siRNA (Cont si) or CBL siRNA (CBL si) and analyzed after 48 h using Cell Titer-Glo and represented as percentage of cells knocked down with Cont si. Data are presented from three independent experiments while the error bars represent ±SD (n = 3 independent experiments). Part of the siRNA transfected cells in (B) were grown separately, lysed, and lysates checked for expression of CBL and GAPDH (loading control). (C) Expression of pSTAT5, STAT5, pERK1/2, ERK1/2, pAKT, and AKT from lysates of EBV-B cells from P1–P3 treated with vehicle (water) or 5 μM cp-CBLock for 48 h. The experiment was repeated three times.

whole cell lysate was incubated with primary antibodies at 4 °C for 4 h on a rotatory shaker. This was followed by overnight incubation with 40 μL (50% slurry) Protein A Sepharose CL 4B beads under the same condition. Alternatively for each reaction, 1 mg freshly prepared whole cell lysate was incubated overnight with antibody- or immunoglobulin (Ig)G-conjugated agarose beads. The next day beads were washed three times with IP lysis buffer and once with PBS. For pulldown using GFP trap, the lysates were incubated with the trap beads for 1 h. This was followed by similar washing steps as mentioned before. The beads were then either processed for mass spectrometry or immunoprecipitated proteins were eluted at 95 °C in 40 μL 2X loading dye. A previous method was used to perform immunoblotting[51] and the immunoblots were scanned using an Odyssey CLx Imaging System (LI-COR Biosciences).

Mass spectrometry

Sample preparation

The empty vector (EV) and GFP-CBLock were expressed in HEK293 cells and immunoprecipitated with GFP trap. In another experiment, MOLM-14 cells were treated with 5 μM cp-CBLock or vehicle and cell lysates were prepared after 48 h. CBL was pulled down from the lysates using CBL antibody-conjugated or mouse IgG-conjugated agarose beads. Following immunoprecipitation, beads were washed three times with IP lysis buffer and once with PBS and resuspended in 100 mM ammonium bicarbonate buffer containing 2 M Urea and stored at −20°C. Samples from three biological replicates for each condition were digested "on beads" with Lys-C (Alpha Laboratories) and trypsin (Promega) as described previously.[52]

MS analysis

Separation of peptides resulting from trypsin and Lyc-C digestions was carried out by nanoscale C18 reverse-phase liquid chromatography in an EASY-nLC II 1200 (Thermo Scientific) coupled to an Orbitrap Fusion Lumos mass spectrometer (Thermo Scientific). A binary gradient was used to carry out elution at a flow rate of 300 nL/min, into an in-house packed 50 cm fused silica emitter (New Objective) with ReproSil-Pur C18-AQ, 1.9 μm resin (Dr Maisch GmbH), for 135 min total run-time duration. A column oven (sonation) integrated into the nanoelectrospray ion source (Thermo Scientific) was used to keep the packed emitter at 50 °C. A nanoelectrospray ion source was

used to electrospray the eluting peptides into the mass spectrometer. Air contaminants signal level was decreased using an active Background Ion Reduction Device (ESI Source Solutions). Data acquisition was carried out using the Xcalibur 4.2 software (Thermo Scientific). A full scan was acquired at a resolution of 60,000 at 200 m/z, over mass range of 350–1550 m/z. HCD fragmentation was triggered for the top 15 most intense ions detected in the full scan. For fragmentation, ions were isolated with a target of 7.5E4 ions, at a resolution of 15,000 at 200 m/z, for a maximum of 100 ms. Ions already selected for MS2 were dynamically excluded for 25 s.

MS data analysis

MaxQuant software[53] version 1.6.14.0 was used to process the MS raw data and searched with Andromeda search engine,[54] querying SwissProt[55] *Homo sapiens* (45,401 entries) and the sequence of CBLock peptide. First and main searches were performed with precursor mass tolerances of 20 ppm and 4.5 ppm, respectively, and MS/MS tolerance of 20 ppm. The minimum peptide length was set to six amino acids and specificity for trypsin cleavage was required. Cysteine carbamidomethylation was set as fixed modification, whereas Methionine oxidation and N-terminal acetylation were specified as variable modifications. The false discovery rate (FDR) for peptide, and protein site was set to 1%. Perseus software version 1.6.2.3[56] was used to analyze all MaxQuant outputs. Protein abundance was measured using label-free quantification (LFQ) intensities reported in the ProteinGroups.txt file. Only proteins quantified in all three replicates in at least one group, were measured according to the label-free quantification algorithm available in MaxQuant.[53] Missing values were imputed separately for each column. For the CBLock pulldown, significantly enriched proteins were selected using a permutation-based Student's t test with FDR set at 1%. For the comparison between endogenous and mutant CBL in both K562 and MOLM-14 cell lines, an ANOVA "Multiple sample test" was used with a truncation threshold value of 0.05 (Permutation-based FDR) and S0 = 1. Data for MOLM-14 has only been presented. The test was applied to the four groups considered. For data visualization, proteins that passed the ANOVA test were *Z* score normalized and hierarchical clustering analysis was performed using "Euclidian distance" for both rows and columns.

Short interfering RNA transfections

The siRNAs (30 nmol/L final concentration) against *CBL*, *MRG-1 (CITED2)*, *DYNLL1*, *BUD31*, *RPL34*, *Ikaros* (Santa Cruz Biotechnology) were transfected into the leukemia cells through nucleofection (nucleofector II, Amaxa) using nucleofector kits V and R (Lonza Bioscience). The cells were allowed to grow for 48 h followed by cell-based assays. The transfections of all other cell lines were done using either Lipofectamine-2000 (Thermo Fisher Scientific) or JetPEI (Polyplus-transfection) following manufacturers' protocols, and cells were harvested 48 h after transfection, unless otherwise mentioned.

Cell proliferation assay

The leukemia cells as mentioned in the figures were split into 96-well plates and treated with different vehicles or peptides. Alternatively, leukemia cells nucleofected with the desired siRNAs were seeded into 96-well plates. The cells were grown for either 48 or 72 h as indicated in the figures and assayed for proliferation using CellTiter-Glo luminescent cell viability assay (Promega). An equal volume of CellTiter-Glo was added to the medium in each well and mixed (500 rpm) for 2 min at room temperature in a Tecan multimode plate reader. After 10 min, luminescence was measured using a Tecan multimode plate reader with an integration time of 500 ms. GraphPad Prism was used to plot the obtained luminescence readings from the treated cells as percentage viable (proliferating) cells as compared to the untreated controls. The experiments were performed at least in three biological replicates.

Foci-formation assay

NIH3T3 cells (2.5×10^4) seeded onto 6-cm dishes were infected with the respective constructs on two consecutive days, followed by selection with 2.0 μg/mL puromycin or a combination of 1.0 μg/mL puromycin and 500 μg/mL G418 (geneticin) in fresh medium. The cells were cultured for 2–3 weeks until the appearance of visible foci, which were fixed in methanol followed by sulforhodamine B staining. Foci from three independent experiments were counted manually and GraphPad Prism 10.2.2 was used to perform the statistical analyses.

FACS analysis

For analyzing the peptide uptake, 2.5×10^5 MOLM-14 cells incubated with 1 or 2.5 μM FITC-cp-CBLock were allowed to grow for 24 or 48 h in 3-cm dishes. Cells were washed twice with PBS and resuspended in PBS. Single cell suspension was prepared by filtering through a 40-μm Falcon cell strainer. Dead cells were identified with Zombie yellow (BioLegend, cat no. 423103). Non-treated cells were used as fluorescence negative controls to set gating. For cell-cycle analysis, 2.5×10^5 MOLM-14 cells in 3-cm dishes were treated with either cp-CBLock-WT or cp-CBLock-I10A and allowed to grow for 72 h. The cells were washed twice with PBS and fixed in 70% ice-cold ethanol for 30 min at 4°C. Following ethanol fixation, the cells were washed again with PBS twice and treated with RNase followed by PI staining using the propidium iodide flow cytometry kit (Abcam, cat no. ab139418). Non-treated cells were used as negative controls to set gating. BD LSRFortessa using DIVA acquisition software was used to acquire the data while FlowJo software v9 461 (FlowJo, LLC) was used for data analysis.

Recombinant protein purification

Expression and purification of GST-CBL TKBD (47–355) was performed following an established method.[7] For crystallization, His-TEV Ub-peptide10-6xHis (non-cleavable His-tag at the C terminus) was purified using Ni-NTA affinity followed by TEV treatment. Ub-peptide10-6xHis was then purified by gel filtration chromatography. Glutathione-affinity chromatography was used to purify the GST-tagged ligands (GST, GST-TKBD) for surface plasmon resonance (SPR) experiments. The His-tagged analytes (His-Ub-peptide10 variants, His-GFP and His-GFP-peptide10) and His-iRFP-CBLock were purified by Ni-NTA affinity followed by gel filtration

chromatography. GST, GST-TKBD, GST-TKBD G306E for phage-display experiments and CBL-WT (47–435), CBL-diCys (47–435) for diCys disulfide linking experiments were purified using glutathione-affinity chromatography followed by on-beads cleavage by treatment with TEV protease and then gel filtration chromatography. Purification of c-Met (995–1360) followed simultaneous use of Ni-NTA affinity and glutathione-affinity chromatography, followed by thrombin cleavage. Cleaved c-Met was further purified using anion exchange and gel filtration chromatography.

Arabidopsis thaliana Uba1, UBE2D2, Ub, and CBL pY371 variants expression and purification have been described previously.[7] Bradford assay was used to determine the protein concentrations except for Ub, Ub-CBLock, and GFP-CBLock, for which the concentrations were determined by their absorbance at 280 nm.

Selection of binding peptides

The phage-displayed peptide library used in this study was described previously.[57] Protein immobilization and selections were performed according to established protocols.[58] Briefly, purified GST-tagged CBL TKBD or CBL TKBD-G306E were coated on 96-well MaxiSorp plates by adding 100 μL of 1 μM of each protein and incubated overnight at 4°C. Afterward, five rounds of selections using the phage-displayed 16-mer peptide library were applied against immobilized proteins, including the following steps. (1) Within the phage pool, each phage particle displays a unique peptide sequence and encapsulates the encoding DNA. (2) Protein-binding phages are captured with immobilized proteins. (3) Non-binding phages are washed away. (4) Bound phages are amplified by infection of a bacterial host. Individual phage with improved binding properties obtained from rounds 4 and 5 were identified by phage ELISA[59] and subjected to DNA sequencing of the phagemids to translate peptide sequences.

SPR binding assays

CM-5 chips (Cytiva) on a Biacore T200 at 25°C were used to perform the SPR experiments as described previously.[7] Running buffer (25 mM Tris-HCl, pH 7.6, 150 mM NaCl, 0.1 mg/mL BSA, 1 mM DTT, and 0.005% [v/v] Tween 20) was used to serially dilute the Ub-peptide and GFP-peptide fusion analytes and the same buffer but with 4% DMSO was used for the peptides in the absence of a fusion partner. Binding was calculated using concentration ranges as indicated in Figure S2. Signal differences between GST-protein and GST are reported and data analysis was performed using steady-state affinity analysis from Biacore Insight Software (Cytiva). GraphPad Prism was used to create the plots.

Disulfide bond formation assay

CBL variant mixtures were prepared at room temperature as described previously,[7] keeping aside a sample portion for zero-time point. Removal of DTT using a Zeba spin desalting column (Thermo Scientific) initiated the reactions. At indicated time points, samples were removed and quenched with 20 mM *N*-ethylmaleimide (NEM; Sigma) for 10 min and mixed with 4X SDS loading buffer. DTT (100 mM) was added to prepare the reduced samples. The samples were resolved by SDS-PAGE and the gels stained with Coomassie blue were scanned. The disulfide-bonded (D) and unmodified species (U) were quantified using the Image Studio software. Three independent experiments were performed, and curves for the disulfide bond fractions from each reaction were generated using GraphPad Prism.

Thermal shift assay

The thermal shift assay was performed following a previously described protocol.[60] HEK293 cells seeded onto 6-cm dishes were treated with the indicated peptides or vehicle and left for 24 h. The cells were then washed with PBS and pelleted. The cell pellets were washed and resuspended in 1.1 mL PBS in the presence of similar concentrations of peptides or vehicle. The samples split into different fractions of equal volume (100 μL each) were then exposed to different temperatures (37.0°C–65.0°C) for 3 min and cooled for 3 min at room temperature and placed in ice. The cells were lysed as described in immunoblotting before and whole cell lysates were prepared. SDS-PAGE using NuPAGE 4%–12% Bis-Tris gels (Thermo Fisher Scientific) was used to resolve the samples, and a previous method was used to perform immunoblotting.[51] The immunoblots were scanned using an Odyssey CLx Imaging System (LI-COR Biosciences) to quantify the relative CBL levels and assess its stability in the presence of the peptides or vehicle.

In vitro ubiquitination assay

A previously established protocol[7] was followed to perform the *in vitro* ubiquitination experiments. Briefly, buffer (50 mM Tris-HCl, pH 7.6, 50 mM NaCl, 5 mM $MgCl_2$ and 5 mM ATP) was used to perform the reactions using human UBA1 (0.5 μM), UBE2D2 (0.8 μM), Ub (5 μM), c-Met (2 μM), and pCBL 47–435 (0.2 μM) at room temperature in the presence or absence of the different peptides (25 μM). Aliquots were mixed with LDS-loading dye to stop the reactions either at fixed or indicated time points for different experiments. The samples were then resolved by SDS-PAGE, and gels stained with Coomassie blue were scanned. Autoubiquitination reactions were performed in the same buffer using human UBA1 (0.2 μM), UBE2D2 (5 μM), Ub (100 μM), GST-pCBL 47–435, or GST-pCBL 47–435 R420Q (0.8 μM) at room temperature. Aliquots were mixed with LDS-loading dye to stop the reactions at indicated time points. Samples were resolved by SDS-PAGE, and gels were stained with Coomassie blue.

Crystallization

Crystals were obtained by mixing CBL (47–355) (6.5 mg/mL) with Ub-peptide10 (10 mg/mL) in a 1:1 M ratio and equal volume of reservoir solution containing 0.1 M Bis-Tris Propane, pH 6.0, 0.2 M sodium fluoride, and 20% (v/v) PEG 3350 using a hanging drop vapor diffusion method at 19°C. The crystals were flash-frozen in 0.1 M Bis-Tris Propane, pH 6.0, 0.2 M sodium fluoride, 22% (v/v) PEG 3350, 20% (v/v) glycerol.

Data collection and structure determination

Data were collected at beamline I04 at Diamond Light Source (DLS), processed using xia2 pipeline,[61] and integrated with automated XDS.[62] Initial phases of TKBD-Ub-peptide10 complex were obtained by molecular replacement with PHASER[63] using the PDB entries for TKBD (PDB: 2Y1M) and Ub (PDB: 1UBQ). There are two molecules of TKBD-Ub-peptide10 complex in the asymmetric unit. The structures of peptide10 and the linker connecting to Ub were built manually. All models were built in COOT[64] and refined using PHENIX.[65] TKBD-Ub-peptide10 complex was refined to a resolution of 2.02 Å. All figure models were generated using PYMOL.

Preparation of MOLM-13$^{Luc/CBLock}$ variant cells

HEK293T cells seeded onto 10-cm dishes were transfected with pLenti-TK-Firefly (Addgene, cat no. 122277) along with psPAX2 (Addgene, plasmid no. 12260) and pMD2.G (Addgene, plasmid no. 12259). After 48 h, the viral particles were used to infect the MOLM-13 cells, followed by a reinfection 72 h later. Medium was changed and the cells were selected with 1.0 μg/mL puromycin. The cells were checked for luciferase expression through immunoblotting; luciferase activity was measured using ONE-Glo EX Luciferase Assay System (Promega) following the manufacturer's protocol. Fresh viral particles were generated in HEK293T cells transfected with pCW57 *Neo* iRFP-CBLock variants along with pMD2.G and psPAX2. MOLM-13 cells stably expressing firefly luciferase were further infected with the fresh viral particles on 2 consecutive days, followed by selection with 500 μg/mL G418 (geneticin). After generating the stable MOLM-13$^{Luc/CBLock}$ variant cell lines, the cells were induced with 1.0 or 2.0 μg/mL doxycycline after every 2 days for 8 or 10 days. Expression of CBLock variants was confirmed by the presence of iRFP in the cells when scanned in the Odyssey CLx Imaging System (LI-COR Biosciences). Alternatively, the cells were lysed, and immunoblotting was performed to check for iRFP.

AML animal model and bioluminescence imaging

All animal procedures adhered to ethical guidelines and received approval through the UK Home Office Project License (PPL 70/8645 and PP6345023) and the Animal Welfare and Ethical Review Body (AWERB) of the University of Glasgow. The study was conducted in strict compliance with Animals Scientific Procedure Act 1986 and ARRIVE guidelines were followed in the reporting of *in vivo* experiments. Female NSG (8–9 weeks old) mice were purchased from Charles River Laboratories (UK). Animals were housed with *ad libitum* access to food and water, under a 12-h day/night cycle at 21 °C at 55% humidity in individual ventilated cages with environmental enrichment. Mice (9–10 weeks old) were intravenously injected with either 1×10^6 or 5×10^6 mycoplasma-free MOLM-13$^{Luc/CBLock}$ (with constitutive luciferase expression along with doxycycline inducible iRFP-CBLock expression) cells. Thirty minutes after injection, the animals were imaged for the uptake of cells through bioluminescence imaging using the luciferase substrate (D-Luciferin) in an IVIS Spectrum (IS1121N5602; Revvity UK LTD) *in vivo* imaging system. In the first experiment, eight mice were randomly assigned to receive 100 μL of 20 mg/mL doxycycline (Sigma-Aldrich) daily via oral gavage, while the remaining eight were part of the vehicle control group receiving distilled water by oral gavage. The animals in both groups received their treatment on the same day as cell transplantation. Tumor burden/disease progression was monitored through bioluminescence imaging as described above and the animals were weighed daily. The vehicle mice were euthanized on day 12 on reaching humane clinical endpoint while the dox-treated mice were euthanized on day 21 as a timed endpoint. For bioluminescence imaging of organs, organs were harvested, and bioluminescence measured in each organ to assess the leukemic cell infiltration. For cohort two, treatment (as above) was started only when animals presented with tumor burden as observed through bioluminescence imaging [total flux (p/s) $\geq 10^5$]. Both the vehicle and dox-treated mice were euthanized on reaching humane clinical endpoints. Kaplan-Meier method was used to plot the survival curves for the mice receiving dox or vehicle treatment.

Histology, Giemsa staining, and blood cell count analysis

Major organs were harvested and fixed in 10% buffered neutral formalin, which were then dehydrated and embedded. Hematoxylin and eosin (H&E) staining was used to assess the impact of CBLock overexpression in the animals and the extent of leukemic infiltration in different organs on 4 μm formalin-fixed paraffin-embedded sections (FFPE) that had previously been placed in the oven overnight at 37°C then for 60°C for 2 h.

Smears were made onto glass slides from whole blood of the euthanized mice. Giemsa staining of the blood smears was performed to analyze the mice whole blood for the presence of myeloblasts using the Giemsa Stain Kit (Abcam, cat. no. ab150670) following the manufacturer's protocol. Whole cardiac blood was drawn from the animals postmortem and whole blood cell counts were immediately analyzed in a ProCyte Dx Hematology Analyzer (IDEXX) following the manufacturer's instructions. Results were not attained from two of the dox-treated mice for which samples failed quality control and these were not included in further analysis.

Tissue microarray and immunohistochemistry

Immune multiple organs tumor and normal tissue array (Tissue Array, US Biomax; MC1081) contained 20 leukemia (including primitive B lymphoblastic leukemia, B cell chronic lymphocytic leukemia, chronic B cell leukemia, chronic myelocytic leukemia, acute granulocytic leukemia, and hairy cell leukemia) patient samples and eight different normal (including lymph node, spleen, bone marrow, and thymus gland) tissue samples. Following immunohistochemistry (IHC), expression levels of FLT3, CBL, and CBL pY371 were analyzed making use of the Halo software, and representative images were exported. Only the intact cores (actual core numbers indicated in figure legends) were used to manually perform semi-quantitative H-scoring using a score range of 300 (100% strong staining) to 0 (negative) was performed manually.[66] Patient consent details and ethical standards can be obtained from TissueArray.

Bone tissue was fixed in 10% neutral buffered formalin before being decalcified in an EDTA solution at 40°C for differing amounts of time depending on tissue type (legs, 7 days, ribs/spine, 2 days). Tissue was then processed through a standard overnight histology tissue processing cycle before being orientated and embedded in histology wax. IHC staining was performed on 4 μm FFPE sections that had previously been ovened overnight at 37°C then for 60°C for 2 h.

FFPE sections for phospho-p44/42 MAPK (pERK1/2) staining were loaded into an Agilent pre-treatment module to be dewaxed and to undergo heat-induced epitope retrieval (HIER) using high target retrieval solution (K8004, Agilent) where sections were heated to 97°C for 20 min. After HIER, the sections were rinsed in flex wash buffer (K8007, Agilent) prior to being loaded onto the Agilent autostainer. The sections underwent peroxidase blocking (S2023, Agilent) for 5 min and rinsed with flex buffer. The antibody (pERK1/2) was applied at an optimized dilution for 30 min before washing with flex wash buffer. Rabbit Envision was next applied for 30 min before washing with flex wash buffer then applying Liquid DAB (K3468, Agilent) for 10 min. Sections were washed in water and counterstained with hematoxylin z (RBA-4201-00A, CellPath). FFPE sections were stained on a Leica Bond Rx autostainer for c-CBL, CD33, FLT3, and phospho-CBL (pCBL). FFPE sections underwent on-board dewaxing (AR9222, Leica) and epitope retrieval using appropriate epitope retrieval solution (ER). Sections for c-CBL and pCBL staining underwent retrieval using ER1 (AR9661, Leica) where solution was applied for 40 min at 95°C. Sections for CD33 and FLT3 underwent retrieval using ER2 solution (AR9640, Leica) for 20 min at 95°C. Sections were rinsed with Leica wash buffer (AR9590, Leica) before peroxidase block was performed using an Intense R kit (DS9263, Leica) for 5 min. Sections were rinsed with wash buffer (AR9590, Leica) before application of primary antibody at an optimal dilution. The sections were rinsed with wash buffer before application of appropriate secondary antibody (cCBL, Mouse Envision; K4001, Agilent) (CD33, FLT3, pCBL, Rabbit Envision; K4003, Agilent) for 30 min. The sections were rinsed with wash buffer, visualized using DAB and counterstained with hematoxylin in the Intense R kit. The IHC-stained sections were rinsed in tap water, dehydrated through a series of graded alcohols, and placed in xylene. The stained sections were coverslipped in xylene using DPX mountant (SEA-1300-00A, CellPath). The slides were scanned using a Leica SCN400F slide scanner (Leica Biosystems, Wetzlar, Germany). Representative images were exported using Halo software and analyzed.

Human patient sample and immortalized EBV-B cells

Informed consent was obtained in the country of residence for each patient (France) in accordance with local regulations and with institutional review board (IRB) approval. The physicians caring for the patients completed a detailed questionnaire recording demographic data, clinical features, and biological and microbiological results, and the data were sent to J.C.B.

Quantification and statistical analysis

Statistical analyses were performed with GraphPad Prism 10.2.2. Student's t tests (unpaired, parametric) with Welch's correction were performed to analyze statistical significance with p values < 0.05 accepted as statistically significant unless otherwise mentioned. The error bars present in the figures represent ±SD. Representative images are shown, and the experiments were performed three times unless otherwise stated.

DATA AVAILABILITY

Coordinate and structure factors for TKBD-Ub-peptide10 have been deposited in the Protein DataBank with accession code PDB: 9ERZ. Raw mass spectrometry proteomics data have been deposited to the ProteomeXchange Consortium (http://proteomecentral.proteomexchange.org) via the PRIDE partner repository with the dataset identifiers PRIDE: PXD051178, PXD051179. All data generated during this study are included in this article (and its supplemental files) or are available from the corresponding author upon request. All raw gel and western blot images generated during this study have been deposited to Mendeley Data: https://doi.org/10.17632/694w27wyjm.1.

ACKNOWLEDGMENTS

We thank DLS for access to beamline I04 (proposal mx16258) that contributed to the results presented here; and Mads Gabrielsen for input in analyzing the initial low-resolution structure of TKBD-Ub-peptide10 complex. We acknowledge support from the BSU Biological Services Unit, Molecular Technologies and Histology labs at the CRUK Scotland Institute. We would also like to thank Dimitris Athineos (CRUK Scotland Institute) for assistance with the *in vivo* experiments and Catherine Winchester (CRUK Scotland Institute) for critically reviewing the manuscript. Schematic figures for the *in vivo* experiments and the graphical abstract were created using BioRender.com. In memoriam of our esteemed co-author Dr. Wei Zhang, we express sincere gratitude for his passionate contribution that was instrumental in shaping the direction of this research work.

This work was supported by the CRUK Scotland Institute's Advanced Technology Facilities (core funded by CRUK with grants A17196 and A31287), European Research Council (ERC) under the European Union's Horizon 2020 research and innovation program (grant agreement n 647849), Cancer Research UK grant (A23278/A29256), and Glasgow Children's Hospital Charity to D.T.H. K.B. is supported by CRUK (A17196, A29799, and A31287). S.Z. was supported by CRUK SI Core Funding (A29800). W.Z. was supported by a Cancer Research Society/BMO Bank of Montreal Scholarship for the Next Generation of Scientists. S.S.S. is supported by a Genome Canada Disruptive Innovation in Genomics grant (OGI-119).

AUTHOR CONTRIBUTIONS

S.F.A. and D.T.H. conceived the project, determined the structure of TKBD-Ub-peptide10 complex, and wrote the manuscript. S.F.A., A.P., and K.A.M. generated constructs, and purified proteins. S.F.A. conducted the cell culture experiments and performed protein crystallization. W.Z. and S.S.S. performed phage-displayed peptide binding selection and analysis. G.J.S. and L.B. performed and analyzed SPR experiments. S.L. and S.F.A. performed and analyzed MS proteomic experiments. S.Z. supervised MS proteomic experiments. L.R. and S.F.A. performed and analyzed the FACS experiments. K.B. and L.M. supervised and conceived the *in vivo* experiments. J.A. and S.F.A. performed and analyzed the *in vivo* experiments. C.N. processed harvested organs and performed IHC. J.B. and J.C.B. acquired the human samples and generated the immortalized EBV-B cells. All authors commented on the manuscript.

DECLARATION OF INTERESTS

The authors declare no competing interests.

SUPPLEMENTAL INFORMATION

Supplemental information can be found online at https://doi.org/10.1016/j.ymthe.2025.04.042.

REFERENCES

1. Ng, C., Jackson, R.A., Buschdorf, J.P., Sun, Q., Guy, G.R., and Sivaraman, J. (2008). Structural basis for a novel intrapeptidyl H-bond and reverse binding of c-Cbl-TKB domain substrates. EMBO J. *27*, 804–816. https://doi.org/10.1038/emboj.2008.18.

2. Petrelli, A., Gilestro, G.F., Lanzardo, S., Comoglio, P.M., Migone, N., and Giordano, S. (2002). The endophilin-CIN85-Cbl complex mediates ligand-dependent downregulation of c-Met. Nature *416*, 187–190. https://doi.org/10.1038/416187a.

3. Soubeyran, P., Kowanetz, K., Szymkiewicz, I., Langdon, W.Y., and Dikic, I. (2002). Cbl-CIN85-endophilin complex mediates ligand-induced downregulation of EGF receptors. Nature *416*, 183–187. https://doi.org/10.1038/416183a.

4. Yokouchi, M., Kondo, T., Sanjay, A., Houghton, A., Yoshimura, A., Komiya, S., Zhang, H., and Baron, R. (2001). Src-catalyzed phosphorylation of c-Cbl leads to the interdependent ubiquitination of both proteins. J. Biol. Chem. *276*, 35185–35193. https://doi.org/10.1074/jbc.M102219200.

5. Paul, M.K., and Mukhopadhyay, A.K. (2004). Tyrosine kinase - Role and significance in Cancer. Int. J. Med. Sci. *1*, 101–115.

6. Zheng, N., Wang, P., Jeffrey, P.D., and Pavletich, N.P. (2000). Structure of a c-Cbl-UbcH7 complex: RING domain function in ubiquitin-protein ligases. Cell *102*, 533–539.

7. Dou, H., Buetow, L., Hock, A., Sibbet, G.J., Vousden, K.H., and Huang, D.T. (2012). Structural basis for autoinhibition and phosphorylation-dependent activation of c-Cbl. Nat. Struct. Mol. Biol. *19*, 184–192. https://doi.org/10.1038/nsmb.2231.

8. Dou, H., Buetow, L., Sibbet, G.J., Cameron, K., and Huang, D.T. (2013). Essentiality of a non-RING element in priming donor ubiquitin for catalysis by a monomeric E3. Nat. Struct. Mol. Biol. *20*, 982–986. https://doi.org/10.1038/nsmb.2621.

9. Kassenbrock, C.K., and Anderson, S.M. (2004). Regulation of ubiquitin protein ligase activity in c-Cbl by phosphorylation-induced conformational change and constitutive activation by tyrosine to glutamate point mutations. J. Biol. Chem. *279*, 28017–28027. https://doi.org/10.1074/jbc.M404114200.

10. Levkowitz, G., Waterman, H., Ettenberg, S.A., Katz, M., Tsygankov, A.Y., Alroy, I., Lavi, S., Iwai, K., Reiss, Y., Ciechanover, A., et al. (1999). Ubiquitin ligase activity and tyrosine phosphorylation underlie suppression of growth factor signaling by c-Cbl/Sli-1. Mol. Cell *4*, 1029–1040.

11. Kales, S.C., Ryan, P.E., Nau, M.M., and Lipkowitz, S. (2010). Cbl and human myeloid neoplasms: the Cbl oncogene comes of age. Cancer Res. *70*, 4789–4794. https://doi.org/10.1158/0008-5472.CAN-10-0610.

12. Clara, J.A., Sallman, D.A., and Padron, E. (2016). Clinical management of myelodysplastic syndrome/myeloproliferative neoplasm overlap syndromes. Cancer Biol. Med. *13*, 360–372. https://doi.org/10.20892/j.issn.2095-3941.2016.0043.

13. Schwaab, J., Ernst, T., Erben, P., Rinke, J., Schnittger, S., Ströbel, P., Metzgeroth, G., Mossner, M., Haferlach, T., Cross, N.C.P., et al. (2012). Activating CBL mutations are associated with a distinct MDS/MPN phenotype. Ann. Hematol. *91*, 1713–1720. https://doi.org/10.1007/s00277-012-1521-3.

14. Hou, H.A., Tsai, C.H., Lin, C.C., Chou, W.C., Kuo, Y.Y., Liu, C.Y., Tseng, M.H., Peng, Y.L., Liu, M.C., Liu, C.W., et al. (2018). Incorporation of mutations in five genes in the revised International Prognostic Scoring System can improve risk stratification in the patients with myelodysplastic syndrome. Blood Cancer J. *8*, 39. https://doi.org/10.1038/s41408-018-0074-7.

15. Leardini, D., Messelodi, D., Muratore, E., Baccelli, F., Bertuccio, S.N., Anselmi, L., Pession, A., and Masetti, R. (2022). Role of CBL Mutations in Cancer and Non-Malignant Phenotype. Cancers (Basel) *14*, 839. https://doi.org/10.3390/cancers14030839.

16. Bamford, S., Dawson, E., Forbes, S., Clements, J., Pettett, R., Dogan, A., Flanagan, A., Teague, J., Futreal, P.A., Stratton, M.R., and Wooster, R. (2004). The COSMIC (Catalogue of Somatic Mutations in Cancer) database and website. Br. J. Cancer *91*, 355–358. https://doi.org/10.1038/sj.bjc.6601894.

17. Caligiuri, M.A., Briesewitz, R., Yu, J., Wang, L., Wei, M., Arnoczky, K.J., Marburger, T.B., Wen, J., Perrotti, D., Bloomfield, C.D., and Whitman, S.P. (2007). Novel c-CBL and CBL-b ubiquitin ligase mutations in human acute myeloid leukemia. Blood *110*, 1022–1024. https://doi.org/10.1182/blood-2006-12-061176.

18. Bandi, S.R., Brandts, C., Rensinghoff, M., Grundler, R., Tickenbrock, L., Köhler, G., Duyster, J., Berdel, W.E., Müller-Tidow, C., Serve, H., et al. (2009). E3 ligase-defective Cbl mutants lead to a generalized mastocytosis and myeloproliferative disease. Blood *114*, 4197–4208. https://doi.org/10.1182/blood-2008-12-190934.

19. Niemeyer, C.M., Kang, M.W., Shin, D.H., Furlan, I., Erlacher, M., Bunin, N.J., Bunda, S., Finklestein, J.Z., Gorr, T.A., Mehta, P., et al. (2010). Germline CBL mutations cause developmental abnormalities and predispose to juvenile myelomonocytic leukemia. Nat. Genet. *42*, 794–800. https://doi.org/10.1038/ng.641.

20. Sanada, M., Suzuki, T., Shih, L.Y., Otsu, M., Kato, M., Yamazaki, S., Tamura, A., Honda, H., Sakata-Yanagimoto, M., Kumano, K., et al. (2009). Gain-of-function of mutated C-CBL tumour suppressor in myeloid neoplasms. Nature *460*, 904–908. https://doi.org/10.1038/nature08240.

21. Sargin, B., Choudhary, C., Crosetto, N., Schmidt, M.H.H., Grundler, R., Rensinghoff, M., Thiessen, C., Tickenbrock, L., Schwäble, J., Brandts, C., et al. (2007). Flt3-dependent transformation by inactivating c-Cbl mutations in AML. Blood *110*, 1004–1012. https://doi.org/10.1182/blood-2007-01-066076.

22. Nadeau, S., An, W., Palermo, N., Feng, D., Ahmad, G., Dong, L., Borgstahl, G.E., Natarajan, A., Naramura, M., Band, V., and Band, H. (2012). Oncogenic Signaling by Leukemia-Associated Mutant Cbl Proteins. Biochem. Anal. Biochem. *Suppl 6*, 7921. https://doi.org/10.4172/2161-1009.S6-001.

23. Nakata, Y., Ueda, T., Nagamachi, A., Yamasaki, N., Ikeda, K.I., Sera, Y., Takubo, K., Kanai, A., Oda, H., Sanada, M., et al. (2017). Acquired expression of Cbl(Q367P) in mice induces dysplastic myelopoiesis mimicking chronic myelomonocytic leukemia. Blood *129*, 2148–2160. https://doi.org/10.1182/blood-2016-06-724658.

24. Rathinam, C., Thien, C.B.F., Flavell, R.A., and Langdon, W.Y. (2010). Myeloid leukemia development in c-Cbl RING finger mutant mice is dependent on FLT3 signaling. Cancer Cell *18*, 341–352. https://doi.org/10.1016/j.ccr.2010.09.008.

25. Fernandes, M.S., Reddy, M.M., Croteau, N.J., Walz, C., Weisbach, H., Podar, K., Band, H., Carroll, M., Reiter, A., Larson, R.A., et al. (2010). Novel oncogenic mutations of CBL in human acute myeloid leukemia that activate growth and survival pathways depend on increased metabolism. J. Biol. Chem. *285*, 32596–32605. https://doi.org/10.1074/jbc.M110.106161.

26. Nadeau, S.A., An, W., Mohapatra, B.C., Mushtaq, I., Bielecki, T.A., Luan, H., Zutshi, N., Ahmad, G., Storck, M.D., Sanada, M., et al. (2017). Structural Determinants of the Gain-of-Function Phenotype of Human Leukemia-associated Mutant CBL Oncogene. J. Biol. Chem. *292*, 3666–3682. https://doi.org/10.1074/jbc.M116.772723.

27. Thien, C.B., Walker, F., and Langdon, W.Y. (2001). RING finger mutations that abolish c-Cbl-directed polyubiquitination and downregulation of the EGF receptor are insufficient for cell transformation. Mol. Cell *7*, 355–365.

28. Ahmed, S.F., Buetow, L., Gabrielsen, M., Lilla, S., Sibbet, G.J., Sumpton, D., Zanivan, S., Hedley, A., Clark, W., and Huang, D.T. (2021). E3 ligase-inactivation rewires CBL interactome to elicit oncogenesis by hijacking RTK-CBL-CIN85 axis. Oncogene *40*, 2149–2164. https://doi.org/10.1038/s41388-021-01684-x.

29. Belizaire, R., Koochaki, S.H.J., Udeshi, N.D., Vedder, A., Sun, L., Svinkina, T., Hartigan, C., McConkey, M., Kovalcik, V., Bizuayehu, A., et al. (2021). CBL mutations drive PI3K/AKT signaling via increased interaction with LYN and PIK3R1. Blood *137*, 2209–2220. https://doi.org/10.1182/blood.2020006528.

30. Andoniou, C.E., Lill, N.L., Thien, C.B., Lupher, M.L., Jr., Ota, S., Bowtell, D.D., Scaife, R.M., Langdon, W.Y., and Band, H. (2000). The Cbl proto-oncogene product negatively regulates the Src-family tyrosine kinase Fyn by enhancing its degradation. Mol. Cel. Biol. *20*, 851–867.

31. Buday, L., Khwaja, A., Sipeki, S., Faragó, A., and Downward, J. (1996). Interactions of Cbl with two adapter proteins, Grb2 and Crk, upon T cell activation. J. Biol. Chem. *271*, 6159–6163.

32. Jozic, D., Cárdenes, N., Deribe, Y.L., Moncalián, G., Hoeller, D., Groemping, Y., Dikic, I., Rittinger, K., and Bravo, J. (2005). Cbl promotes clustering of endocytic adaptor proteins. Nat. Struct. Mol. Biol. *12*, 972–979. https://doi.org/10.1038/nsmb1000.

33. Meisner, H., Conway, B.R., Hartley, D., and Czech, M.P. (1995). Interactions of Cbl with Grb2 and phosphatidylinositol 3'-kinase in activated Jurkat cells. Mol. Cel. Biol. *15*, 3571–3578.

34. Miura-Shimura, Y., Duan, L., Rao, N.L., Reddi, A.L., Shimura, H., Rottapel, R., Druker, B.J., Tsygankov, A., Band, V., and Band, H. (2003). Cbl-mediated ubiquitinylation and negative regulation of Vav. J. Biol. Chem. *278*, 38495–38504. https://doi.org/10.1074/jbc.M305656200.

35. Rao, N., Miyake, S., Reddi, A.L., Douillard, P., Ghosh, A.K., Dodge, I.L., Zhou, P., Fernandes, N.D., and Band, H. (2002). Negative regulation of Lck by Cbl ubiquitin ligase. Proc. Natl. Acad. Sci. USA *99*, 3794–3799. https://doi.org/10.1073/pnas.062055999.

36. Reedquist, K.A., Fukazawa, T., Panchamoorthy, G., Langdon, W.Y., Shoelson, S.E., Druker, B.J., and Band, H. (1996). Stimulation through the T cell receptor induces Cbl association with Crk proteins and the guanine nucleotide exchange protein C3G. J. Biol. Chem. *271*, 8435–8442.

37. Matsuo, Y., MacLeod, R.A., Uphoff, C.C., Drexler, H.G., Nishizaki, C., Katayama, Y., Kimura, G., Fujii, N., Omoto, E., Harada, M., and Orita, K. (1997). Two acute monocytic leukemia (AML-M5a) cell lines (MOLM-13 and MOLM-14) with interclonal phenotypic heterogeneity showing MLL-AF9 fusion resulting from an occult chromosome insertion, ins(11;9)(q23;p22p23). Leukemia *11*, 1469–1477. https://doi.org/10.1038/sj.leu.2400768.

38. Makishima, H., Sugimoto, Y., Szpurka, H., Clemente, M.J., Ng, K.P., Muramatsu, H., O'Keefe, C., Saunthararajah, Y., and Maciejewski, J.P. (2012). CBL mutation-related patterns of phosphorylation and sensitivity to tyrosine kinase inhibitors. Leukemia *26*, 1547–1554. https://doi.org/10.1038/leu.2012.7.

39. Buetow, L., Tria, G., Ahmed, S.F., Hock, A., Dou, H., Sibbet, G.J., Svergun, D.I., and Huang, D.T. (2016). Casitas B-lineage lymphoma linker helix mutations found in myeloproliferative neoplasms affect conformation. BMC Biol. *14*, 76. https://doi.org/10.1186/s12915-016-0298-6.

40. Wirnsberger, G., Zwolanek, F., Asaoka, T., Kozieradzki, I., Tortola, L., Wimmer, R. A., Kavirayani, A., Fresser, F., Baier, G., Langdon, W.Y., et al. (2016). Inhibition of CBLB protects from lethal Candida albicans sepsis. Nat. Med. *22*, 915–923. https://doi.org/10.1038/nm.4134.

41. Layssol-Lamour, C.J., Sarry, J.E., Braun, J.P.D., Trumel, C., and Bourgès-Abella, N. H. (2021). Reference Values for Hematology, Plasma Biochemistry, Bone Marrow Cytology and Bone Histology of NOD.Cg-Prkdc(scid) Il2rg(tm1Wjl)/SzJ Immunodeficient Mice. J. Am. Assoc. Lab. Anim. Sci. *60*, 4–17. https://doi.org/10.30802/AALAS-JAALAS-20-000020.

42. Mfuh, A.M., Boerth, J.A., Bommakanti, G., Chan, C., Chinn, A.J., Code, E., Fricke, P. J., Giblin, K.A., Gohlke, A., Hansel, C., et al. (2024). Discovery, Optimization, and Biological Evaluation of Arylpyridones as Cbl-b Inhibitors. J. Med. Chem. *67*, 1500–1512. https://doi.org/10.1021/acs.jmedchem.3c02083.

43. Boerth, J.A., Chinn, A.J., Schimpl, M., Bommakanti, G., Chan, C., Code, E.L., Giblin, K.A., Gohlke, A., Hansel, C.S., Jin, M., et al. (2023). Discovery of a Novel Benzodiazepine Series of Cbl-b Inhibitors for the Enhancement of Antitumor Immunity. ACS Med. Chem. Lett. *14*, 1848–1856. https://doi.org/10.1021/acsmedchemlett.3c00439.

44. Kimani, S.W., Perveen, S., Szewezyk, M., Zeng, H., Dong, A., Li, F., Ghiabi, P., Li, Y., Chau, I., Arrowsmith, C.H., et al. (2023). The co-crystal structure of Cbl-b and a small-molecule inhibitor reveals the mechanism of Cbl-b inhibition. Commun. Biol. *6*, 1272. https://doi.org/10.1038/s42003-023-05655-8.

45. Ochi, A., Abe, T., Nakao, R., Yamamoto, Y., Kitahata, K., Takagi, M., Hirasaka, K., Ohno, A., Teshima-Kondo, S., Taesik, G., et al. (2015). N-myristoylated ubiquitin ligase Cbl-b inhibitor prevents on glucocorticoid-induced atrophy in mouse skeletal muscle. Arch. Biochem. Biophys. *570*, 23–31. https://doi.org/10.1016/j.abb.2015.02.006.

46. Hu, L.B., Hu, X.Q., Zhang, Q., You, Q.D., and Jiang, Z.Y. (2021). An affinity prediction approach for the ligand of E3 ligase Cbl-b and an insight into substrate binding pattern. Bioorg. Med. Chem. *38*, 116130. https://doi.org/10.1016/j.bmc.2021.116130.

47. Weisberg, E., Meng, C., Case, A.E., Tiv, H.L., Gokhale, P.C., Toure, A.A., Buhrlage, S., Liu, X., Wang, J., Gray, N., et al. (2020). The combination of FLT3 and SYK kinase inhibitors is toxic to leukaemia cells with CBL mutations. J. Cel. Mol. Med. *24*, 2145–2156. https://doi.org/10.1111/jcmm.14820.

48. Mayerhofer, C., Niemeyer, C.M., and Flotho, C. (2021). Current Treatment of Juvenile Myelomonocytic Leukemia. J. Clin. Med. *10*, 3084. https://doi.org/10.3390/jcm10143084.

49. Sato, T., Yang, X., Knapper, S., White, P., Smith, B.D., Galkin, S., Small, D., Burnett, A., and Levis, M. (2011). FLT3 ligand impedes the efficacy of FLT3 inhibitors in vitro and in vivo. Blood *117*, 3286–3293. https://doi.org/10.1182/blood-2010-01-266742.

50. Tan, Y.S., Lane, D.P., and Verma, C.S. (2016). Stapled peptide design: principles and roles of computation. Drug Discov. Today *21*, 1642–1653. https://doi.org/10.1016/j.drudis.2016.06.012.

51. Magnussen, H.M., Ahmed, S.F., Sibbet, G.J., Hristova, V.A., Nomura, K., Hock, A.K., Archibald, L.J., Jamieson, A.G., Fushman, D., Vousden, K.H., et al. (2020). Structural basis for DNA damage-induced phosphoregulation of MDM2 RING domain. Nat. Commun. *11*, 2094. https://doi.org/10.1038/s41467-020-15783-y.

52. Hubner, N.C., Bird, A.W., Cox, J., Splettstoesser, B., Bandilla, P., Poser, I., Hyman, A., and Mann, M. (2010). Quantitative proteomics combined with BAC TransgeneOmics reveals in vivo protein interactions. J. Cell Biol. *189*, 739–754. https://doi.org/10.1083/jcb.200911091.

53. Cox, J., Hein, M.Y., Luber, C.A., Paron, I., Nagaraj, N., and Mann, M. (2014). Accurate proteome-wide label-free quantification by delayed normalization and maximal peptide ratio extraction, termed MaxLFQ. Mol. Cel. Proteomics *13*, 2513–2526. https://doi.org/10.1074/mcp.M113.031591.

54. Cox, J., Neuhauser, N., Michalski, A., Scheltema, R.A., Olsen, J.V., and Mann, M. (2011). Andromeda: a peptide search engine integrated into the MaxQuant environment. J. Proteome Res. *10*, 1794–1805. https://doi.org/10.1021/pr101065j.

55. UniProt Consortium (2019). UniProt: a worldwide hub of protein knowledge. Nucleic Acids Res. *47*, D506–D515. https://doi.org/10.1093/nar/gky1049.

56. Tyanova, S., Temu, T., Sinitcyn, P., Carlson, A., Hein, M.Y., Geiger, T., Mann, M., and Cox, J. (2016). The Perseus computational platform for comprehensive analysis of (prote)omics data. Nat. Methods *13*, 731–740. https://doi.org/10.1038/nmeth.3901.

57. Arita, Y., Allen, S., Chen, G., Zhang, W., Wang, Y., Owen, A.J., Dentinger, P., and Sidhu, S.S. (2016). Rapid isolation of peptidic inhibitors of the solute carrier family transporters OATP1B1 and OATP1B3 by cell-based phage display selections. Biochem. Biophys. Res. Commun. *473*, 370–376. https://doi.org/10.1016/j.bbrc.2016.01.050.

58. Tonikian, R., Zhang, Y., Boone, C., and Sidhu, S.S. (2007). Identifying specificity profiles for peptide recognition modules from phage-displayed peptide libraries. Nat. Protoc. *2*, 1368–1386. https://doi.org/10.1038/nprot.2007.151.

59. Gabrielsen, M., Buetow, L., Nakasone, M.A., Ahmed, S.F., Sibbet, G.J., Smith, B.O., Zhang, W., Sidhu, S.S., and Huang, D.T. (2017). A General Strategy for Discovery of Inhibitors and Activators of RING and U-box E3 Ligases with Ubiquitin Variants. Mol. Cell *68*, 456–470.e10. https://doi.org/10.1016/j.molcel.2017.09.027.

60. Temps, C., Lietha, D., Webb, E.R., Li, X.F., Dawson, J.C., Muir, M., Macleod, K.G., Valero, T., Munro, A.F., Contreras-Montoya, R., et al. (2021). A Conformation Selective Mode of Inhibiting SRC Improves Drug Efficacy and Tolerability. Cancer Res. *81*, 5438–5450. https://doi.org/10.1158/0008-5472.CAN-21-0613.

61. Winter, G. (2010). xia2: an expert system for macromolecular crystallography data reduction. J. Appl. Crystallogr. *43*, 186–190. https://doi.org/10.1107/S0021889809045701.

62. Kabsch, W. (2010). Xds. Acta Crystallogr. D Biol. Crystallogr. *66*, 125–132. https://doi.org/10.1107/S0907444909047337.

63. McCoy, A.J., Grosse-Kunstleve, R.W., Adams, P.D., Winn, M.D., Storoni, L.C., and Read, R.J. (2007). Phaser crystallographic software. J. Appl. Crystallogr. *40*, 658–674. https://doi.org/10.1107/S0021889807021206.

64. Emsley, P., Lohkamp, B., Scott, W.G., and Cowtan, K. (2010). Features and development of Coot. Acta Crystallogr. D Biol. Crystallogr. *66*, 486–501. https://doi.org/10.1107/S0907444910007493.

65. Adams, P.D., Afonine, P.V., Bunkóczi, G., Chen, V.B., Davis, I.W., Echols, N., Headd, J.J., Hung, L.W., Kapral, G.J., Grosse-Kunstleve, R.W., et al. (2010). PHENIX: a comprehensive Python-based system for macromolecular structure solution. Acta Crystallogr. D Biol. Crystallogr. *66*, 213–221. https://doi.org/10.1107/S0907444909052925.

66. McCarty, K.S., Jr., Miller, L.S., Cox, E.B., Konrath, J., and McCarty, K.S., Sr. (1985). Estrogen receptor analyses. Correlation of biochemical and immunohistochemical methods using monoclonal antireceptor antibodies. Arch. Pathol. Lab. Med. *109*, 716–721.

YMTHE, Volume *33*

Supplemental Information

Locking CBL TKBD in its native conformation presents a novel therapeutic opportunity in mutant CBL-dependent leukemia

Syed Feroj Ahmed, Jayanthi Anand, Wei Zhang, Lori Buetow, Loveena Rishi, Louise Mitchell, Jonathan Bohlen, Sergio Lilla, Gary J. Sibbet, Colin Nixon, Amrita Patel, Karolina A. Majorek, Sara Zanivan, Jacinta C. Bustamante, Sachdev S. Sidhu, Karen Blyth, and Danny T. Huang

Supplemental Information

Materials and Methods

Estimation of iRFP–CBLock variant concentration in cells

The MOLM-13$^{Luc/CBLock}$ variant cells were induced with 2 µg/ml dox on every 2 days and cells from day 1, 3, 5 and 8 were lysed. The cell lysates (2.0 µl) from three independent experiments from each day for each iRFP–CBLock variant were separated by SDS-PAGE using 4–12%NuPAGE™ Bis-Tris gels (Thermo Fisher Scientific). Purified iRFP–CBLock (25 µM) was run as a standard in each gel. Following separation, the gels were scanned using an Odyssey CLx Imaging System (LI-COR). The bands were quantified using the LI-COR Image Studio 6.0 software and normalized against the whole-cell lysate concentration. The cell pellet for each sample yielded 50 µl whole-cell lysate. Concentration of iRFP–CBLock variant was calculated using the formula:

Concentration (µM) = [{(Normalized band intensity of iRFP–CBLock variant/Band intensity of purified iRFP–CBLock) x Concentration of purified iRFP–CBLock (µM)} x total volume of cell lysate]/ total cell volume.

Volume of $5.5x10^6$ MOLM-13 cells were calculated to be ~10 µl, considering an average diameter of 15 µm for each cell and using the formula $4/3\pi r^3$

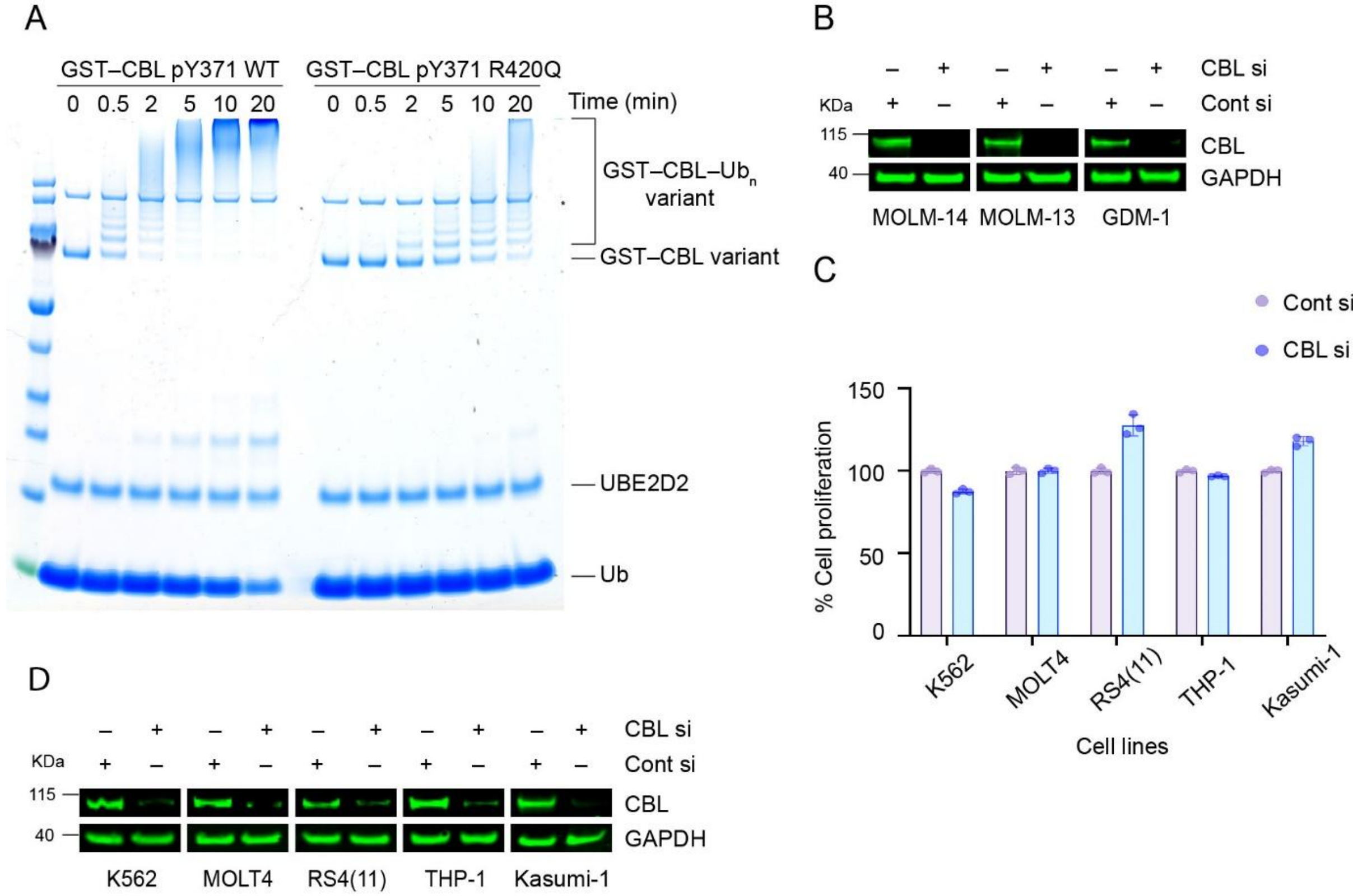

Fig S1. CBL-WT cannot provide growth advantage to leukemia cells. (A) Representative Coomassie stained gel showing the *in vitro* auto-ubiquitination assay performed with purified GST-pCBL 47-435 or GST-pCBL 47-435 R420Q. Three independent experiments were performed. (B) Related to Figure 1D, expression of CBL and GAPDH (loading control) in lysates from MOLM-14, MOLM-13 and GDM-1 cells transfected with control siRNA (Cont si) or CBL siRNA (CBL si). (C) Proliferation of leukemia cells K562, MOLT4, RS4(11), THP-1, and Kasumi-1 knocked down with control siRNA (Cont si) or CBL siRNA (CBL si) and analyzed after 48 hours using Cell Titer-Glo and represented as percentage of cells knocked down with cont si. The data are presented from three independent experiments while the error bars represent ±SD (n = 3 independent experiments). (D) Expression of CBL and GAPDH (loading control) in lysates from K562, MOLT4, RS4(11), THP-1, and Kasumi-1 cell lines transfected with control siRNA (Cont si) or CBL siRNA (CBL si). The GAPDH panel is a loading control in B and D.

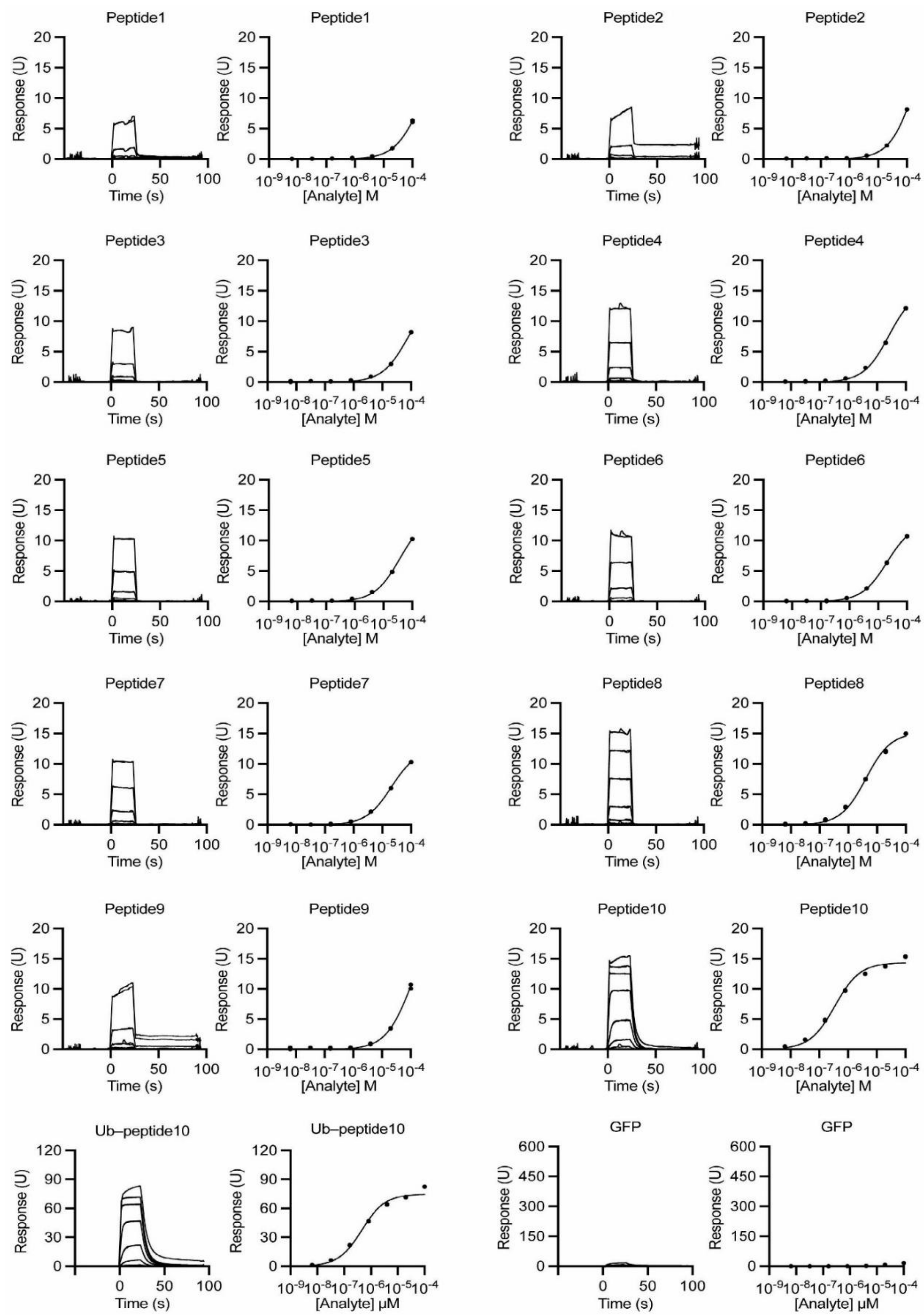
Peptide1
Peptide2
Peptide3
Peptide4
Peptide5
Peptide6
Peptide7
Peptide8
Peptide9
Peptide10
Ub–peptide10
GFP
Response (U)
Time (s)
[Analyte] M
[Analyte] μM

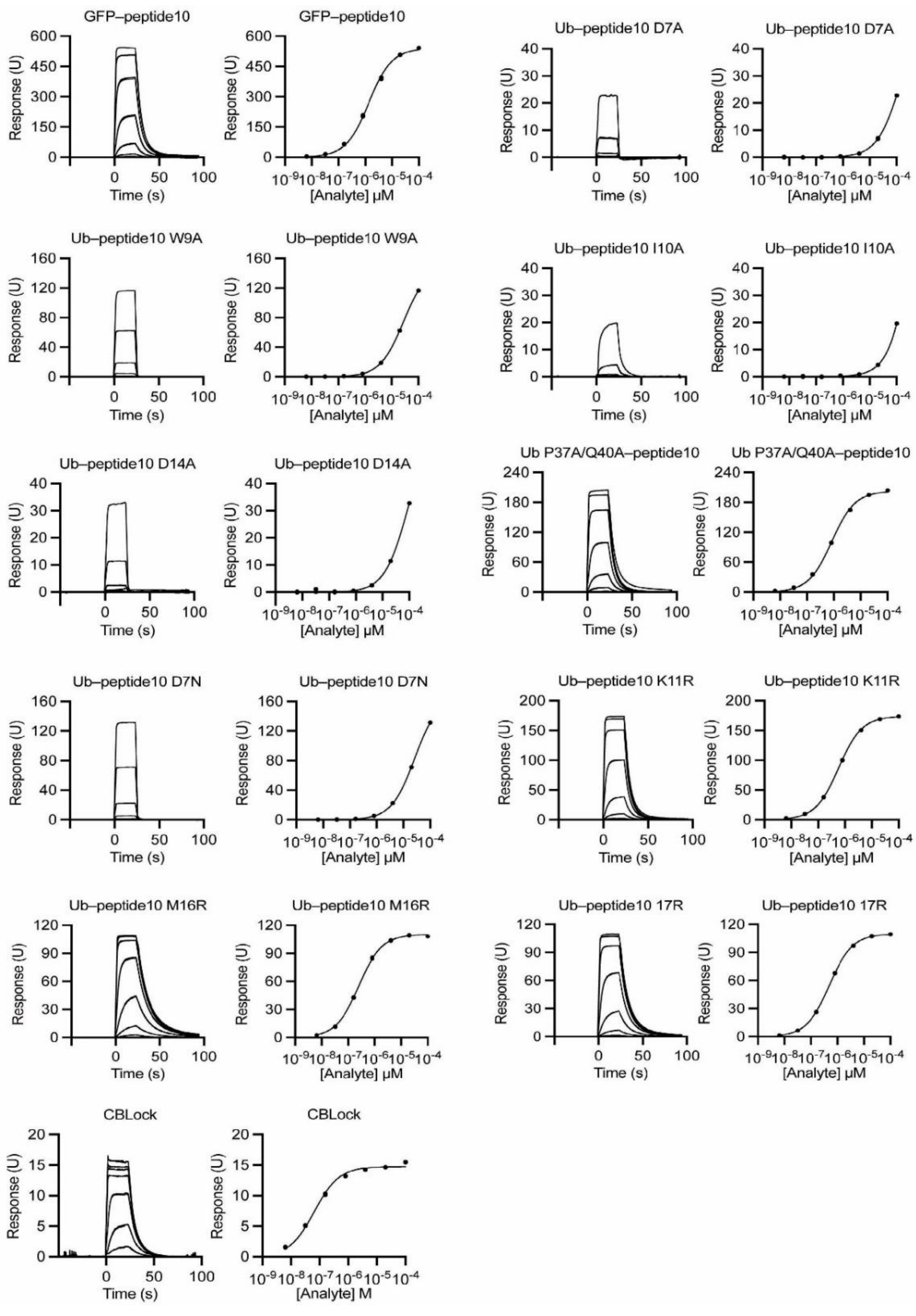

Fig S2. SPR analyses of GST-bound ligand and analyte binding affinities, related to Table 1. Representative sensorgrams (left) and binding curves (right) for GST-TKBD and the analytes peptide (1-10), Ub-peptide10, GFP, GFP-peptide10, Ub-peptide10 D7A, Ub-peptide10 W9A, Ub-peptide10 I10A, Ub-peptide10 D14A, Ub-P37A/Q40A-peptide10, Ub-peptide10 D7N, Ub-peptide10 K11R, Ub-peptide10 M16R, Ub-peptide10 17R, CBLock, as indicated above each sensorgram and binding curve set. Two technical replicates (n =2) for each binding curve.

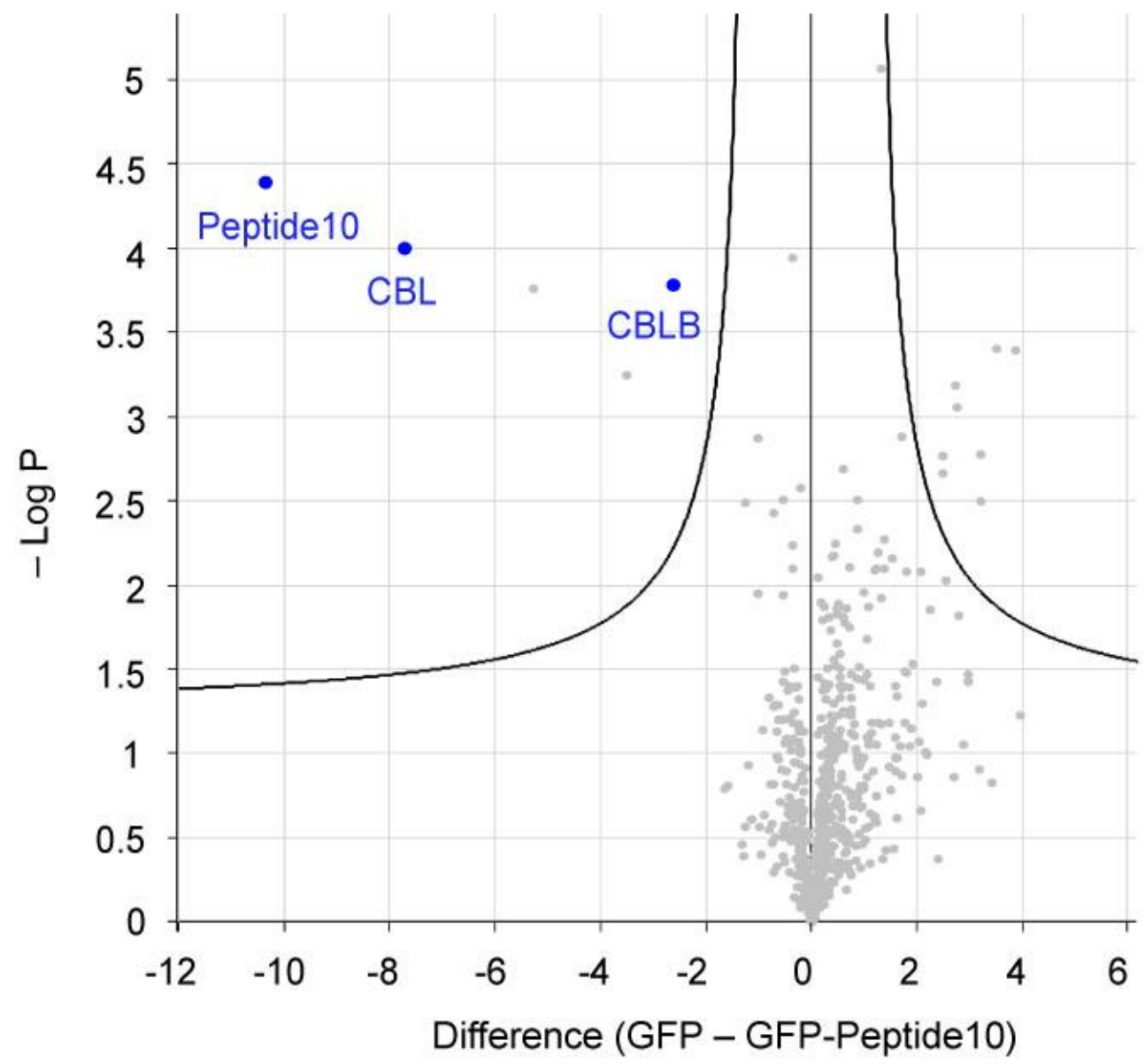

Fig S3. Peptide10 binds specifically to CBL in cells. Volcano plot showing differences in the interactomes of GFP and GFP-peptide10. The experiment was performed in three biological replicates.

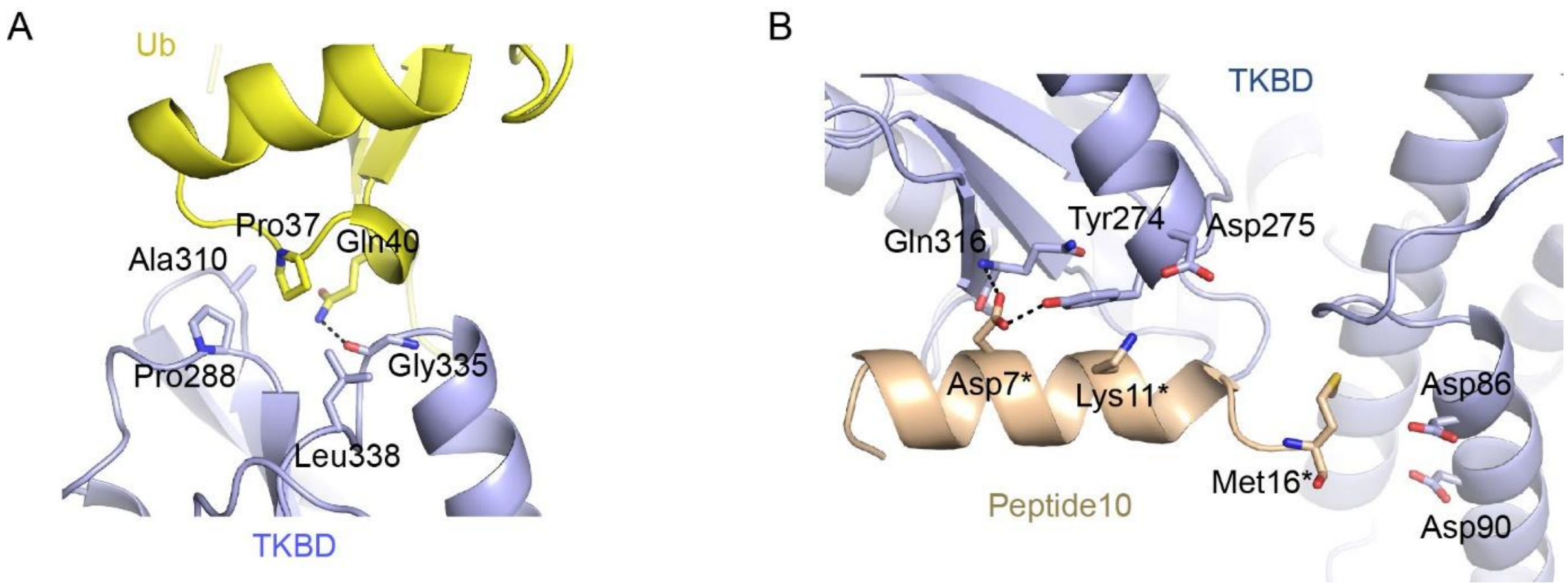

Fig S4. Close-up views of binding interfaces in the CBL TKBD-Ub-peptide10 complex structure (A) Close-up view of CBL TKBD-Ub binding interface. Key interacting residues are shown as sticks. Coloring as in Figure 2A. O and N atoms are colored red and blue, respectively. (B) Close-up view of CBL TKBD-peptide10 binding interface. Residues and regions for potential sequence optimization to improve binding affinity are shown as sticks. Coloring as in Figure 2A. O and N atoms are colored red and blue, respectively.

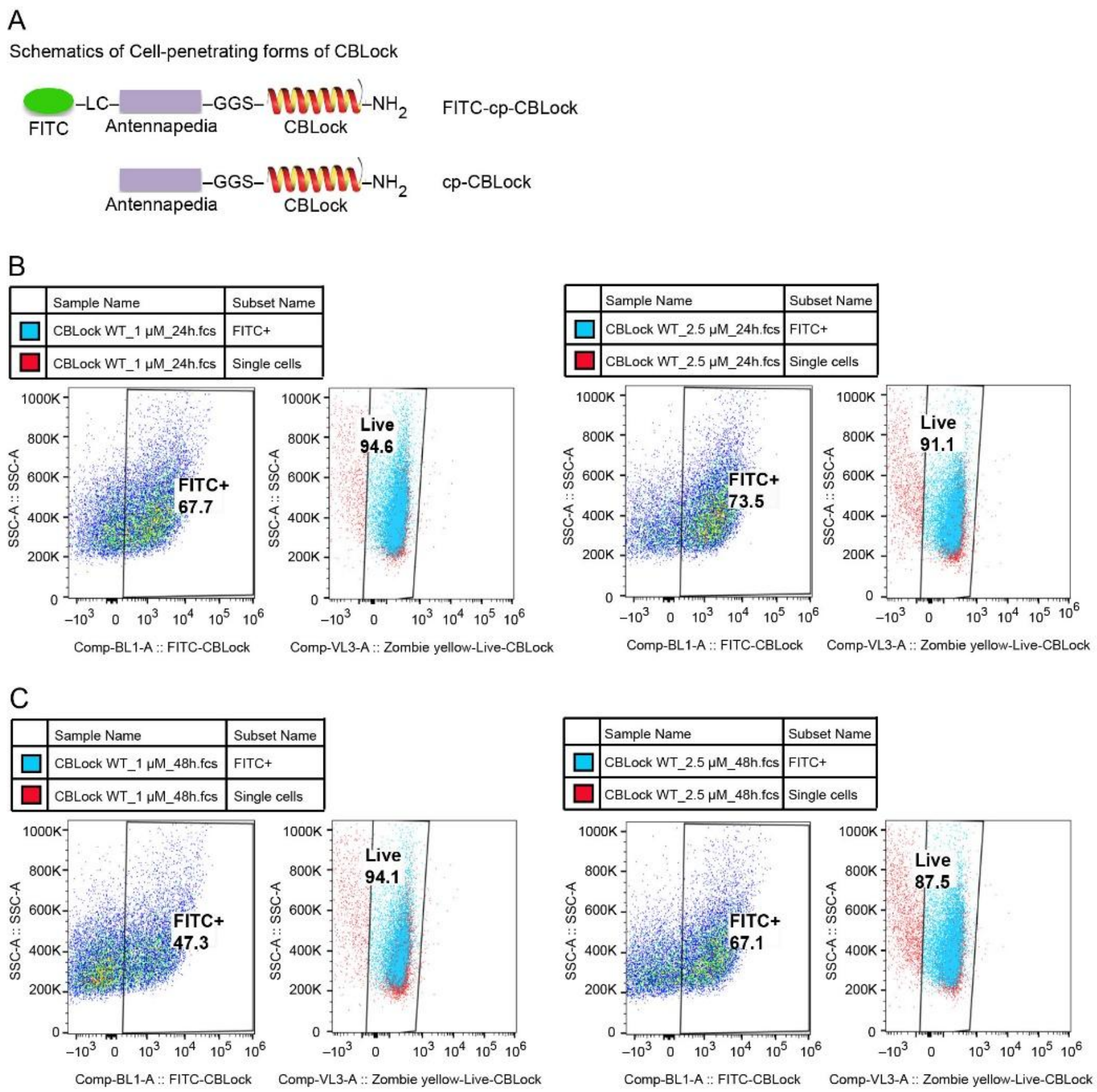

Fig S5. Cell penetrating form of CBLock. (A) Schematics outlining the CBLock with antennapedia (cp-CBLock) with/without FITC conjugation. (B) FACS analysis of MOLM-14 cells treated with FITC-cp-CBLock (1 and 2.5 µM) for 24 hours and 48 hours. Zombie yellow was used to find the dead cells. Non-treated cells were used as fluorescence negative controls to set gating. The experiment was performed in three biological replicates.

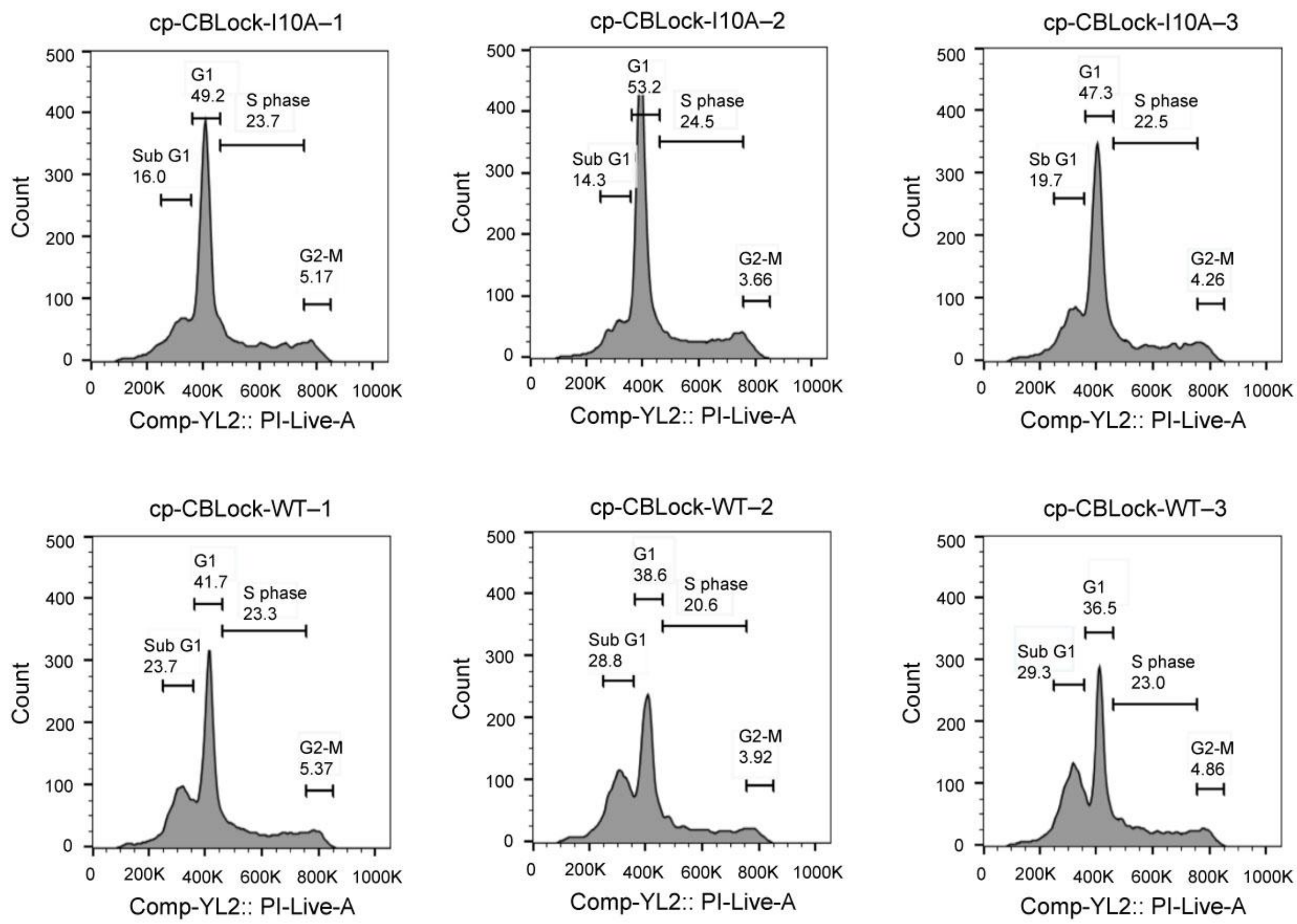

Fig S6. CBLock treatment causes sub-G1 cell cycle arrest in mutant CBL cells. Cell cycle analysis of MOLM-14 cells treated with 2.5 µM cp-CBLock WT or cp-CBLock I10A for 48 hours. Representative examples of data shown in Figure 4B. Vehicle treated cells were used as the controls. Histograms show the cell cycle profile of the cells from the 3 independent experiments stained with PI.

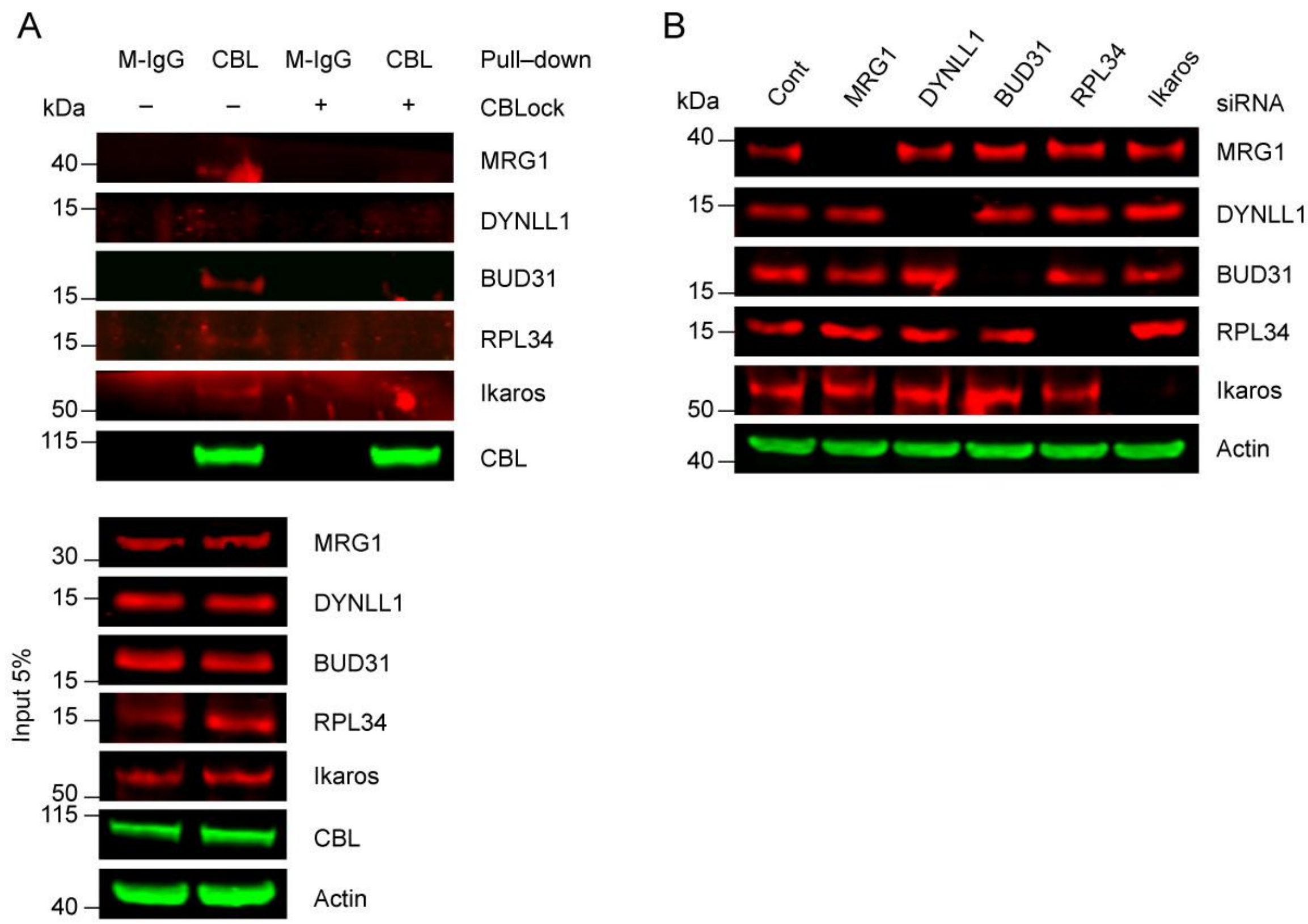

Fig S7. CBLock disrupts additional mutant CBL interactome. (A) Co-immunoprecipitation assay of MOLM-14 cell lysates treated with vehicle (water) or 5 µM cp-CBLock. Anti-CBL antibody and mouse anti-IgG (M-IgG) immunoprecipitants and cell lysates were analyzed by immunoblotting with anti-MRG1, anti-DYNLL1, anti-BUD31, anti-RPL34, anti-Ikaros, anti-CBL and anti-Actin antibodies as indicated. Related to Figure 4H. (B) Related to Figure 4I, expression of MRG1, DYNLL1, BUD31, RPL34, Ikaros and Actin in lysates from MOLM-14 cells transfected with control siRNA (Cont si), MRG1 siRNA (MRG1 si), DYNLL1 siRNA (DYNLL1 si), BUD31 siRNA (BUD31 si), RPL34 siRNA (RPL34 si), or Ikaros siRNA (Ikaros si). The Actin panel is a sample control in A and B.

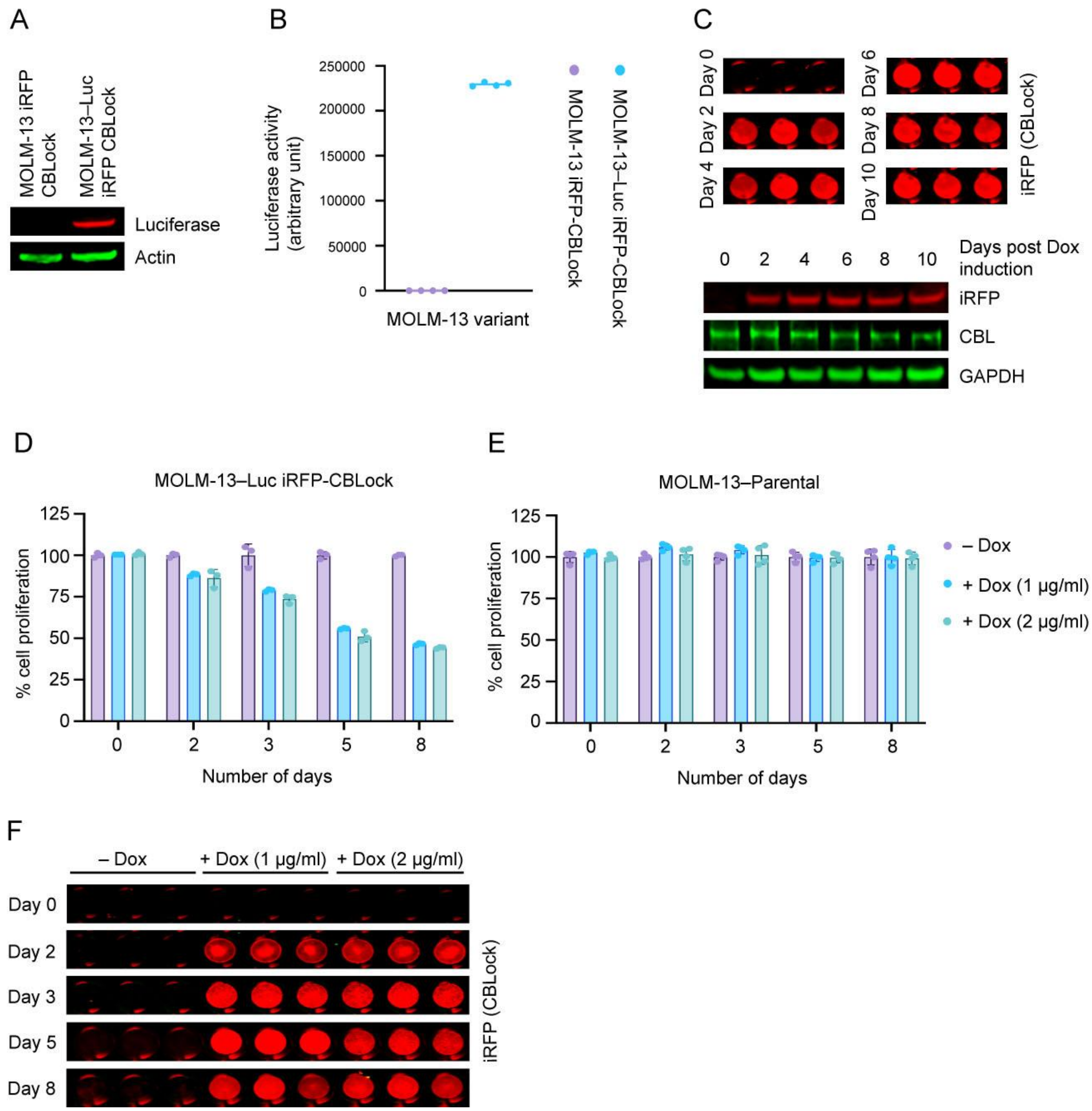

A
MOLM-13 iRFP CBLock
MOLM-13–Luc iRFP CBLock
Luciferase
Actin
B
Luciferase activity (arbitrary unit)
250000
200000
150000
100000
50000
0
MOLM-13 variant
MOLM-13 iRFP-CBLock
MOLM-13–Luc iRFP-CBLock
C
Day 0
Day 2
Day 4
Day 6
Day 8
Day 10
iRFP (CBLock)
0 2 4 6 8 10
Days post Dox induction
iRFP
CBL
GAPDH
D
MOLM-13–Luc iRFP-CBLock
% cell proliferation
125
100
75
50
25
0
0 2 3 5 8
Number of days
E
MOLM-13–Parental
% cell proliferation
125
100
75
50
25
0
0 2 3 5 8
Number of days
– Dox
+ Dox (1 µg/ml)
+ Dox (2 µg/ml)
F
– Dox
+ Dox (1 µg/ml)
+ Dox (2 µg/ml)
Day 0
Day 2
Day 3
Day 5
Day 8
iRFP (CBLock)

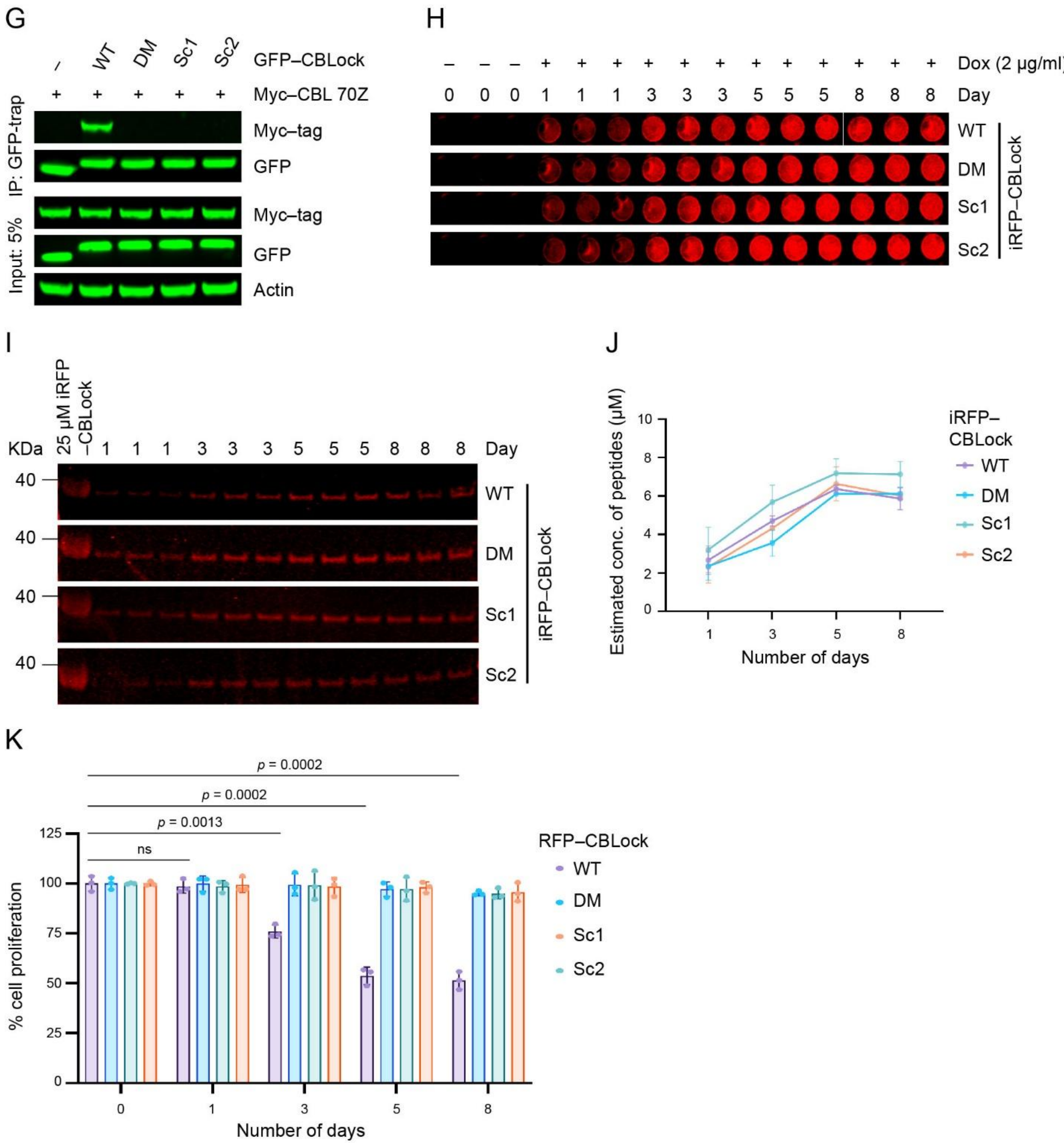

Fig S8. Developing constitutive luciferase expressing and inducible iRFP-CBLock variant expressing MOLM-13 cells. (A) Expression of luciferase and Actin were analyzed from lysates of MOLM-13^{CBLock} or MOLM-13$^{Luc/CBLock}$ cells using anti-luciferase and anti-Actin antibodies, where Actin is the loading control. (B) Luciferase activity of MOLM-13^{CBLock} or MOLM-13$^{Luc/CBLock}$ cells using One-Glo assay system (n=4). The data points are presented from four biological replicates (n = 4 biological replicates). (C) Dox induced expression of iRFP-CBLock. MOLM-13$^{Luc/CBLock}$ cells were treated with 2 μg/ml dox every two days for 10 days. Cells from day 0, 2, 4, 6, 8, 10 were scanned using the LI-COR imaging system. Alternatively, lysates from dox induced cells were lysed and checked for iRFP (CBLok), CBL and GAPDH expressions,

where GAPDH is used as the loading control. (D) Proliferation of MOLM-13$^{Luc/CBLock}$ cells treated with dox (1 and 2 μg/ml) every 2 days for 8 days and cells from 0, 2, 3, 5, and 8 days were analyzed using Cell Titer-Glo and represented as percentage of cells on day 0. The data points are presented from three independent experiments while the error bars represent ±SD (n = 3 independent experiments). (E) Proliferation of MOLM-13^{parental} cells treated with dox (1 and 2 μg/ml) as in D. The data points are presented from four independent experiments while the error bars represent ±SD (n = 4 independent experiments). (F) iRFP (CBLock) expression from cells in D. (G) Co-immunoprecipitation assay of HEK293 cell lysates overexpressing Myc-tagged CBL-70Z along with GFP or GFP-CBLock variants as indicated in the figure. GFP-trap immunoprecipitants and cell lysates were analyzed by immunoblotting with anti-Myc–tag, anti-GFP and anti-Actin antibodies as indicated. (H) iRFP (CBLock variant) expression from MOLM-13^{Luc}cells expressing the respective variant in presence of 2 μg/ml dox from days post induction as indicated in the figure. (I) Dox-induced cells in H were lysed and lysates were checked for iRFP (CBLock variants). Purified iRFP–CBLock (25 μM) was run as a standard. (J) Plot showing the estimated concentrations (in μM) of iRFP–CBLock variants. The data points represent the mean values from three independent experiments while the error bars represent ±SD (n = 3 independent experiments). (K) Proliferation of MOLM-13$^{Luc/CBLock}$ variant cells treated with dox (2 μg/ml) every 2 days for 8 days and cells from 0, 1, 3, 5, and 8 days were analyzed using Cell Titer-Glo and represented as percentage of cells on day 0. The data points are presented from three independent experiments while the error bars represent ±SD (n = 3 independent experiments).

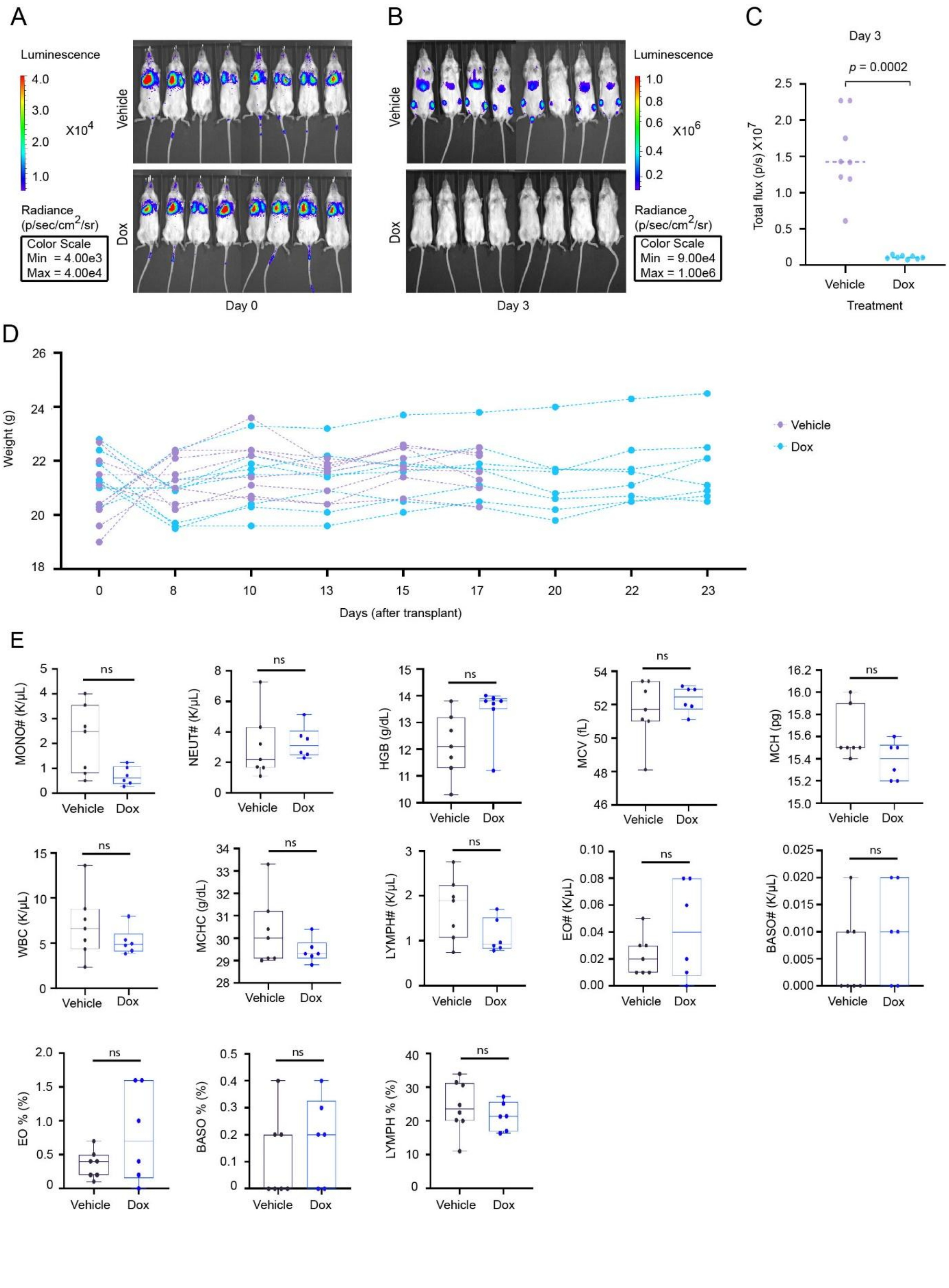
A
Luminescence
4.0
3.0
2.0
1.0
X10^4
Radiance (p/sec/cm^2/sr)
Color Scale
Min = 4.00e3
Max = 4.00e4
Vehicle
Dox
Day 0
B
Vehicle
Dox
Luminescence
1.0
0.8
0.6
0.4
0.2
X10^6
Radiance (p/sec/cm^2/sr)
Color Scale
Min = 9.00e4
Max = 1.00e6
Day 3
C
Day 3
p = 0.0002
Total flux (p/s) X10^7
Vehicle
Dox
Treatment
D
Weight (g)
Days (after transplant)
Vehicle
Dox
E
MONO# (K/μL)
NEUT# (K/μL)
HGB (g/dL)
MCV (fL)
MCH (pg)
WBC (K/μL)
MCHC (g/dL)
LYMPH# (K/μL)
EO# (K/μL)
BASO# (K/μL)
EO % (%)
BASO % (%)
LYMPH % (%)
ns
Vehicle
Dox

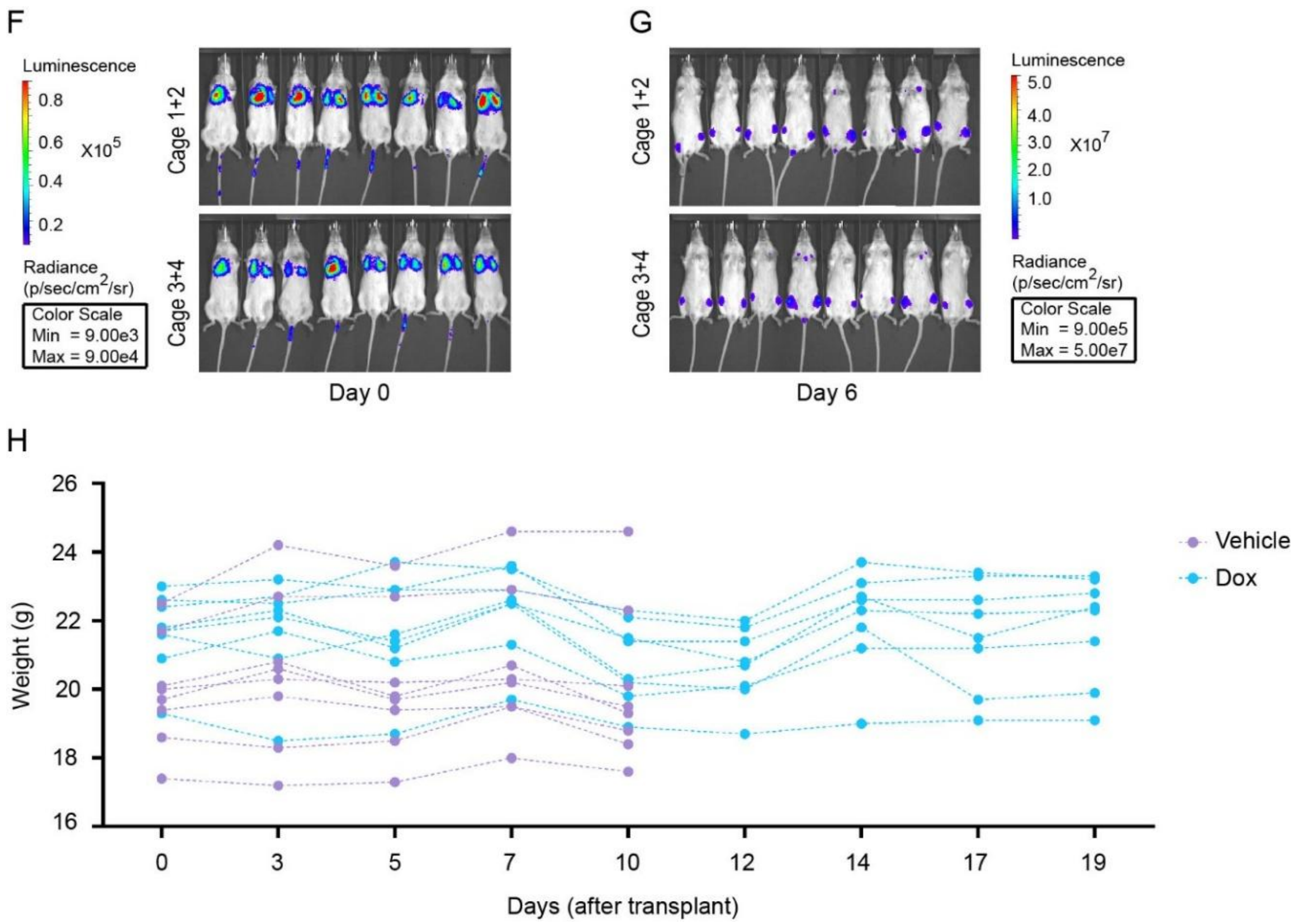

Fig S9. CBLock delays tumour progression in the mouse model of AML. (A) Bioluminescence imaging of NSG mice injected with MOLM-13$^{Luc/CBLock}$ cells on the day of cell injection to monitor cell uptake (n=8 mice per group). Relative to Figure 5B. (B) Bioluminescence imaging of NSG mice iv injected with 100 µl of $5x10^6$ MOLM-13$^{Luc/CBLock}$ cells and treated with either vehicle (n = 8) or dox (n = 8) to regularly track disease progression, related to Figure 5B. (C) Tumor burdens in both groups of mice (shown in B) were measured as total flux (p/s). Total flux (p/s) for the ventral view of the mice were calculated from the region of interest (ROI) of the bioluminescent images of the vehicle (n= 8) and dox (n = 8) treated mice, the dashed lines represent median values. (D) Plot showing body weights of individual mice treated with vehicle (n = 8) or dox (n = 8). (E) Complete blood count analyses of vehicle (n=7) and dox (n=6, reason for exclusion of 2 samples provided in the methods section) treated mice cardiac whole blood. Plots showing neutrophil count (NEUT), monocyte count (MONO), hemoglobin (HGB), mean corpuscular volume (MCV), mean corpuscular hemoglobin (MCH), white blood cells (WBC), mean corpuscular hemoglobin concentration (MCHC), lymphocyte count (LYMPH), eosinophil count (EO), basophil count (BASO), eosinophil percent (EO %), basophil percent (BASO %), and lymphocyte percent (LYMPH %) between vehicle and dox treated mouse blood. Data points represent individual mice blood harvested at clinical endpoint (day12) for vehicle (n = 7) and timed endpoint (day 21) for dox (n = 6) treated mice, the error bars represent ±SD. (F) Bioluminescence imaging of NSG mice injected with MOLM-13$^{Luc/CBLock}$ cells on the day of cell injection to monitor cell uptake. Related to Figure 6F. (G) Bioluminescence imaging of NSG mice iv injected with 100 µl of $1x10^6$ MOLM-13$^{Luc/CBLock}$ cells to track disease progression. (H) Plot showing body weights of individual mice treated with vehicle or dox.

Table S1: Data collection and refinement statistics

	TKBD-Ub-peptide10
**Data collection**	
PDB	9ERZ
Space group	$P12_11$
Cell dimensions	
a, b, c (Å)	58.78, 87.32, 84.35
α , β, γ (°)	90, 99.39, 90
Resolution (Å)	60.2–2.02 (2.05–2.02)
R_{merge} (%)	3.8 (50.8)
R_{pim} (%)	2.4 (33.2)
Completeness (%)	97.2 (99.7)
Multiplicity	3.4 (3.3)
I/σI	17.1 (2.2)
CC(1/2)	0.999 (0.835)
Wilson B (Å^2)	37.85
**Refinement**	
R_{work} (%)	17.81
R_{free} (%)	22.91
Number of reflections	54602
No. atoms	
Protein	6064
Water	349
Ligand /ion	2
R.m.s.d.	
Bond lengths (Å)	0.007
Bond angles (°)	0.768
B-factors	
Protein	55.56
Ca^{2+}	74.77
Water	46.39

Values in parentheses are for highest-resolution shell.
